# Phosphorylation by PINK1 Releases the UBL Domain and Initializes the Conformational Opening of the E3 Ubiquitin Ligase Parkin

**DOI:** 10.1371/journal.pcbi.1003935

**Published:** 2014-11-06

**Authors:** Thomas R. Caulfield, Fabienne C. Fiesel, Elisabeth L. Moussaud-Lamodière, Daniel F. A. R. Dourado, Samuel C. Flores, Wolfdieter Springer

**Affiliations:** 1 Department of Neuroscience, Mayo Clinic Jacksonville, Florida, United States of America; 2 Mayo Graduate School, Neurobiology of Disease, Mayo Clinic, Jacksonville, Florida, United States of America; 3 Department of Cell & Molecular Biology, Computational & Systems Biology, Uppsala University, Uppsala, Sweden; University of California San Diego, United States of America

## Abstract

Loss-of-function mutations in *PINK1* or *PARKIN* are the most common causes of autosomal recessive Parkinson's disease. Both gene products, the Ser/Thr kinase PINK1 and the E3 Ubiquitin ligase Parkin, functionally cooperate in a mitochondrial quality control pathway. Upon stress, PINK1 activates Parkin and enables its translocation to and ubiquitination of damaged mitochondria to facilitate their clearance from the cell. Though PINK1-dependent phosphorylation of Ser65 is an important initial step, the molecular mechanisms underlying the activation of Parkin's enzymatic functions remain unclear. Using molecular modeling, we generated a complete structural model of human Parkin at all atom resolution. At steady state, the Ub ligase is maintained inactive in a closed, auto-inhibited conformation that results from intra-molecular interactions. Evidently, Parkin has to undergo major structural rearrangements in order to unleash its catalytic activity. As a spark, we have modeled PINK1-dependent Ser65 phosphorylation *in silico* and provide the first molecular dynamics simulation of Parkin conformations along a sequential unfolding pathway that could release its intertwined domains and enable its catalytic activity. We combined free (unbiased) molecular dynamics simulation, Monte Carlo algorithms, and minimal-biasing methods with cell-based high content imaging and biochemical assays. Phosphorylation of Ser65 results in widening of a newly defined cleft and dissociation of the regulatory N-terminal UBL domain. This motion propagates through further opening conformations that allow binding of an Ub-loaded E2 co-enzyme. Subsequent spatial reorientation of the catalytic centers of both enzymes might facilitate the transfer of the Ub moiety to charge Parkin. Our structure-function study provides the basis to elucidate regulatory mechanisms and activity of the neuroprotective Parkin. This may open up new avenues for the development of small molecule Parkin activators through targeted drug design.

## Author Summary

Parkinson's disease (PD) is a devastating neurological condition caused by the selective and progressive degeneration of dopaminergic neurons in the brain. Loss-of-function mutations in the *PINK1* or *PARKIN* genes are the most common causes of recessively inherited PD. Together the encoded proteins coordinate a protective cellular quality control pathway that allows elimination of impaired mitochondria in order to prevent further cellular damage and ultimately death. Although it is known that the kinase PINK1 operates upstream and activates the E3 Ubiquitin ligase Parkin, the molecular mechanisms remain elusive. Here, we combined state-of-the art computational and functional biological methods to demonstrate that Parkin is sequentially activated through PINK1-dependent phosphorylation and subsequent structural rearrangement. The induced motions result in release of Parkin's closed, auto-inhibited conformation to liberate its enzymatic functions. We provide for the first time a complete protein structure of Parkin at an all atom resolution and a comprehensive molecular dynamics simulation of its activation and opening conformations. The generated models will allow uncovering the exact mechanisms of regulation and enzymatic activity of Parkin and potentially the development of novel therapeutics through a structure-function-based drug design.

## Introduction

Mutations in the PTEN-induced putative kinase 1 (*PINK1*) and *PARKIN* genes are the most common causes of autosomal recessive Parkinson's disease (PD) [1]. Although the molecular mechanism underlying the pathogenesis of PD remain elusive, it has become clear that PINK1 and Parkin protein functionally cooperate in a novel mitochondrial quality control pathway [2]. Upon depolarization of the mitochondrial membrane, the Ser/Thr kinase PINK1 is stabilized on damaged organelles and plays a pivotal role for the activation and recruitment of the E3 Ubiquitin (Ub) ligase Parkin from the cytosol [3]–[6]. Parkin then labels numerous mitochondrial proteins with the small modifier protein Ub [7], [8]. Upon ubiquitination of mitochondria, adaptor proteins such as p97 and p62 are recruited to facilitate clustering of mitochondria around perinuclear regions and the selective degradation of substrates via the proteasome system and of whole organelles via autophagy (mitophagy) [3], [7], [9], [10]. Mutations in both genes, *PINK1* and *PARKIN*, abrogate this presumably neuroprotective pathway through distinct molecular mechanisms and at different steps along the sequential process [3]–[6].

PINK1 has been demonstrated to phosphorylate Parkin at residue Ser65 in its N-terminal Ub-like (UBL) domain [11]–[13]. Moreover, it has been suggested that the activation of Parkin's enzymatic functions and its mitochondrial translocation are coupled [11], [14], [15]. Very recently, PINK1 has also been shown to phosphorylate the modifier Ub itself at the same conserved Ser65 residue [16]–[19]. Both phosphorylation events appear to be required for full activation of Parkin's enzymatic functions. Parkin had long been classified as a typical Really-Interesting-New-Gene (RING)-type E3 Ub ligase [20] that bridges the interaction between an E2 Ub-conjugating enzyme and a substrate. However, its Ub transfer mechanism has been challenged lately [21]. In fact, Parkin is a member of the RING-in-between-RING (IBR)-RING (RBR) family of E3 Ub ligases that mediate the transfer of Ub by a novel hybrid mechanism [22]. While Parkin binds the E2 co-enzyme with its RING1 domain (similar to RING ligases), it receives the Ub moiety from the E2 co-enzyme onto its active site (Cys431) in an unstable thioester intermediate (similar to Homologous-to-the-E6-AP-Carboxyl-Terminus (HECT)-type E3 ligases). Ub is then further transferred from Parkin onto a lysine residue of a substrate protein [23].

Consistent with its notoriously weak enzymatic activity, several partial crystal structures of Parkin [24]–[27] show a closed, auto-inhibited conformation. Several intra-molecular interactions between individual domains literally fold back Parkin onto itself. An at least 3-fold inhibition prevents charging of Parkin with Ub that is required for its activation and enzymatic functions [28]. It is evident that in order to gain enzymatic activity, Parkin must undergo major structural rearrangements. Ubiquitination enzymes indeed can perform particularly large conformational changes during their catalytic cycles including the remodeling of domain interfaces [29]. Moreover, phosphorylation-dependent exposure of the RING domain [30], [31] or relief of the auto-inhibited structures has been shown for RING and HECT E3 ligases respectively [32].

Here, we set out to provide structural models for human Parkin that would allow release of its auto-inhibited conformation and consequently activation of its E3 Ub ligase functions. We have applied highly accurate molecular modeling methods to provide for the first time an all-atom resolution of human full-length Parkin. Given the suggested auto-regulatory role of the UBL domain [33] and the importance of PINK1 kinase activity for Parkin activation [11]–[13], we have performed molecular dynamics simulations (MDS) to study conformational changes that might be induced by phosphorylation of Ser65. Strikingly, our simulations and calculations of pSer65 *in silico* predict a structural rearrangement of the UBL domain that initiates a sequential release of Parkin's intra-molecular interactions. Along these opening conformations, we have docked the E2-Ub complex, required for Parkin's activation and enzymatic functions. Importantly, the presented computational predictions are consistent with our cell biological studies. We provide the basis for a better understanding of the molecular mechanisms of auto-inhibition and liberation of Parkin's catalytic activity.

## Results

### Modeling of human Parkin structures

**Figure 1:**
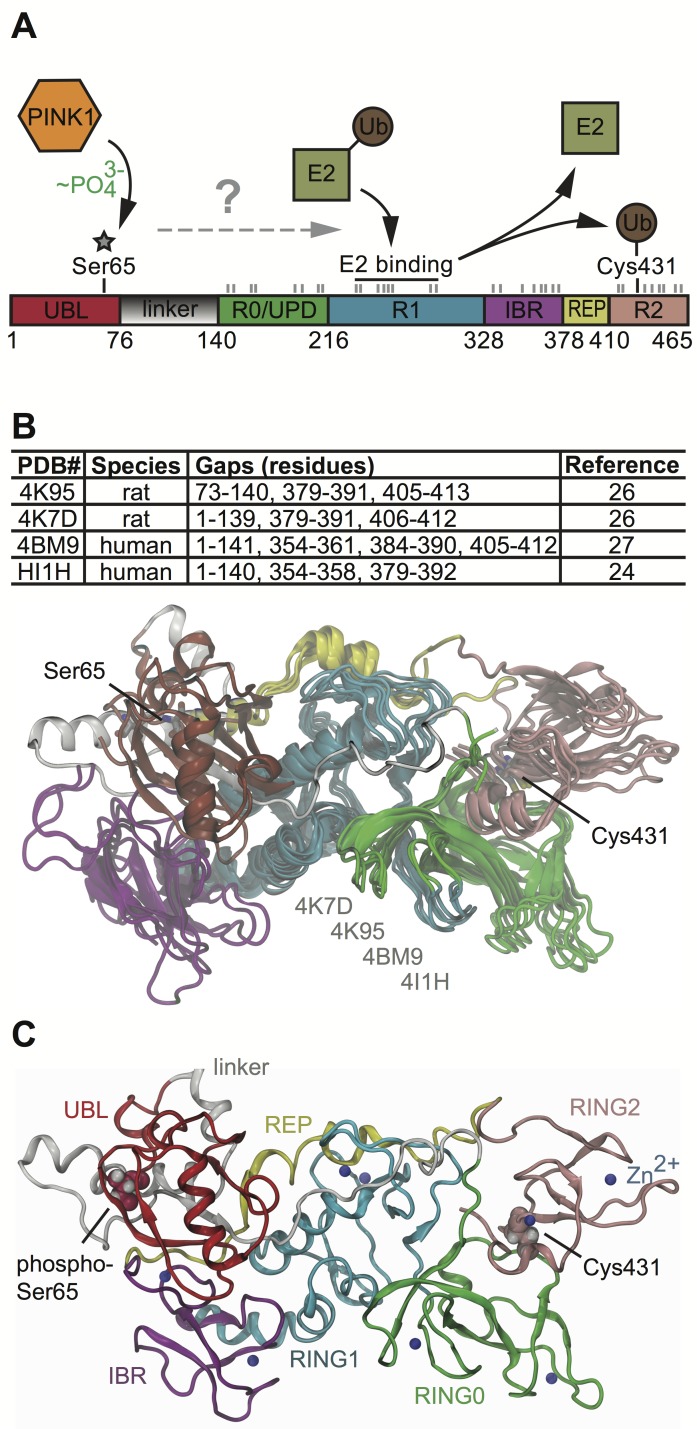
Activation, domains, structures and models of human Parkin. **A**) Schematic representation of Parkin's 2D structure and its activation mode. *Top*: PINK1-dependent phosphorylation of Ser65 has been shown to activate Parkin. During activation, an Ub-loaded E2 enzyme binds Parkin and catalyzes the Ub transfer onto the active site, Cys431, in order to charge Parkin with the small modifier protein. *Bottom*: Shown are color-coded functional domains of human Parkin (residues 1–465): Ubiquitin-like (UBL, red), flexible linker (gray), RING0/Unique Parkin domain (R0/UPD, green), RING1 (R1, blue), in-between RING (IBR, purple), Repressor element of Parkin (REP, yellow), RING2 (R2, pink). The putative E2 binding site in RING1 is indicated by a black line. Gray lines indicate the position of the Zn^2+^ coordinating cysteine/histidine residues in the different RING domains. **B**) Superposition of Parkin's molecular structures. Table lists recently resolved X-ray structures that have been used to generate a model for human full-length Parkin with all-atom resolution. Key residues Ser65 and Cys431 are shown as sticks with carbon in gray, nitrogen in blue, oxygen in red, and sulfur in yellow. **C**) Molecular modeling of Parkin pSer65. The ribbon diagram for an all-atom molecular structure of Parkin is given, presenting an *in silico* model of a PINK1-phosphorylated, and thus activated conformation of Parkin. Color matches that of the domain key indicated, and as above. The phosphorylated Ser65 is shown along with the active site Cys431 as Van der Waals (VdW) spheres colored by domain. Zn^2+^ atoms are shown as blue spheres.

In order to understand the activation mechanisms of the neuroprotective E3 Ub ligase Parkin (for a schematic view of Parkin and its activation see Figure 1A), i.e. its opening conformations and the release of enzymatic function(s), we performed molecular modeling and dynamics simulations. Our models were generated using the recently resolved X-ray structures of human N-terminal truncated Parkin (PDB IDs: 4BM9 [27] and 4I1H [24]) and of its rat homolog (PDB IDs: 4K7D and 4K95 [26]). The molecular modeling methods have been described previously [34]–[36] and the procedure is outlined in the method section in detail. The generated model for the first time allows a view of the full-length human Parkin protein at an all atom resolution (Figure 1B and Movie S1). Most importantly, it provides the structure of the regulatory N-terminus but also closes smaller gaps across Parkin's entire length. The UBL domain (residues 1–76) and in particular a flexible linker (residues 77–140) had not been structurally resolved so far for human Parkin. The linker is comprised of two sub-domains: (1) a semi-globular domain from residues 77 to 125 that appears highly dynamic and (2) a tethering loop region from residues 126 to 140 that connects to the RING0 domain. A further examination of the linker's secondary structural features gives the following: α-helical regions from residues Gly77 to Gly85, Arg89 to Ser108, a helix loop turn from Leu112 to Ser116, and minor beta-roll (anti parallel) from Val117 to Leu123 with the beta-strands from the UBL domain residues Phe4-Asn8 and Leu41-Phe45. From residue Leu123 onward, the linker is random coil through Arg140.

The newly modeled N-terminus consequently alters the position of the IBR relative to the UBL domain and RING1. The superposition overlay deviates slightly from the X-ray structures (Figure 1B), however, root mean square (RMS) measurements between the backbone of the improved model and various collected structure remains under 5 Å for residues Ser145 to Val465. The RING domains contained within are rather rigid due to zinc-finger stabilization, but the UBL domain and the adjacent linker are both flexible and capable of large movement with free MDS (Movie S2). Our complete model of human Parkin plus observations for its dynamic motion indicates that the N-terminal UBL-linker region acts like a spring/clamp that tightly holds Parkin in its closed, auto-inhibited conformation. Noteworthy, a prime role for the N-terminus in negatively regulating Parkin's enzymatic activity had already been established [33]. Consistently, PINK1-dependent phosphorylation of the UBL-located Ser65 appears to play a regulatory role for activation and recruitment of Parkin to depolarized mitochondria [11], [14], [15].

### Phosphorylation of Parkin at Ser65 induces local structural changes

**Figure 2:**
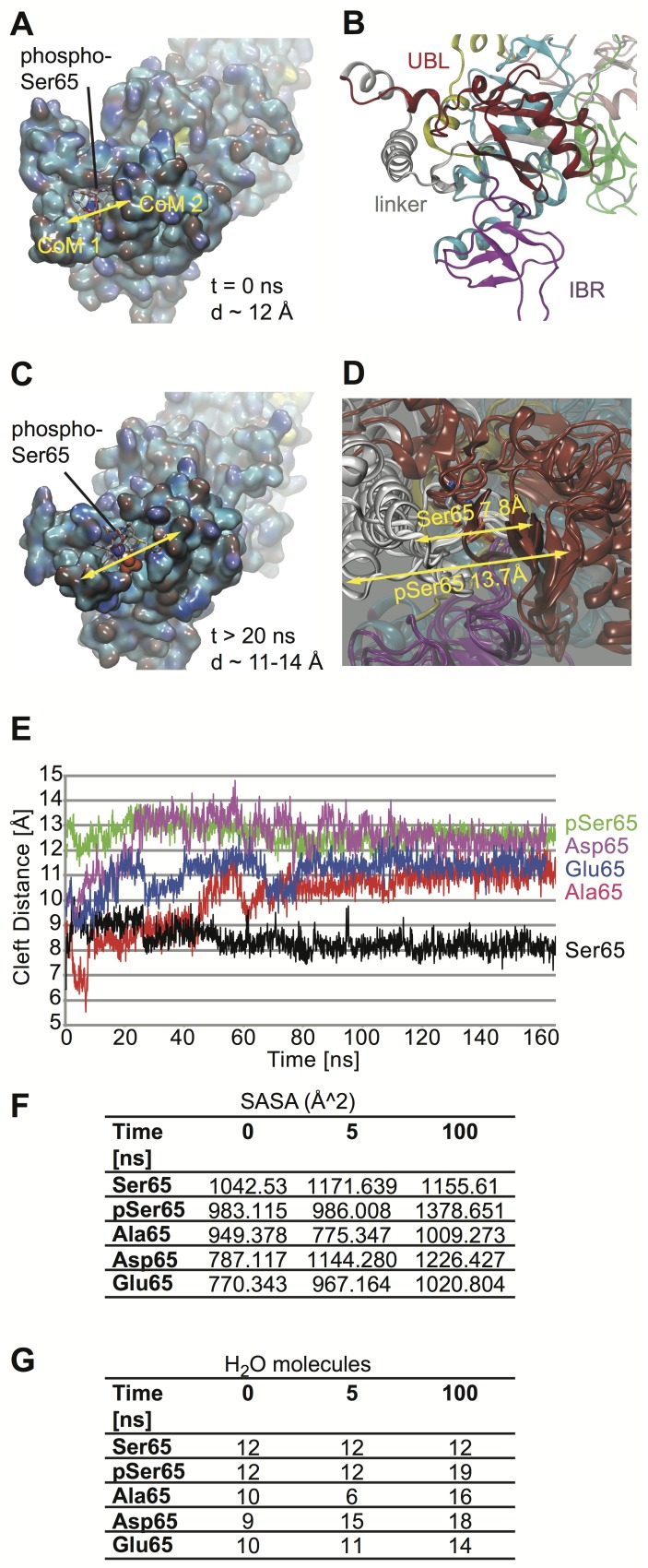
phospho-Ser65 triggers cleft widening in the N-terminal region. **A**) Center view of a newly defined pocket in Parkin with Ser65 positioned towards the middle. The cleft is formed between the flexible linker region [*cleft wall 1*: Arg97, Ser110, Val105, Val111, Leu112, Asp115 Ser116, Val117, and Gly118] and the UBL domain [*cleft wall 2*: Met1, Ile2, Val3, Phe4, Ser19, Leu61, Asp62, Gln63, Gln64, Ile66, and Val67]. Parkin is rendered in solvent accessible surface colored by atom type (nitrogen-blue, oxygen-red, carbon-cyan). Yellow double-sided arrow indicates cleft regions that were used for center-of-mass calculations: CoM1 (Ser110, Val111, Asp115) to CoM2 (Met1, Ile2, Val3, Phe4, Gln63). The initial cleft width is given for time equal zero. **B**) The ribbons diagram for Parkin is shown for comparison with same orientation. Relevant domain labels are given. **C**) Parkin pSer65 is shown after 20 ns of unbiased MDS. The yellow arrow indicates the increased cleft distance. **D**) Superposition of Parkin structures after 100 ns MDS. Shown are structures for Ser65 and pSer65 as well as for S65A, S65D and S65E variants. Arrows indicate the cleft distances of Ser65 and pSer65. **E**) Plot for CoM1 to CoM2 distance over time from MDS. Graph shows a relatively closed and stable cleft for unmodified Ser65 (black) of about 8 Å in distance, while pSer65 (green) shows a much more wider cleft from the start, ranging from 11–14 Å over time. Phospho-mimic mutants S65D (magenta) or S65E (blue) as well as the phospho-dead mutant S65A (red) show a strong increase in cleft size over time reaching distance observed with pSer65. **F**) Solvent-Accessible-Surface-Area (SASA-Å^2^) within the pocket enclosing Ser65 measured in Å^2^ units. While Ser65 maintain a relatively stable SASA, values for pSer65 strongly increase over time. An increase in SASA over time is also observed for the substitutions S65D and S65E as well as for S65A after an initial decrease. **G**) Shown are numbers of H_2_O molecules within the cavity surrounding Ser65 during MDS. While Ser65 constantly maintains twelve H_2_O molecules, pSer65, S65D, S65E, and S65A show a greater solvation of the cavity, consistent with an increased cleft distance and improved SASA.

Given this functional link and the particular N-terminal flexibility, we modeled PINK1-dependent phosphorylation of Parkin's UBL domain at Ser65 *in silico* to investigate potential opening conformations (Figure 1C). In our Parkin model, Ser65 is buried within a pocket that is formed between the flexible linker region and the UBL domain (Figure 2A/B). We found that phosphorylation of Ser65 (pSer65) resulted in conformational changes compared to unmodified Parkin in the static model (Figure S1) as well as in more flexibility during free MDS (Movie S3). A comparison between the pSer65 Parkin model with existing X-ray structures is given in Figure S2. Locally, pSer65 induced a widening of the surrounding cleft and an increase of water molecules occupying the pocket (Figure 2). We used two center-of-mass metrics to determine the cleft widening. Calculations were carried out first on the edges of the cleft on each side (Figure 2A/C, for orientation see Figure 2B). Ser65 showed a rather narrow gap of about only 7–9 Å, however pSer65 induced a widening of the cleft>12 Å. Figure 2D shows the wide range in flexibility between the linker and the UBL domain as the MDS proceeds. Simulations of greater than 100 ns for Parkin Ser65 and pSer65 gave approximately 8 Å or 14 Å, respectively.

To follow up on these significant changes, we studied mutations of Ser65. In functional studies, Serine is often substituted with Alanine for a phospho-dead version (S65A), while mutations to the negatively charged Aspartic (S65D) or Glutamic (S65E) acid are used as phospho-mimic variants. For critical analysis of the intermolecular interactions induced by these mutations (Figure S3), we computed changes in binding energy (ΔΔG), using the Zone Equilibration of Mutants (ZEMu) [37] method, implemented in the MacroMoleculeBuilder (MMB) [38]. However, we could not detect any significant change among the Ser65 substitutions (Figure S3).

We next modeled the Ser65 mutations to measure gap distances during free MDS using two correlative center-of-mass sets (Figure 2D/E). Both S65D and S65E showed an initial gap distance of about 10 Å. The cleft further widened quickly over the course of 20 ns and longer using MDS, reaching distances of around 12–13 Å similar to pSer65 (Figure 2E). The phospho-dead S65A variant displayed an initial narrower gap, rather comparable to unmodified Ser65, but showed a pronounced gap opening during simulation of 20 ns and longer (Figure 2E) reaching widths closer to pSer65 and both phospho-mimic mutants. Moreover, we found that hydration of the pocket interfacing UBL domain and linker provokes the opening of Parkin (Movie S4). Based on these findings, we measured the Solvent-Accessible-Surface-Area (SASA-Å^2^) within the pocket and counted the number of water molecules at common time intervals (from the simulation). The average number of water molecules within the pocket ranges from 9 to 12 for the initial time point (t = 0), 6 to 12 for t = 5 ns, and 12 to 19 for t = 100 ns (Figure 2F). Ser65 allowed the pocket to be filled with a constant number of approximately 12 water molecules during the entire simulation, while pSer65 induced a rapid increase from 12 to 19 water molecules that were maintained over time. In the case of S65A, the pocket initially collapsed from 10 to 6 and then slowly re-hydrated to over 16 water molecules. Both phospho-mimic mutants induced solvation of the pocket surrounding residue 65, more similar to pSer65 Altogether, we provide structural evidence that phosphorylation or mutations of Ser65 perturbs the stability between the UBL domain and the linker region, such that the structure becomes looser more quickly allowing the enclosing cleft to widen.

### Functional cell-based analysis of Ser65 and substitutions

**Figure 3:**
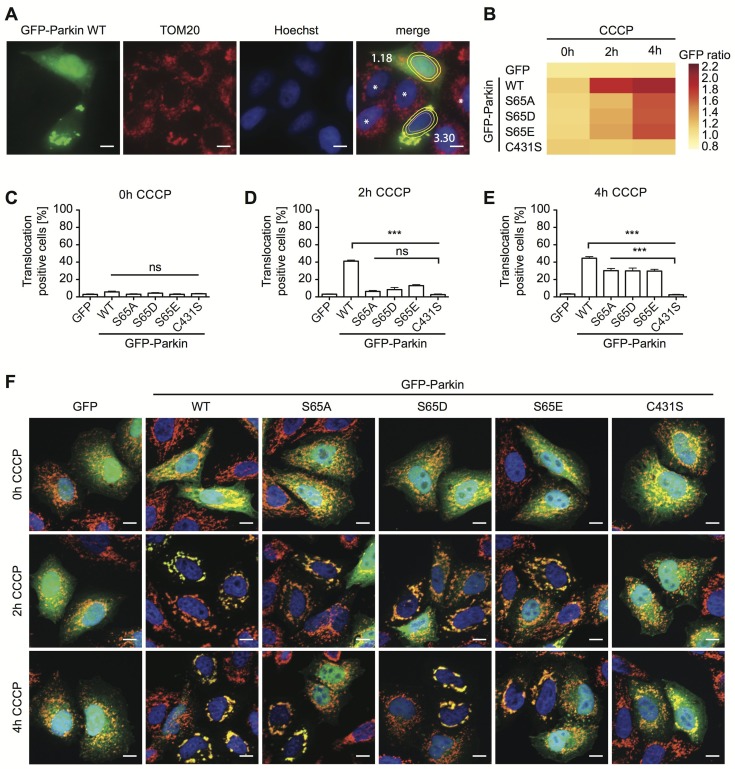
Cell-based high content imaging of Parkin Ser65 mutations. HeLa cells transiently expressing GFP-Parkin wild type, S65A, S65D, or S65E mutations were left untreated (0 h) or treated with the uncoupler CCCP for 2 h or 4 h. Cells expressing GFP only or the catalytically inactive GFP-Parkin C431S mutations served as controls. **A**) Images have been acquired using automated microscopy and show mitochondria (TOM20) in red, GFP-Parkin in green and nuclei (Hoechst) in blue. Quantification of Parkin re-localization is assessed by measuring maximal intensity in a cytoplasmic ring around the nucleus divided by the mean intensity of the nuclear GFP signal. Cytoplasmic ring and inner nuclear regions are schematically shown in the merge image. The GFP ratio is given for each GFP-positive cell in white as an example and reflects Parkin translocation to mitochondria. Untransfected cells or cells below threshold expression of Parkin are marked with a white asterisk and have been excluded from the analysis. **B**) The average GFP ratios of Parkin wild type and Ser65 mutations per well are shown as a heat map. **C–E**) Bar graphs give the percentages of cells with a defined mitochondrial translocation of Parkin at 0 h (**C**), 2 h (**D**), and 4 h (**E**) of CCCP treatment. A GFP threshold of 1.8 was chosen that corresponds to the average ratio of Parkin wild type translocation after 2 h CCCP treatment (n>4 plates with at least 4 wells per condition, one-way ANOVA, Tukey's post-hoc, p<0.0001, F = 147.3, ns – not significant, *** p<0.0005). **F**) Given are representative merge images at higher magnification that show co-localization of GFP-Parkin (green) and mitochondria (anti-TOM20, red). Nuclei (Hoechst) are shown in blue. Scale bars correspond to 10 µM. For images of the individual channels at all time points, see Figure S4.

To corroborate our structural predictions how mutations affect opening of the cleft that encloses residue 65, we expressed GFP-Parkin wild type, S65A, S65D, and S65E variants in human HeLa cells. As a functional readout, we used an unbiased high content imaging assay that monitors mitochondrial translocation of Parkin in cells over time [39]. The paradigm relies on chemical uncoupling of mitochondria by treatment with carbonylcyanide m-chlorophenylhydrazone (CCCP), which induces PINK1-dependent phosphorylation of Parkin at Ser65 in the UBL domain, Ub charging of Cys431 and Parkin's recruitment to mitochondria. Translocation of Parkin is quantified as the ratio of cytoplasmic to nuclear GFP (Parkin) signal for each of the fusion proteins (for an example, see Figure 3A). Un-transfected and low level expressing cells were excluded from the analysis, ensuring comparable GFP intensities across all Parkin variants (Figure S4A). Under basal conditions, Parkin wild type and residue 65 mutants were evenly distributed throughout the cell (GFP ratio ∼1). Upon mitochondrial depolarization (2 h CCCP), the average GFP ratio for Parkin wild type strongly increased, indicating its co-localization with mitochondria (Figure 3B). The catalytically inactive Parkin mutant C431S did not translocate to mitochondria, consistent with previous reports that enzymatic activity and mitochondrial translocation of Parkin are linked [11], [14], [15]. In contrast, Parkin S65A, S65D, or S65E did not abrogate translocation, but showed a clear delay in recruitment at both time points analyzed.

We defined translocation positive cells as a GFP ratio of>1.8, which corresponds to the average value (∼40% of the cells) for Parkin wild type at 2 h CCCP (Figure 3C–E). All Parkin mutations showed a significantly reduced percentage at 2 h CCCP treatment with no significant difference between Ser65 substitutions and the control mutant C431S. Ser65 mutants remained less translocation-positive at 4 h CCCP compared to wild type, but showed significantly more translocation compared to C431S. Representative merged immunofluorescence images are given in Figure 3F (for individual channels, see Figure S4B–D). The delay in Parkin activation and translocation caused by mutations of residue 65 is further indicated by a reduced co-recruitment of the Ub adaptor protein p62 (Figure S4C–D). Upon Parkin-dependent ubiquitinations of mitochondrial substrate proteins, p62 promptly accumulates on mitochondria in order to facilitate organelle clustering and degradation [3], [9]. Noteworthy and consistent with our structural analysis of Ser65 mutations, phospho-dead and phospho-mimic substitutions equally delayed Parkin translocation to mitochondria. Though S65A cannot be phosphorylated by PINK1, it is capable of opening the cleft between UBL domain and linker region, similar to pSer65 or phospho-mimic mutants. This is in agreement with previous reports that phosphorylation of Ser65 plays an important role, but is not absolutely required for the translocation of Parkin [13], [16].

### pSer65-induced movement of the UBL-linker region across and alongside Parkin

**Figure 4:**
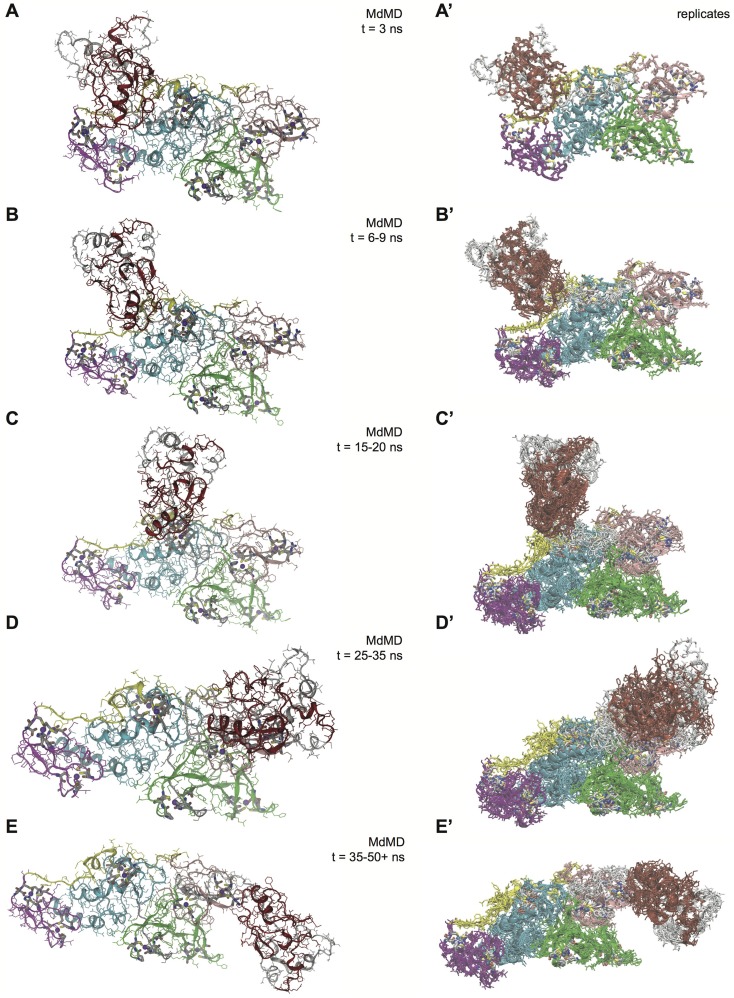
MdMD excursions along the pathway of the UBL domain across Parkin. Representative pathways via MdMD excursions between LC-MOD/MC generated conformers for the movement of the N-terminal region (residues 1–140) in MDS state 1 (panel **A**) to the active site region in MDS state 5 (panel **E**). Panels **A–E** and **A′–E′** represent the five key points from the guideposts that MdMD was able to drive the structures toward. RMSD between the structural model and the guideposts were within 3 Å in each case. Color-coded ribbon structures are given. **A**) The initial structure for Parkin is mostly unperturbed after 3 ns of MdMD sampling with global variable based on LCMOD sampling between generated Parkin conformers. **B**) Opening of the N-terminal region is shown after>10 ns of MdMD sampling. **C**) Midpoint for the UBL domain movement towards the active site region following>15 ns of MdMD sampling. The linker helix region is more dynamic, exposing the E2 binding site in RING1. **D**) UBL domain adjustment as it approaches the C-terminal region after 30 ns of MdMD sampling. **E**) The UBL domain is in final position and occupies region around the active site (Cys431) after 35 ns of MdMD sampling. **A′–E′**) Superposition of the four replicates from MdMD that match the relative time point/stage from panels **A–E**. Ensemble of structures from replicates gives a common relative pathway between guideposts generated conformers.

To model putative opening structures, Parkin models were analyzed using both traditional MDS and enhanced sampling MDS techniques to generate a large pool of conformers. We used Targeted Molecular Dynamics or Maxwell's demon Molecular Dynamics (MdMD) [35], [36], plus mixed torsional sampling with large-scale, low-mode sampling and Monte Carlo Molecular mechanics (e.g. LC-MOD/MCMM) within Schrödinger [40]–[43]. We found several unique conformations that captured the N-terminal flexibility, including some large-scale rearrangements in the positioning of the UBL domain (Movie S3). Retaining only the lowest energy conformers generated from a large ensemble of states, we applied Polak-Ribiere conjugate gradient for structural minimization to obtain the optimal internal coordinates to each structures to arrive at final conformer snapshots. We then dubbed evenly spaced conformers as guideposts spanning the conformational extremes for the UBL domain starting adjacent to the IBR and ending in close proximity to the active site. Using MdMD minimal biasing method, we tested whether or not the Parkin structures could move between the conformers generated with global variable (accessor) based on root mean square deviation (RMSD) to the backbone C-alpha atoms. This revealed a smooth pathway for the movement of the UBL domain (Figure 4 and Movie S5). At the beginning, Parkin showed only minor rearrangements of the UBL domain (Figure 4A), while the movement of the UBL and of the flexible linker is evident at longer times of MDS (Figure 4B–E).

We repeated this simulation using a randomizer in our molecular dynamics sprint interval that ensures non-duplicative runs, and show the superposition of replicates alongside (Figure 4A′–E′ and Movie S6). Using the smooth transitions, we have generated>32,000 conformers to study the opening of Parkin. As a consequence of the flexibility in the linker region, we found an array of domain reorientations that connect in a logical fashion the movement of the UBL domain from its initial configuration to a set of states near the active site (Movie S5). We tested each structure generated with YASARA's What-If and Procheck for backbone dihedrals, rotamers, and packing that support the structures stability during simulations [44]–[46]. The average plot for Phi-Psi space is shown in the Ramachandran plot with Z-axis for dihedral count (Figure S5). The initial MdMD was not applied during the first 1–10 ns, however upon engaging the algorithm, the linearity of the MdMD algorithm measured by RMSD between the replicate and guidepost structure is given (Figure S6). We measured the C-terminal RMSD for residues 145–465 finding three replicates within 2.75 Å of the initial production run. We used an RMS global variable within MdMD to determine a series of conformers related to UBL motion (Figure S7). In contrast to the zinc-finger stabilized C-terminal core (residues Ser141-Val465), the N-terminal region (residues Met1-Arg140) showed a rapidly growing root mean square fluctuation (RMSF) (Figure S8) and an increase in RMSD (Figure S9) in the MdMD replicates.

### Release of Parkin's inhibitory intra-molecular interactions

During the trajectory of the UBL domain across and alongside Parkin, several structural changes were noted that are potentially relevant for E2 co-enzyme binding and Ub charging of Parkin. Following phosphorylation of Ser65 as a trigger, we found that residues within the linker region undergo repeated contacts with the RING1 and RING2 domains during movement of the UBL domain (Movie S5). Our data indicates that several safety belts must be released in order to unleash its E3 ligase activity.

**Figure 5:**
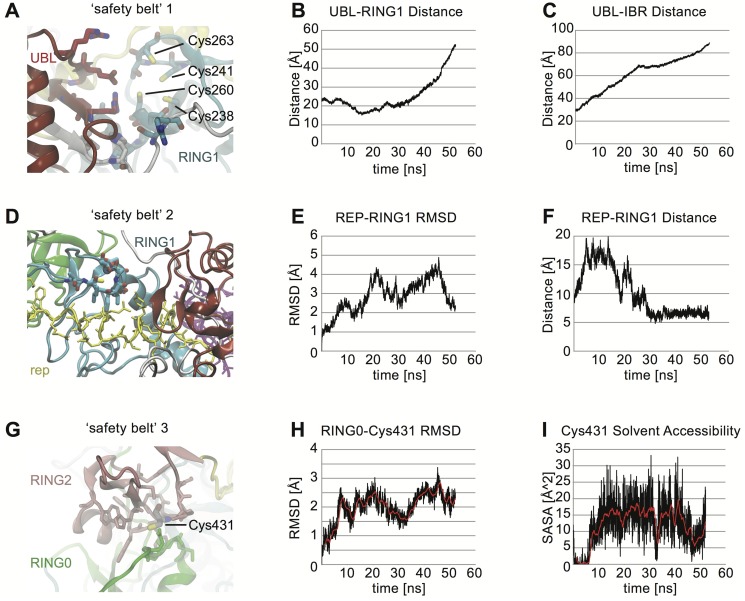
Phosphorylation of Ser65 releases the safety belts of Parkin. **A**) Zoom into safety belt 1: The UBL blocks RING1 and IBR domains. Key cysteine residues of the E2 binding site in RING1 are indicated. The E2 binding site was defined as follows: Ile236, Thr237, Cys238, Ile239, Thr240, Cys241, Thr242, Asp243, Val244, Arg245, Ile259, Cys260, Leu261, Asp262, Cys263, Phe264, His265, Leu266, and Tyr267 **B**) The distance between UBL domain (Leu26) and RING1 (Cys238) significantly increased over time MDS. **C**) Similarly, the distance between UBL (Leu26) and IBR (Phe364) domains significantly increased over time MDS. **D**) Zoom into safety belt 2: The REP region blocks the E2 binding site in RING1 (as defined in A). **E**) Dynamic change in REP-RING1 interaction during Parkin opening motion. Graph shows the release of the REP region from the E2-binding site in RING1 as measured by RMSD. The RING1 is released from the REP region by MdMD time of 20 ns, exposing the E2 binding site. **F**) Loosened interaction between the center Tyr391 in REP region and Cys238 in RING1. The distance increases from 10 to 20 Å. During longer simulations, the distance eventually collapses as the UBL domain moves away and E2 binding has transiently occurred. Across many replicates, we find that the availability of adequate space for an E2 enzyme to approach the binding site in RING1 begins somewhere between 5–22 ns. **G**) Zoom into safety belt 3: Cys431 is buried by RING0. **H**) Release of the active site (Cys431) from RING0 (Arg163 C-alpha atom) as measured by RMSD for center-of-mass. RMSD increases moderately over time indicative of a less compacted area. **I**) SASA for Cys431 entire residue. During MDS, more water is available to Cys431, indicating its enhanced exposure.

First, the UBL-linker region must dissociate from RING1 and IBR domains in order to loosen the entire structure (Figure 5A). Based on our simulations, we measured the release of the inhibitory N-terminus that acts like a spring/clamp. The distance between the UBL and RING1 domains indeed increased from about 20 Å to more than 50 Å over time MDS (Figure 5B). Similarly, the distance between the UBL domain and the IBR region significantly increased from an initial 30 Å to almost 90 Å (Figure 5C).

Second, the repressor element of Parkin (REP), a region between IBR and RING2, blocks access of an Ub-loaded E2 co-enzyme (E2∼Ub; the tilde symbol is used to indicate a thioester bond) in RING1 (Figure 5D). This inhibitory interaction is loosened during Parkin's transitions from stage 1 (Figure 4A) to stages 2/3 (see Figure 4B/C). To measure this release, we calculated RMSD scores. The REP residues considered were all within 5 Å of the supposed RING1 binding region (defined as Cys238, Thr240, Cys241, and Cys263). MdMD showed a steady increase of the RMSD score from 0 Å to>5 Å over the course of 50 ns sampling (Figure 5E). We then measured the distance of the REP element to the E2 binding site in RING1. The bond distance from Tyr391 (REP region) to Cys238 (E2 binding site in RING1) changed over time from under 10 Å to ∼20 Å (Figure 5F). This might allow access of an incoming E2∼Ub complex to the binding site. Interestingly, during continued UBL-linker movement towards the active site (as seen in Figure 4), Tyr391 is eventually able to reposition back into the E2 binding site, possibly indicating a reset mechanism for the next binding event of a (re-)charged E2 enzyme.

Third, the RING0 domain buries Parkin's active site Cys431, making it unavailable to receive Ub from an incoming E2 (Figure 5G). During the UBL-linker movement, RMSD measurements indicate that the RING0 to Cys431 slightly increased (∼3 Å) (Figure 5H), while the distance itself initially increased during the first 10 ns from 15.8 Å to ∼19 Å and then decreased to under 17 Å (data not shown). SASA calculations indicated that Cys431 initially lost virtually all water interaction surface (first 5 ns), but abruptly began to hydrate thereafter (Figure 5I). This coincides with the binding of the E2 co-enzyme on the other side of Parkin at RING1 that had been blocked by the REP region before. For the remainder of the measurements, Cys431 stayed at an average SASA of 12.5 Å^2^ with transient peaks of 20 Å^2^. This increase in solvent exposure over time is indicative of a conformational reorientation, which could allow the active site Cys431 to receive a thioester-bonded Ub moiety from the E2 enzyme.

Taken together, our MDS and subsequent calculations revealed a sequential release of Parkin's safety belts preventing its activation. The dissociation of the inhibitory N-terminus is triggered by PINK1-dependent phosphorylation of Ser65 in the UBL domain. As a consequence, Parkin's entire structure is loosened and further perpetuates the liberation of Parkin's presumed E2 binding region and of its active center as pre-requisite for enzymatic activity.

### E2∼Ub complex – Parkin docking following MdMD

**Figure 6:**
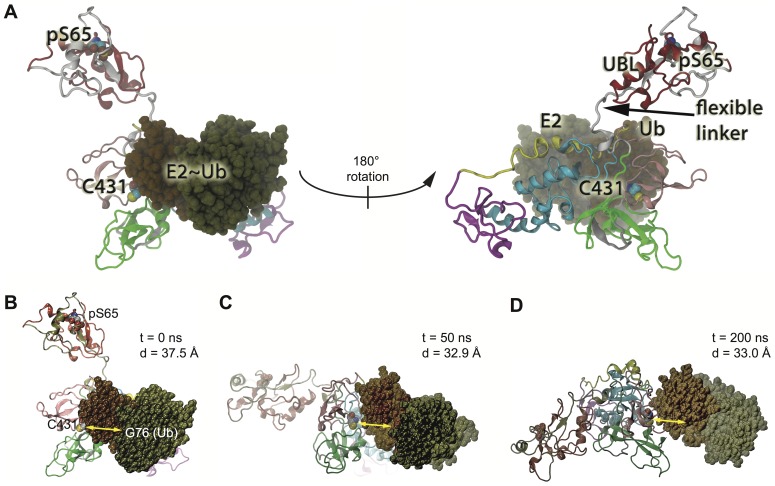
Protein-protein docking for Parkin and a charged E2∼Ub complex. **A**) Ten conformations spanning closed Parkin to fully opened Parkin (see Figure 4) were sampled for protein-protein interactions. The E2-Ub complex was docked at the midpoint UBL position (state 2/3) when the REP region liberated the binding site in RING1 (Figure 4B/C). This conformation showed fewer steric clashes and lowest energy profile. The docking in the same position is shown and rotated 180° to reveal the other side. Residues of the Ubch5a-Ub complex are indicated by color (dark green and brown, respectively). **B–D**) Docking at the RING1 interface is critical for E2-Ub progression towards the active site of Parkin. E2 binding at RING1 limits the UBL-linker mobility preventing the drift back to the original, auto-inhibited state. **B**) Same orientation as in A (left side) predicted as an optimal docking conformation after 0 ns of MDS. The distance between Gly76 of Ub and Parkin's active site (Cys431) is indicated. **C**) Following unbiased MD (>200 ns), the E2-Ub complex moves towards the Parkin's active center. The decreased distance is shown after 50 ns. **D**) Overall re-orientation of the UBL domain and the E2-Ub complex is shown after 200 ns.

To identify the E2 enzyme binding site in RING1 and to dock an E2∼Ub complex, we scanned across an evenly spaced distribution of ∼50 structures spanning the opening of Parkin using Piper for protein-protein docking [43]. We used the structure of an Ub-loaded E2 enzyme UbcH5a/UBE2D1 (PDB code: 4AP4) that shows an isopeptide amide linkage between the mutant active site of the E2 (Cys85Lys) and Gly76 of Ub (UbcH5-Ub complex) [47]. Members of the UbcH5 family have been shown to charge Parkin with Ub and act as co-enzymes during mitophagy [14], [21], [39], [48]. Each Parkin structure was allowed an attempt to dock with the UbcH5a-Ub complex retaining the best ten conformers from each pairing. The resulting pool was then filtered for lowest energy structures using Schrödinger's Bioluminate/Piper docking and Molecular Mechanics-Generalized Born Surface Area evaluation [43], [49]–[51]. The optimal bound state of Parkin with the E2 enzyme is shown in Figure 6A. In this structure (state 2/3, see Figure 4B/C), the REP region is pushed back to better reveal residues in RING1 critical for E2 binding.

The top performing structures were further studied with unbiased (free) MDS. Following docking of the UbcH5a-Ub complex with Parkin, we completed simulations where the Ub-loaded E2 makes substantial movements toward the active site region of Parkin (Figure 6B–D and Movie S7). The distance from residue Cys431 to the C-terminal Ub residue Gly76 ranges from an initial 60 Å (in the closed conformation) to approximately 30 Å after 200 ns of accelerated MDS [52] using default parameters within the NAnoscale Molecular Dynamics engine [53]. The optimal binding pair kept that distance within 40 Å. It is interesting to note, that the E2-Ub complex rolls around Parkin, thereby moving the Gly76 of the bound Ub moiety into a better position for Parkin's catalytic center. While the UbcH5a-Ub complex moves across Parkin, it maintains a final average distance of approximately 32 Å from the thiol of Parkin's Cys431 to Gly76 of Ub. However, without release of the Ub moiety from the E2 (due to the amide linkage in this structure) the co-enzyme stalled in the vicinity of Parkin's active site, while at the same time the UBL domain moved away from the midpoint configuration (state 3, see Figure 4C) into a new conformation unobserved in MdMD (Figure 6A–C, Movie S7). As a control, we started a simulation with the sub-optimal binding for Ub-loaded E2 and Parkin (higher energy). In this case, we found that the docked UbcH5a-Ub complex tended to stall and gradually drifted from the active site toward the IBR domain, increasing the distance between Ub-Gly76 and Parkin-Cys431 (>65 Å) (Movie S8). In summary, our MDS provide the basis to study major domain rearrangements within Parkin as well as to investigate binding of E2 co-enzymes and Ub charging of Parkin as part of its activation process.

### Ub charging of Parkin and utilization of E2 co-enzymes

The labile nature of the E2∼Ub thioester makes the structural and functional studies of these complexes very challenging. For Parkin, a thioester-bound Ub could not be identified so far [21]. A substitution of the active site cysteine in E1, E2, or E3 ubiquitinations enzymes with a serine residue, results in the formation of a relatively stable oxyester bond to Gly76 of Ub. To confirm the suitability of a C431S substitution as a tool to monitor the E2-dependent Ub charging of Parkin, we used MMB and ZEMu. In contrast to many Zn^2+^ coordinating cysteines, the catalytic residue Cys431 is not making any intra-molecular interaction (Figure S10). For the C431S substitution we obtained similar ΔΔGs from three Parkin crystals that indicate no major change, corroborating the usefulness of this particular inactive variant.

**Figure 7:**
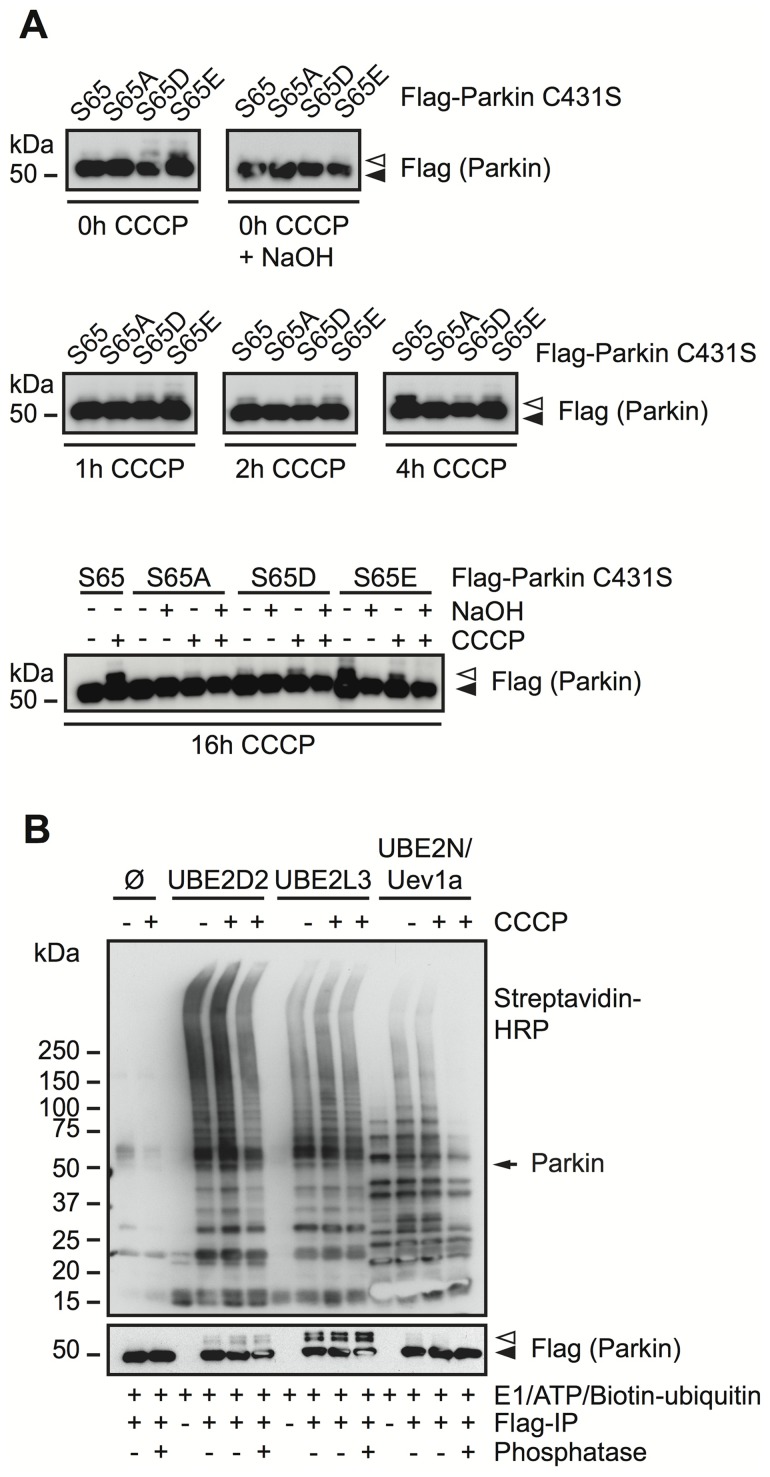
Activation and enzymatic function of Parkin. **A**) HeLa cells were transfected with FLAG-Parkin C431S. Cells were treated with CCCP for 0, 1, 2, 4, or 16 h and harvested. Western blots were prepared to monitor the formation of a Parkin C431S-Ub oxyester, which, in contrast to unmodifed Parkin (closed arrowhead) appears as a band shift (open arrowhead) and is sensitive to NaOH treatment. The phospho-mimic mutations S65D or S65E showed some levels of Parkin C431S-Ub even in the absence of CCCP, consistent with a slight activation under steady-state conditions. **B**) HEK293E cells were transfected with FLAG-Parkin wild type and left either untreated or were treated with 10 µM CCCP. FLAG immunoprecipitations were performed and ubiquitinations reactions were carried out on the beads. All ubiquitination reactions contained E1, ATP, and N-terminally biotinylated Ub. Either no E2 enzyme, or UBE2D2, UBE2L3 or UBE2N plus its co-factor Uev1a were added. In order to analyze the effect of Parkin phosphorylation, some FLAG immunoprecipitates were pretreated with phosphatase before Ub reaction was carried out. UBE2D2, UBE2L3 and UBE2N can serve as co-factors for Parkin *in vitro*. CCCP treatment is not required, but enhances the ubiquitination reactions. Phosphatase treatment reduces the ubiquitinations to the extent observed without CCCP treatment. UBE2N shows no detectable activity towards Parkin auto-ubiquitination. A closed arrowhead indicates the position of unmodified FLAG-Parkin, an open arrowhead labels Ub modified species. An arrow labels the molecular weight of Parkin on the Streptavidin-HRP blot.

To investigate effects of Ser65 mutations on Parkin C431S-Ub oxyester formation as a surrogate for its activation, we expressed these Parkin variants carrying an additional C431S mutation (Figure 7A). As expected, CCCP treatment of cells expressing the single C431S (S65) mutation induced the formation of an 8 kD shifted Parkin band. The specificity of this band was determined by NaOH treatment that chemically cleaves the C431S-Ub oxyester bond. This is consistent with Parkin's auto-inhibited, inactive structure before and its activation upon mitochondrial depolarization. Of note, for both phospho-mimic mutations (in particular S65E and to a lesser extent S65D), an 8 kD shifted band was detected even at basal conditions (i.e. without CCCP treatment). Consistently, S65E (and S65D) showed enhanced levels of Ub-charging at early time points (1 h CCCP) while oxyester formation of S65 Parkin became apparent only after 2 h and strongly increased by 4 h of CCCP treatment. In contrast, the phospho-dead S65A mutant showed no discernable Ub-oxyester at 0 h CCCP and compared to Ser65 strongly reduced levels after longer incubation times with CCCP (16 h). These results are consistent with a slight activation of the phospho-mimic Parkin mutants at steady state and a strongly diminished Ub-charging of the phospho-dead variant S65A.

To provide further evidence that Ser65 phosphorylation plays a role for Parkin's enzymatic function, we performed immunoprecipitation coupled to an *in vitro* ubiquitination assay. We transfected HEK293E cells with FLAG-Parkin and affinity purified Parkin by anti-FLAG from cells that have been treated with CCCP for 1 h or left untreated. Given the importance of PINK1 kinase activity and phosphorylation of Parkin's Ser65 [11]–[13], we treated some samples with phosphatase to observe its E3 ligase activity with or without this activating posttranslational modification. Immunoprecipitated Parkin was incubated with a complete mix of ATP, recombinant Ub and E1 enzyme as well as different E2 enzymes. While incubation with UbcH5b/UBE2D2 or UbcH7/UBE2L3 resulted in the formation of likely mono- and di-ubiquitinated Parkin species, Ubc13/UBE2N together with its co-factor Uev1a was unable to generate these Ub modifications on Parkin (Figure 7B). Parkin, without any E2 enzymes, was not able to Ub modify itself. No discernable differences in Parkin's (auto-)ubiquitination were observed between samples that had been left untreated or had been treated with CCCP or phosphatase. CCCP treatment resulted in enhanced poly-Ub levels as judged by streptavidin detection of the biotinylated Ub used. Phosphatase treatment strongly reduced the enzymatic activities of Parkin. It is unclear whether these ubiquitinations were formed on Parkin itself (but are not detectable with a anti-FLAG antibody) or on E2 enzymes or were attached to other co-immunoprecipitated proteins. Taken together, our structural and functional data corroborate an important role of Ser65 phosphorylation and provide a mechanistic insights into unleashing Parkin through unfolding, E2 enzyme binding and Ub charging as well as activation of its enzymatic E3 ligase functions and concomitant recruitment to damaged mitochondria.

## Discussion

Based on several recent but partial X-ray crystals [24], [26], [27] we performed molecular modeling to provide an all atom resolution structure of human Parkin. This neuroprotective RBR-type E3 Ub ligase is inactivated in familial and probably also sporadic forms of PD [1], [54]. Under basal conditions, Parkin is auto-inhibited through several intra-molecular interactions. Our models suggest that the N-terminal UBL domain and the linker region, which has been crystallographically difficult to resolve, act as a spring/clamp that holds Parkin in its closed conformation. Previous studies had shown that upon mitochondrial stress, Parkin's activation and recruitment is dependent on the upstream kinase PINK1. We describe the first comprehensive molecular dynamics study of Parkin activation upon PINK1-dependent phosphorylation of Ser65 in the UBL domain. We have generated over 30,000 snapshot structures of Parkin that illustrate a sequential release of its intertwined domains along an unfolding pathway to liberate its Ub ligase function(s).

Functionally, PINK1-dependent phosphorylation of Parkin's Ser65 appears as the most upstream event of its activation cascade. We have analyzed a phospho-dead S65A mutation of Parkin as well as the phospho-mimic variants S65D and S65E. Using a high content imaging approach, we found a significant delay in Parkin recruitment upon mitochondrial uncoupling for all Ser65 substitutions, in line with previous studies [13], [55]. In contrast to an earlier report [11], [15], we noted a substantial increase in Ub charging (oxyester formation) of particularly S65E Parkin and to a lesser extent of the S65D variant even in the absence of CCCP. Our findings are in agreement with more recent studies that support the idea of a partially released auto-inhibition for phospho-mimic Parkin mutants [55], [56]. In fact, phospho-mimic Parkin, compared to wild type, showed increased Ub ligase activity at steady state in neuronal cells [55] and *Drosophila* [56] as evidenced by reduced levels of Parkin substrates under physiological conditions. However, phospho-dead Parkin showed greatly diminished Ub charging at all conditions, consistent with a reduced E2 discharge and E3 ligase activity *in vitro* [57] and *in vivo* [56]. Despite the enhanced enzyme activity at base line, neither S65D nor S65E Parkin could be found on mitochondria without depolarization. Although mitochondrial translocation and Ub charging of Parkin appear as interdependent events [11], [14], [15], our data corroborate the hypothesis of a second mechanism that is required for the translocation of Parkin, consistent with the finding that Parkin phosphorylation is not sufficient to trigger its recruitment. Thus, PINK1 phosphorylation of Parkin appears to primarily boost its enzymatic activity, thereby regulating not only mitochondrial function but also activity and survival of dopaminergic neurons [56].

In order to bridge the gap between static structures and enzymatic functions, we performed MDS and used in addition to normal modes, Monte Carlo algorithms and minimal-biasing methods that provided consequent opening conformations of Parkin. Although some of the presented models are hypothetical in nature, it is certain that the N-terminal region has to dissociate from the remaining part of Parkin in order to facilitate further required structural rearrangements. Our full-length Parkin model shows that Ser65 is located within a newly defined cleft that is formed between the UBL domain and the adjacent linker region. Using free MDS, we demonstrate that phosphorylation of Ser65 results in widening and enhanced solvation of this pocket. Similarly, both phospho-mimic and albeit slower also phospho-dead Ser65 mutations allow opening of the cavity as opposed to unmodified Ser65. Our MDS studies further suggest that phosphorylation of Ser65 in Parkin initializes the dissociation of the UBL domain (within ns) and thereby induces larger scale conformational motions over time. It should be noted that the simulation time scale is not an exact match for what is actually occurring in the cell, but is idealized. In the cell, Parkin protein will require more than the calculated time to undergo major structural rearrangements and binding of the E2 co-enzyme in order to become fully active. pSer65-dependent hydration of the surrounding cavity induces the release of the UBL domain, which appears to act as the first safety belt that keeps Parkin's activity in check under basal conditions. Upon dissociation of the UBL domain, we next detected release of the inhibitory REP region from the E2 binding site in RING1 that could facilitate association of an Ub-charged co-enzyme. As a further consequence we also observed conformational adjustments in RING0 that intervenes between RING1 and RING2 burying Parkin's active site when unmodified. In its inactive state, the E2 binding site and Parkin's catalytic center are separated by a distance of more than 50 Å, which would not allow for Ub transfer. However, during opening of Parkin, we measured significant changes in distances, RMSD and SASA values, indicating differences in positioning and solvation of the region surrounding Cys431 that would be important for charging of Parkin.

These promising measurements prompted us to dock an Ub-charged E2 enzyme (UbcH5a/UBE2D1) during the opening phase of Parkin. Indeed, we found conformations that were able to accommodate the co-enzyme and allowed us to generate an energetically favorable Parkin:E2-Ub complex. Strikingly, subsequent free MDS, showed a reorientation of the E2 to better position the Ub-Gly76 residue towards Parkin's active site. This coincides with hydration around Cys431 and results in a significantly reduced distance between the catalytic centers of the E2 and Parkin. The amide linkage in the E2-Ub co-crystal prevented further insights into the transfer of the Ub moiety from the E2 enzyme onto Parkin's active center, but we noted a repositioning of the REP region back into the E2 binding site in RING1. This putative reset mechanism may allow dissociation of the discharged E2 for the next binding event during consecutive ubiquitinations rounds. During mitophagy, multiple E2 enzymes are utilized by Parkin for Ub charging, mitochondrial translocation and substrate ubiquitinations [14], [39], [48], [57], [58]. We have recently demonstrated that some regulate Parkin's activation and mitophagy through redundant, cooperative, or antagonistic mechanisms [39]. E2 enzymes usually confer the linkage specificity for RING-type Ub ligases while HECT- and RBR-type E3 ligases are charged with Ub and themselves define the respective Ub linkages that are formed. Parkin appears to catalyze the conjugation of various Ub modifications ranging from (multi-) mono-ubiquitination to poly-Ub chains with different topologies [59], while particularly K27, K48, and K63 linked chains have been observed during mitophagy [3], [7], [55]. In the absence of Parkin co-crystals with co-enzymes and/or substrates, it will be important to dock additional E2 co-enzymes, other co-factors as well as substrates, where structures are available to shed more light onto the catalytic activity(ies) of Parkin.

In this context, it is important to note that Parkin has been suggested to self-associate through a PINK1-dependent mechanism upstream of its mitochondrial translocation [14]. Our own findings (unpublished) and other recent studies [19], [56] including the crystal structures (PDB IDs: 4K7D and 4K95) [26] support the dimerization/multimerization capability of Parkin. A small fraction of PINK1-phosphorylated (i.e. activated) Parkin could activate non-phosphorylated Parkin in trans and thereby amplify its E3 ligase activity through an autocatalytic mechanism [19], [56]. The inactive C431S Parkin variant would inhibit this feed-forward loop and consistently is unable to translocate to mitochondria [11], [14], [15], [39] suggesting that the Ub moiety must be passed onto a lysine residue of a substrate that may well include Parkin itself. Of note, the delay in S65A translocation to mitochondria was rescued in the presence of wild type (i.e. pSer65) Parkin [19]. However, S65A Parkin could not be charged by wild type in the C431S-Ub oxyester experiment [56]. Accumulating evidence indicates that pSer65 primarily boosts Parkin's enzymatic E3 ligase activity, but also suggests additional functions for the phosphorylation of the UBL domain than release of auto-inhibition [57]. A deletion mutant of Parkin lacking the UBL domain was able to translocate to mitochondria [3], [8], but could not ubiquitinate the model substrate Miro1 while retaining robust auto-ubiquitination and E2 discharge comparable to wild type Parkin [57]. A role of the UBL domain in binding to substrates and regulators has been described [60].

Strikingly, it has been shown that PINK1 also phosphorylates the modifier Ub itself at the conserved Ser65 residue [16]–[19], in addition to Parkin's UBL domain. Of note, Parkin phospho-Ub alone (i.e without CCCP treatment) can activate Parkin wild type, ΔUBL as well as the S65A mutant *in vitro* [16], [17]. However, optimal activation of Parkin appears to depend on both Ser65 phosphorylation events catalyzed by PINK1 [17]. Consistent with a putative phospho-binding site in Parkin's RING0 that has been identified through co-crystallization of a sulphate ion [27], Parkin can bind to phospho-mimic Ub which seemed to be dependent on Parkin pSer65 [18]. One might speculate that while phosphorylation of UBL domain could (pre-)activate Parkin's E3 ligase functions, phosphorylation of Ub that is already attached to a mitochondrial substrate might induce its translocation and full enzymatic activity. Binding of Parkin to phosphorylated Ub moieties (as free or attached monomers or poly-Ub chains) or even its own phosphorylated UBL domain [16]–[19] may also help to maintain an open, active conformation. In summary, our structural and functional data underscore the importance of PINK1-dependent phosphorylation of the UBL domain for the activation of Parkin's enzymatic functions. In addition to functional studies that are required to entirely elucidate the mechanisms and the sequence of events during activation and translocation of Parkin, molecular dynamics simulations will certainly be useful to provide structural insights. Our models highlights multiple opportunities for analysis of PD mutations and modifications to hopefully open up new avenues for the design of safe small molecule activators of this multipurpose neuroprotective E3 Ub ligase.

## Materials and Methods

### Modeling Parkin structures and refinement

The protein sequence of the human E3 Ub ligase Parkin (isoform 1) (Parkin), encoded by the *PARK2* gene, was taken from the NCBI Reference Sequence: NP_004553.2. The following 465 amino acid residues (full-length) were used for modeling: MIVFVRFNSSHGFPVEVDSDTSIFQLKEVVAKRQGVPADQLRVIFAGKELRNDWTVQNCDLDQQSIVHIVQRPWRKGQEMNATGGDDPRNAAGGCEREPQSLTRVDLSSSVLPGDSVGLAVILHTDSRKDSPPAGSPAGRSIYNSFYVYCKGPCQRVQPGKLRVQCSTCRQATLTLTQGPSCWDDVLIPNRMSGECQSPHCPGTSAEFFFKCGAHPTSDKETSVALHLIATNSRNITCITCTDVRSPVLVFQCNSRHVICLDCFHLYCVTRLNDRQFVHDPQLGYSLPCVAGCPNSLIKELHHFRILGEEQYNRYQQYGAEECVLQMGGVLCPRPGCGAGLLPEPDQRKVTCEGGNGLGCGFAFCRECKEAYHEGECSAVFEASGTTTQAYRVDERAAEQARWEAASKETIKKTTKPCPRCHVPVEKNGGCMHMKCPQPQCRLEWCWNCGCEWNRVCMGDHWFDV.

Parkin has several conserved domains to be modeled (from the N-terminus to the C-terminus): UBL (residues 1–76), undefined linker region (residues 77–140), RING0 (or UPD) domain (residues 141–216), RING1 domain (residues 217–328), IBR domain (residues 329–378), REP region (residues 379–410), and RING2 domain (residues 411–465). The Parkin sequence was aligned, with each domain modeled as a separate unit built into a composite structure (see Text S1 – Part 1).

The modeling was built as a hybrid model from consensus between the programs PRIME (Prime v3.0, Schrödinger, LLC, New York, NY) [44], [61], YASARA SSP/Homology/PSSM Method [44], [62]–[67], and TASSER [68]–[73]. The variable loops and gaps were filled using knowledge-based homology and knowledge-based potentials with YASARA, or *ab initio* approach of ORCHESTRAR [74]. Each missing loop was modeled using the Loop Search module implemented in Sybyl 8.0 or with YASARA loop modeler [44], [45], [63], [75], [76]. Only loops with the highest homology and lowest root mean square deviations were selected for the final models. The side chains and rotamers were adjusted with knowledge-based potentials, simulated annealing with explicit solvent, and small equilibration simulations using YASARA's refinement protocol and verified by WHAT-IF and PROCHECK [77]. Fragments were divided into overlapping groups between the five templates (see Text S1 – Part 2). Combined fragments were overlaid using in-house superposition algorithms to determine optimal overlay and energies, which left the extraneous overlaid residues to be discarded as unnecessary. Finally TASSER was considered for each fragment and the entire length protein.

Refinement of the fragments was completed using YASARA's refinement module. These refinements started with the Secondary Structure Prediction (SSP) feature of YASARA. Both homology and fold recognition were considered and a final refinement with the entire model was completed using YASARA for 250 ps of MD using knowledge-based force fields. Additionally, YASARA supports an extensive and large loop library for modeling loops and gaps. The sequence and identity of each fragment was reasonable (see Text S1 – Part 2) [44]. The superposition and subsequent refinement yielded an optimal model for the full-length human Parkin protein. Recently released X-ray structures for large portions of Parkin greatly increased the accuracy of the modeling. The final model was subjected to energy optimization with PR conjugate gradient with an R-dependent dielectric.

The model conformation was verified with WHAT-IF and PROCHECK and has a valid conformation consistent with good phi-psi space [46], [78], [79]. Atom consistency was checked for all 465 amino acids, verifying correctness of chain name, dihedrals, angles, torsions, non-bonds, electrostatics, atom-typing, and parameters. Each model was exported to the following formats: Maestro (MAE), YASARA (PDB). Model manipulation was done with Maestro (Macromodel, version 9.8, Schrödinger, LLC, New York, NY, 2010), or Visual Molecular Dynamics (VMD) [80].

### Molecular dynamics simulation

MDS was completed on each model for conformational sampling, using methods previously described in the literature [35], [36], [81], [82]. Briefly, each Parkin system was minimized with relaxed restraints using either Steepest Descent or Conjugate Gradient PR, and equilibrated in solvent with physiological salt conditions, as shown in the literature. Following equilibration each system was allowed to run MD calculations between 100–1000 nanoseconds in length. The primary purpose of MD for this study was conformational variability that may occur in the UBL. We also conducted conformationally enhanced sampling with MD biasing methods, like MdMD and MC-based generators. The protocol for refinement include the following steps: (1) Simulated annealing with explicit water molecules and ions, (2) Energy minimization, (3) MDS for 500 ps to relax to the force field (both AMBER03 and YASARA2 were tested). Tables were generated for most optimal conformation. PRIME and YASARA also give output for likely dimerization. FoldX was utilized as a plugin within YASARA to achieve mutant comparisons. In summary, the FoldX algorithm may calculate protein-protein interactions, protein-DNA interactions, or mutations within a protein, whereby FoldX calculates ΔΔG of interaction: ΔΔGab = ΔGab−(ΔGa+ΔGb)+ ΔGkon+ΔSsc [83]. Here, ΔGkon reflects the effect of electrostatic interactions on the kon and ΔSsc is the loss of translational and rotational entropy upon making the complex.

### Molecular dynamics methods

Charmm27, Amber, and OPLS2005 force fields were tested with the current release of NAnoscale Molecular Dynamics 2 engine. The protein with hydrogens consists of 7,083 atoms. In all cases, we neutralized with counter-ions, and then created a solvent with 150 mM Na+ Cl- to recreate physiological strength. TIP3P water molecules were added around the protein at a depth of 15–18 Å from the edge of the molecule depending upon the side [84]. Our protocol has been previously described in the literature [36]. Solvated protein simulations consist of a box with between 1.17×10^5^ atoms including proteins, counter-ions, solvent ions, and solvent waters. Simulations were carried out using the particle mesh Ewald technique with repeating boundary conditions with a 9 Å nonbonded cut-off, using SHAKE with a 2-fs timestep. Pre-equilibration was started with 100,000 steps of minimization followed by 10000 ps of heating under MD, with the atomic positions of protein fixed. Then, two cycles of minimization (100000 steps each) and heating (2000 ps) were carried out with restraints of 10 and 5 kcal/(mol·Å^2^) applied to all protein atoms. Next, 50000-steps of minimization were performed with solute restraints reduced by 1 kcal/(mol·Å^2^). Then, 1000 ps of unrestrained MD were carried out, and the system was slowly heated from 1 to 310 K. The production MD runs were carried out with constant pressure boundary conditions (relaxation time of 1.0 ps). A constant temperature of 300 K was maintained using the Berendsen weak-coupling algorithm with a time constant of 1.0 ps. SHAKE constraints were applied to all hydrogens to eliminate X-H vibrations, which yielded a longer simulation time step (2 fs). Our methods for equilibration and production run protocols are in the literature [35], [81], [85], [86]. Equilibration was determined from a flattening of RMSD over time after an interval of>20 ns. Our biasing algorithm, MdMD (MdMD section) expedited conformational searching over timescales inaccessible otherwise. Translational and rotational center-of-mass motions were initially removed. Periodically, simulations were interrupted to have the center-of-mass removed again by a subtraction of velocities to account for the “flying ice-cube” effect [87]. Following the simulation, the individual frames were superposed back to the origin, to remove rotation and translation effects.

### Maxwell's demon molecular dynamics

The MdMD algorithm has been previously described in exhaustive detail for smaller systems [36]. The application of MdMD allows the user to alter the shape of X-ray crystallographic structures to match the cryogenic-electron microscopic (cryo-EM) data, which may present an alternative conformation of the structure. In doing so, the cryo-EM density can drive the MD toward an unknown conformation. By automating this process, human biases and errors are minimized for the model making process. To prevent local wells, the time of sampling, MD sprint varies and Boltzmann velocities can be applied directionally. MdMD algorithm was implemented to collect representative pathway data for Parkin dynamics between LC-MOD/MCMM generated states. Average time for MdMD pathway is between 10 and 20 ns, while the count for discarded states during global variable testing ranges from 16% to 8%. Several replicate runs of MdMD were performed.

### LC-MOD/MC and MCMM/LMCS conformation generation

Using Schrödinger's Conformational Search Suite, we employed the following scheme. Potential terms were set using the OPLS2005 force field with water solvent and charges designated from the force field. We used an extended cutoff for electrostatic calculations (default: Van der Waals 8.0 Å, Electrostatic 20.0 Å, H-bond 4.0 Å). We utilized constraints fixed on each zinc atom and the associated zinc-finger amino acid residues (i.e. cysteines, histidines, etc), which were determined using harmonic restraints at a force constant of 50 kcal/mol per selected atom. Additionally, torsions of the adjacent amino acid to the zinc-binding amino acid was softly constrained with a 10 kcal/mol force constant. Every other atom of the system was left to freely move under the conditions of the search algorithm, and the constrained pairs were assigned relative to each other, not to coordinate space, such that the zinc-finger could move as a single unit during conformational searches. The Powell-Reeves conjugate gradient energy minimization method was utilized on conformations achieved to return the structure to its local minimum for that particular conformation [88].

Several search schemes were applied to Parkin to look for large global conformational variation. Using the conformational search engine, we examined torsional sampling with MCMM, Mixed-torsional/Low-mode sampling, Large-scale Low-mode sampling, and Mixed torsional/Large-scale low-mode sampling (LC-MOD). When using the LLMOD search, we set the initial convergence criteria to 1.000. To customize the search we used enhanced torsion sampling options with distance and torsion checking. Maximum number of steps per attempt was 5,000 with 100 steps per rotatable bond, saving 10 structures per search. The default energy window for saving structures is 5.02 kcal/mol. Probability of a torsion rotation/molecule translation is 0.5. Minimum distance for a low-mode move is 3.0 Å and maximum is 7.5 Å. The search was continued for 100 s of conformers, retaining only lowest energy models and discarding unrealistic structures. Structures from the LCMOD search form guidepost structures for MdMD biasing of the initial model to traverse the landscape of structures generated using Schrödinger's Large-scale sampling using the initial crude guideposts and various biasing schemes. Final simulations relied on MdMD for smooth pathway transitions.

### Modification and mutation modeling

Mutations of amino acids were completed using the Maestro within the Schrödinger suite with the mutate residue feature. Additionally, the build panel in Maestro conveniently allows for placing mutated residues (or growing them) automatically within an existing peptide chain. Also, MacroModel features within Maestro allow for the quick minimization of the structure for local geometry fixes to correct stereochemistry and packing of the amino acids. Modifications for amino acids, such as phosphorylation of Serine-65 (pSer65), was achieved using the 2D sketcher and importing as an new amino acid type which can be parameterized using the Schrödinger force fields. Using OPLS2005 or YASARA2, one can parameterize these modification and import into existing molecular dynamics integration engines, as the parameters for the modification are well documented for YASARA and Schrödinger [42]–[44], [63], [89].

### MacroMoleculeBuilder

MMB is an internal coordinate mechanics (ICM) [38] code which models 3D the structure and dynamics of macromolecules. It allows the user to control the mobility of all bonds and add constraints and forces [90]. MDS treats all atoms as independent particles in Cartesian coordinates. In ICM all atoms in a molecule are connected to each other, mostly by pin joints, which allow dihedral torsions about the bond axis. However the user may choose to also allow bond stretching and angle bending, or no freedom at all. Specified residues may also be constrained to a fixed relative position and orientation, with respect to each other or to the ground frame. The Amber PARM99 [91] or other force field can be applied, and its bonded and non-bonded terms can be separately scaled by the user [90]. MMB has successfully been used in RNA folding from biochemical data [92], threading [93], [94], and flexible fitting to density maps [95].

### Zone equilibration of mutants

ZEMu is implemented in MMB [37], [96]. It consists of first, introducing the mutation and finding an energetically favorable local rearrangement around the mutation site, and second, computing the change in interaction energy (ΔΔG) using a knowledge-based (KB) potential. ZEMu equilibration of the mutation site proceeds as follows: First we specified a small (five residue) flexibility zone centered about the mutation site. The flexibility zone is treated in torsion space, leaving the remainder of the protein rigid and fixed. We then specified a larger, enclosing physics zone inside of which electrostatic and van der Waals forces are active. Due to the lack of solvent or other rigorous treatment of viscosity, it is possible for small chemical groups such as methyl to spin unnaturally quickly, which results in the variable time step integrator taking very small time steps. To deal with this we artificially scale the inertia matrices of such groups by a factor of 11.0 – empirically found [37] to be sufficient to lengthen time steps without significantly affecting results. This method establishes a flexibility zone and a (generally larger) physics zone in the protein [97], [98]. The physics zone includes all residues within 12 Å of the flexible residues. The flexibility zone includes the mutated residue plus at least two residues on each side, for a total of five residues, in order to model the backbone rearrangements induced by the mutation [37].

### Prediction of binding energy changes in proteins

For each proposed mutation locus, we equilibrated the corresponding flexibility zone in the wild type complex and evaluated the free energy of unfolding (ΔG_wt_) with the KB potential FoldX [99]. We repeated the calculation for the *mutant* complex to obtain the respective free energy of unfolding (ΔG_mutant_). The difference between these two quantities is our estimate of the experimental change in binding free energy induced by the mutation [100]: ΔΔG = ΔG_wt_−ΔG_mutant_. Using MMB and ZEMu with these equilibration and binding energy evaluation required an average of 15 minutes per protein, on a single core of a 3.00 GHz AMD Opteron 6220 processor. All other calculations in the above MD sections were completed on a Xeon-based cluster with 120 hexa-core processors with 256 GB RAM available.

### Cloning and mutagenesis

FLAG-Parkin and pEGFP-Myc-Parkin wild type have been described before [3]. Mutant Parkin was cloned using site-directed mutagenesis. Constructs were sequence verified using BigDye Terminator v.3.1 and an ABI 3100 Genetic Analyzer (Applied Biosystems). Primer sequences can be obtained upon request.

### Cell culture

Human epithelial cancer cells (HeLa) were obtained from the ATCC (American Type Culture Collection), human embryonic kidney cells (HEK293E) from Invitrogen. Cells were maintained in DMEM containing 10% FBS at 37°C under humidified conditions and 5% CO_2_.

### High content imaging

The Parkin mitochondrial translocation assay has been previously been described [39]. Cells were seeded with 4000 cells/well in 96-well imaging plates (BD Biosciences) and allowed to attach overnight. Cells were transfected with empty vector, GFP-Myc-Parkin wild type or mutants using Lipofectamine2000 (Invitrogen). 48 h after transfection cells were washed 1× in PBS and fixed for 20 min in 4% paraformaldehyde. Nuclei were stained with Hoechst 33342 (1: 5000, Invitrogen) for 10 min and cells washed twice in PBS. Plates were imaged on a BD Pathway 855 with a 20× objective using a 3×3 montage (no gaps) with laser autofocus every second frame. Raw images were processed using the build-in AttoVision V1.6 software. Regions of interest (ROIs) were defined as nucleus and cytoplasm using the build-in ‘RING - 2 outputs’ segmentation for the Hoechst channel after applying a shading algorithm. As a measure of Parkin translocation, the ratio of GFP signal intensity in the cytosol/nucleus was calculated. To exclude non-transfected cells and to ensure comparable transfection levels among analyzed cells, only ROIs with a GFP signal at least 30% higher than background were taken into consideration. Per Parkin construct we analyzed at least 4 independent experiments with 4 wells each per time point.

### Immunostaining

HeLa cells were plated onto glass coverslips coated with poly-D-lysine (Sigma), fixed with 4% paraformaldehyde and permeabilized with 1% Triton-X-100 in PBS. Cells were incubated with primary anti-TOM20 (1∶2000, ProteinTechGroup 11802-1-AP) and anti-p62 antibodies (1∶500, BD Biosciences 610832) followed by incubation with secondary antibodies anti-mouse IgG Alexa Fluor-647 and anti-rabbit Alexa Fluor-568 (Molecular Probes) diluted 1∶1000. Nuclei were stained with Hoechst 33342 (1∶5000). Coverslips were mounted onto slides using fluorescent mounting medium (Dako). Confocal fluorescent images were taken with an AxioObserver microscope equipped with an ApoTome Imaging System (Zeiss).

### Oxyester analysis

HeLa cells were transfected with FLAG-Parkin C431S variants using Lipofectamine 2000 according to manufacturer's protocol and medium was replaced 4 h later. The next day, cells were treated with 10 µM CCCP for 0, 1, 2, 4, or 16 h. Cells were harvested in preheated (95°C) SDS lysis buffer (50 mM Tris pH 7.6, 150 mM NaCl, 1%SDS). Lysates were homogenized by 10 strokes through a 23G needle. Protein concentration was determined by use of bicinchoninic acid (Pierce Biotechnology). To verify the band shift by oxyester formation, aliquots of lysates were treated with or without NaOH (100 mM final) for 1 h at 37°C. NaOH was neutralized by addition of equal amounts of HCl before samples were run on 8–16% Tris-Glycine gels and transferred onto polyvinylidene fluoride membranes (Millipore). Membranes were incubated with anti-FLAG antibody (1∶100,000, Sigma F3165) overnight at 4°C followed by HRP-conjugated anti-mouse secondary antibodies (1∶15,000; Jackson ImmunoResearch 115-035-003). Bands were visualized with ImmobilonWestern Chemiluminescent HRP Substrate (Millipore) using a LAS-3000 Imager (Fuji).

### Immunoprecipitation-coupled *in vitro* ubiquitination assay

HEK293E cells transfected with FLAG-Parkin wild type were incubated with or without 10 µM CCCP for 1 h prior to lysis. Cells were washed once in cold 1× HBS (20 mM HEPES pH 7.4, 150 mM NaCl) and lysed in IP buffer (50 mM HEPES pH 7.5, 10 mM KCl, 150 mM NaCl, 1 mM EDTA, 0.5 mM EGTA, 0.2% NP-40+Complete (Roche)). PhosStop (Roche) was added to all samples that were not treated with phosphatase. 750 µg protein was immunoprecipitated for 4 h using 20 µl of anti-FLAG EZview beads (Sigma). Beads were washed twice in cold IP buffer and once in 1× Ub buffer (20 mM HEPES pH 7.4, 50 mM NaCl, 5 mM MgCl_2_) before supernatant was completely removed. Phosphatase treated samples were washed 2× in IP buffer before supernatant was removed and 8 µl H2O, 2 µl 10× buffer and 10 U phosphatase (FastAP, Fermentas) were added to the beads for 30 min at 37°C. Beads were then washed twice with 1× Ub buffer before ubiquitination reaction. Recombinant proteins were purchased from Boston Biochem. Ubiquitination reaction contained 100 nM E1 enzyme (GST-Ube1, #E306), 1 µM E2 enzyme (#E2-622, #E2-640 or #E2-664), 2.5 mM ATP, 2 mM DTT, 5 µg untagged ubiquitin (#U-100H), 1 µg N-terminally biotinylated ubiquitin (#UB-560) in 1× Ub buffer in a 20 µl reaction. Ubiquitination reaction was carried out for 1.5 h at 37°C. Laemmli buffer was added and samples boiled for 10 min at 95°C. Samples were run on a 8–16% Tris-glycine gel, blotted and probed with horseradish peroxidase-coupled Streptavidin (1∶100,000; Jackson Immunoresearch 016-030-084) and anti-FLAG antibody (1∶100,000, Sigma A8592).

### Statistical analysis

Statistical analysis was performed with one-way ANOVA followed by Tukey's post-hoc test. Error bars indicate S.E.M.

## Supporting Information

Figure S1 **Comparison of the new Ser65 and pSer65 Parkin models.** Superposition overlay of both Ser65 and pSer65 models for human full-length Parkin structure. Ser65, pSer65 and Cys431 are shown in VdW, while the individual domains are in ribbons and colored-coded.
(TIF)

Figure S2 **Comparison between Parkin with and without pSer65.** RMSD comparison between human full-length pSer65 Parkin structural model and X-ray structures (4K7D, 4K95, 4BM9, 4I1H) for backbone residues 141–465, which measure to the initial conformation of pSer65 the following RMS values: 3.56 Å, 3.48 Å, 2.92 Å, and 1.72 Å, respectively.
(TIF)

Figure S3 **Zoom into Parkin crystal structure (4K95).** Region surrounding Ser65 in the UBL domain is shown. Residues Trp45, Leu61, Val67 and Ser65 are highlighted. Ser65 establishes a hydrogen bond with the main chain of Asp62, while this interaction is lost in all mutants. Though S65D and S65E introduce a negative charge that should perturb the electron distribution in the adjacent hydrophobic pocket and decrease the stability of the UBL domain, we obtained ΔΔG values lower than expected from structure gazing (ΔΔG = 0.6 kcal.mol^−1^ and 1.2 kcal.mol^−1^, respectively). On the other hand, S65A should further stabilize the UBL domain by expanding the hydrophobic pocket electron distribution resulting in a ΔΔG of −0.7 kcal.mol^−1^. The RMS error associated with the method is 1.53 kcal/mol for all mutants (1245 mutants of 65 co-crystals) and 1.15 kcal/mol for the stabilizing mutants [37].
(TIF)

Figure S4 **Mutations of Ser65 delay Parkin translocation, p62 recruitment and mitochondrial clustering.** **A**) Analyzed GFP-Parkin constructs had similar expression levels. Shown is the average signal intensity of the cytoplasmic plus the nuclear GFP signal at 0 h CCCP treatment across all analyzed wells after nontransfected, GFP-negative or low level expressing cells were excluded. GFP alone showed a higher expression levels than the GFP-Parkin fusion proteins (n>16 wells, one-way ANOVA, Tukey's post-hoc, p<0.0001, F = 27.24, ns – not significant, *** p<0.0005). **B–D**) Shown are representative immunofluorescence images before and after 2 h or 4 h CCCP treatment. Individual and merged channels are given: GFP-Parkin in green, TOM20 (mitochondria) in red, Ub adaptor protein p62 in cyan and nuclei (Hoechst) in blue. Scale bars correspond to 10 µM.
(TIF)

Figure S5 **Ramanchandran plot for the average structure from MDS indicating normal distribution of Phi-Psi space for protein geometry.** Additional measurements using What-If within Yasara were completed for backbone measurements, 1D/2D packing, and dihedrals/rotamers (see Methods). Both α-helical and β-sheet quadrants on the left side are well populated, random coil is shown on the right.
(TIF)

Figure S6 **Linearity of MdMD algorithm at driving Parkin from state 1 to state 5.** MdMD algorithm is applied following 10 ns production (post-equilibration) MD to allow the structure(s) to relax. After MdMD algorithm starts, the RMS rapidly and linearly descends toward State 5 with final RMSDs <3 Å for all replicates. All RMS calculations are relative to the final state (State 5) from generated models described in methods.
(TIF)

Figure S7 **Stability of the C-terminal region of Parkin (146–465) protein during MdMDS and MDS simulations.** RMSD for protein backbone of the four replicates stays between 2 and 4.5 Å. RMSD was calculated relative to the final state from MdMD with the replicate in brown indicated the reference structure (RMSD goes to zero at end).
(TIF)

Figure S8 **RMS fluctuation of Parkin amino acid residues during free MDS versus MdMD simulation.** The fluctuation of amino acids from UBL versus residues 141–465 is shown with the MdMD line in black and the free MDS line in red. Residues 141–465 (black) stay within 4–6 Å of initial structural model. Apparently the zinc fingers greatly stabilize those regions of the structure. The UBL domain and residues through 140 show greatest fluctuation during MdMD, which traverses from IBR to C431. For the free MDS trajectory, the overall structure is stays under 7–8 Å for RMSF (red line). The red line is for Ser65 simulation and black line is for pSer65 simulation.
(TIF)

Figure S9 **RMSD of UBL-linker versus residues 141–465 from four replicates.** Residues 141–465 (blue) are <5 Å of initial structure for duration. Only local conformational fluctuations occur in the main body, which is rigidified by the presence of eight Zinc-fingers. The MdMD algorithm moves each replicate toward State 5 giving rise to large RMS changes that are shown in red, green and beige. For each replicate, the movement is almost similar giving closely spaced RMS changes during the common MD sprint interval.
(TIF)

Figure S10 **Parkin crystal structure close-up of the active site region.** The active site residue for Parkin (Cys431) is given with its neighboring residues that are implicated in coordinating the thioester linkage with Ub. In this inactive state, Cys431 is not making any contacts. The C431S mutation does not result in any significant structural changes.
(TIF)

Movie S1 **The improved human Parkin model.** A model for human full-length Parkin is shown with legend key for domain residues in lower left corner. Initial closed, auto-inhibited state for Parkin is shown. As animation progresses the surface of the protein is shown semi-transparent to account for the solvent-accessible surface area of Parkin. Surfaces are colored to match domains as indicated in Figure 1. Parkin then rotates 360° in the X-axis followed by Y-axis. Following the surface rendered pose, the partial open state is shown for a pSer65 activated Parkin model. The UBL-linker regions are adjusted away from IBR and REP region, prior to large movement across body of protein.
(MP4)

Movie S2 **Ser65 Parkin from free MDS for closed conformation (100 ns).** Representative animation for molecular dynamics of unmodified human full-length Parkin (Ser65) model is shown. Domains are colored as indicated in Figure 1.
(MP4)

Movie S3 **pSer65 Parkin from free MDS for closed conformation (200 ns).** Representative animation for molecular dynamics of modified human full-length Parkin (pSer65) model is shown. Domains are colored as indicated in Figure 1.
(MP4)

Movie S4 **pSer65 cleft opening with MDS starting in closed conformation.** Close-up view of the cleft pocket, which contains residues Met1, Ile2, Phe4, Arg6, Gln63, Gln64, Arg97, Pro113, and Ser116 in close proximity to pSer65 during the MDS run. Ribbons are colored by secondary structure and surface is shown to illustrate widening of gap between UBL and linker during simulation.
(MP4)

Movie S5 **MdMD pathway from a representative excursion of the UBL-linker domain across Parkin towards the active site.** A representative pathway via MdMD excursions between the initial MDS state 1 to the active site region in MDS state 5 is shown (see Figure 4). The Parkin molecule is colored as in Figure 1. Zn^2+^ atoms (spheres) as well as the coordinating Cys/His residues are shown.
(MP4)

Movie S6 **MdMD replicates of Parkin's opening.** Replicates of MdMD runs show the same trajectory as in Figure 4 and Movie S5. Zn^2+^ atoms are shown as red spheres.
(MP4)

Movie S7 **Successful docking of the E2∼Ub complex onto Parkin with unbiased MDS.** The E2 enzyme is shown in dark green, the Ub moiety in brown.
(MPG)

Movie S8 **Poor docking of the E2∼Ub complex onto Parkin with unbiased MDS.** The E2 enzyme is shown in dark green, the Ub moiety in brown.
(MPG)

Text S1 ***In silico*** **modeling of human Parkin.** Part 1: The modeling of individual fragments of Parkin is provided. Part 2: The consensus model for all fragments combined into complete full-length Parkin structure is provided.
(DOCX)
