# Supplementary figures and images for: Phosphorylation by PINK1 Releases the UBL Domain and Initializes the Conformational Opening of the E3 Ubiquitin Ligase Parkin
Source: PLoS Comput Biol. 2014 Nov 6;10(11):e1003935. doi: 10.1371/journal.pcbi.1003935 (PMC4222639; doi:10.1371/journal.pcbi.1003935)

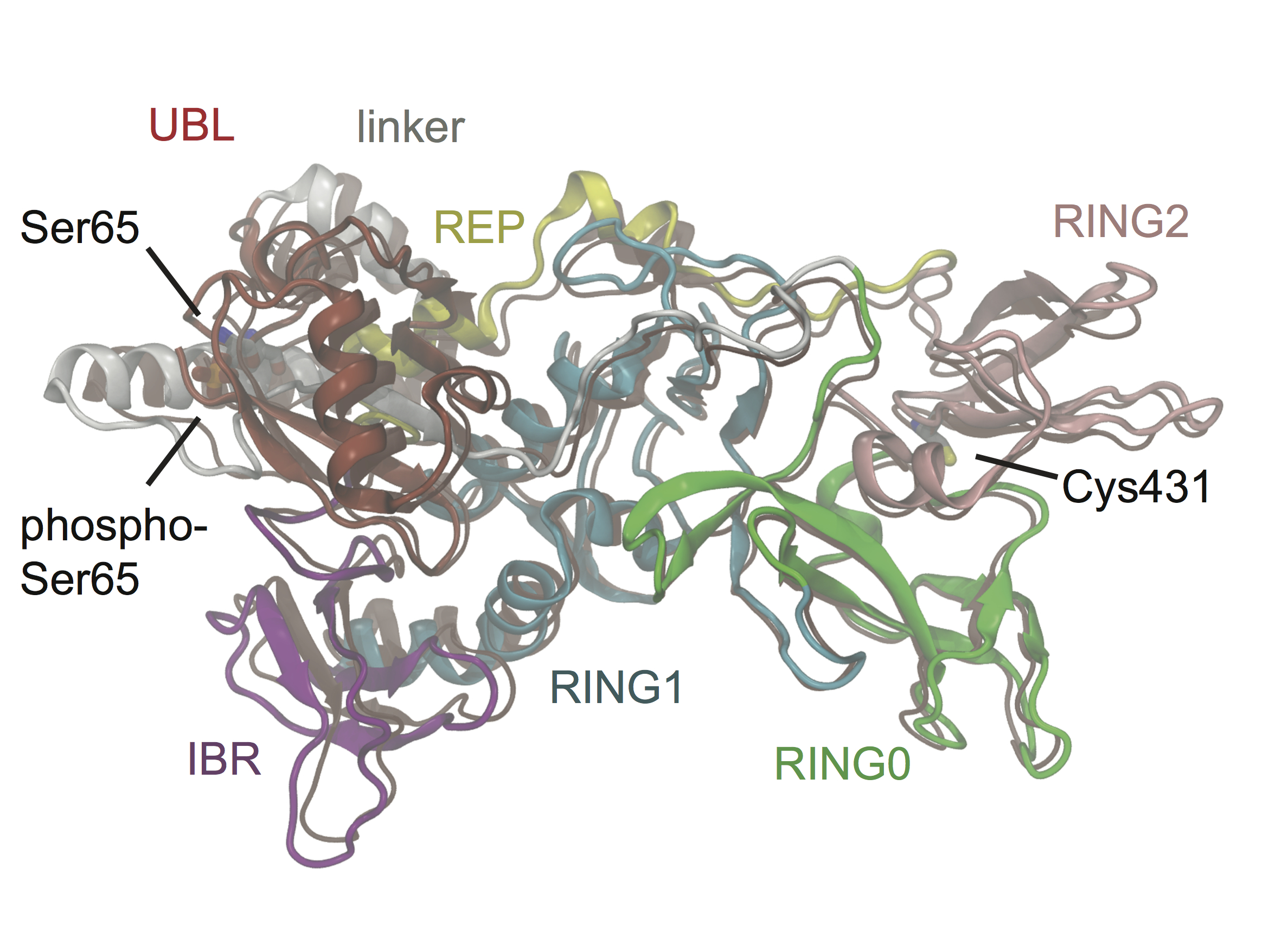

Supplement: Figure S1 — Comparison of the new Ser65 and pSer65 Parkin models. Superposition overlay of both Ser65 and pSer65 models for human full-length Parkin structure. Ser65, pSer65 and Cys431 are shown in VdW, while the individual domains are in ribbons and colored-coded. (TIF) [file pcbi.1003935.s001.tif]

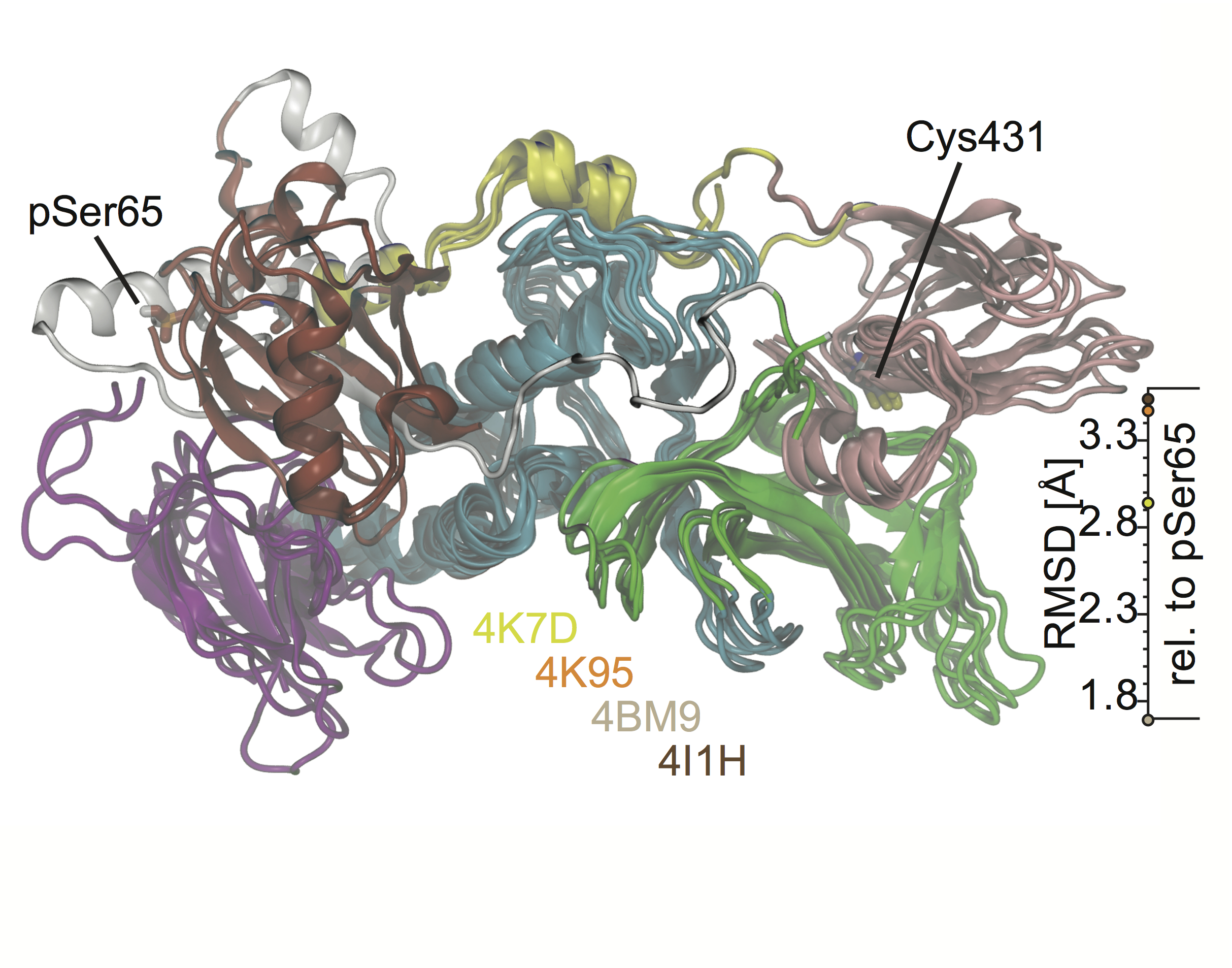

Supplement: Figure S2 — Comparison between Parkin with and without pSer65. RMSD comparison between human full-length pSer65 Parkin structural model and X-ray structures (4K7D, 4K95, 4BM9, 4I1H) for backbone residues 141–465, which measure to the initial conformation of pSer65 the following RMS values: 3.56 Å, 3.48 Å, 2.92 Å, and 1.72 Å, respectively. (TIF) [file pcbi.1003935.s002.tif]

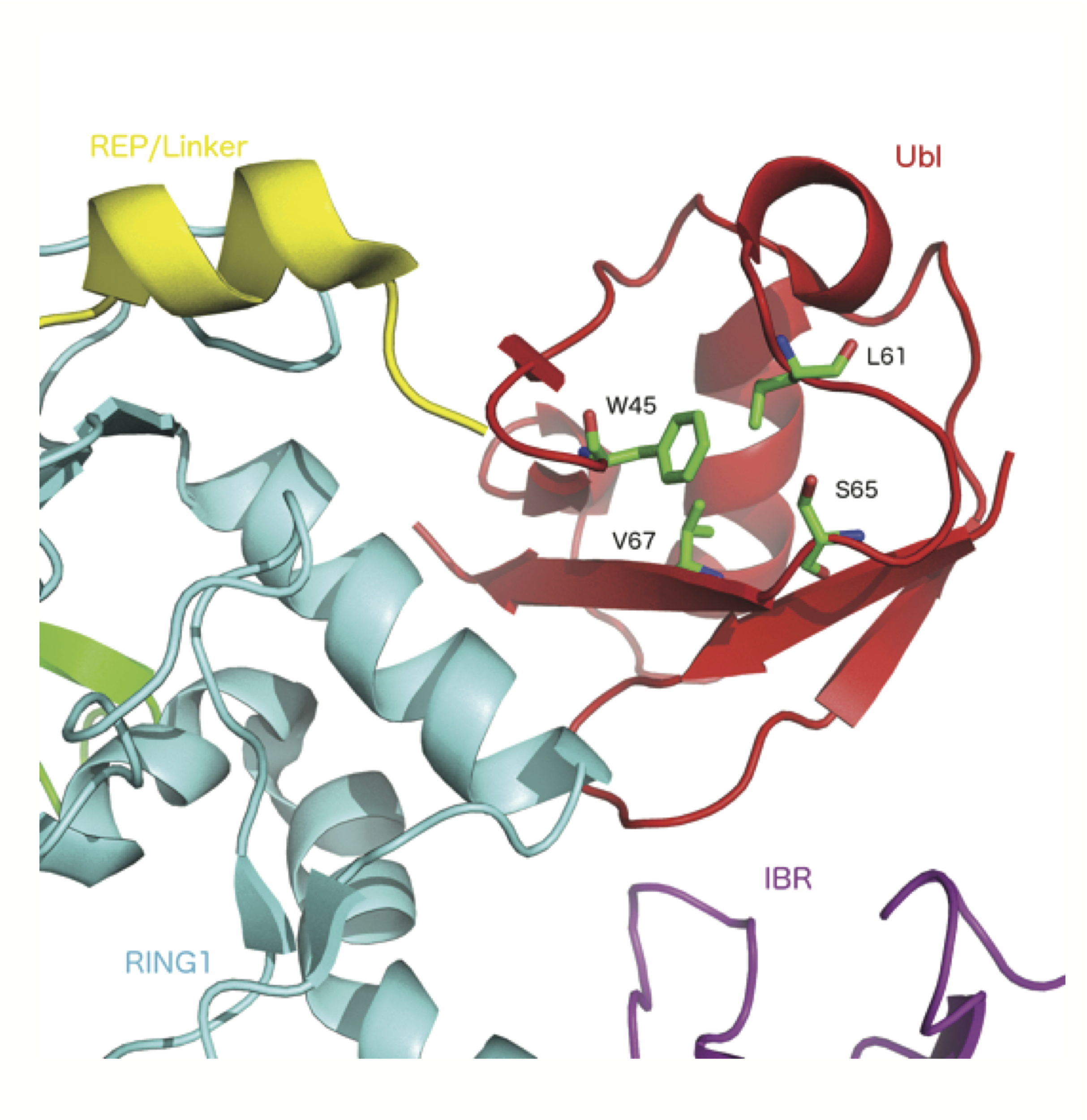

Supplement: Figure S3 — Zoom into Parkin crystal structure (4K95). Region surrounding Ser65 in the UBL domain is shown. Residues Trp45, Leu61, Val67 and Ser65 are highlighted. Ser65 establishes a hydrogen bond with the main chain of Asp62, while this interaction is lost in all mutants. Though S65D and S65E introduce a negative charge that should perturb the electron distribution in the adjacent hydrophobic pocket and decrease the stability of the UBL domain, we obtained ΔΔG values lower than expected from structure gazing (ΔΔG = 0.6 kcal.mol−1 and 1.2 kcal.mol−1, respectively). On the other hand, S65A should further stabilize the UBL domain by expanding the hydrophobic pocket electron distribution resulting in a ΔΔG of −0.7 kcal.mol−1. The RMS error associated with the method is 1.53 kcal/mol for all mutants (1245 mutants of 65 co-crystals) and 1.15 kcal/mol for the stabilizing mutants [37]. (TIF) [file pcbi.1003935.s003.tif]

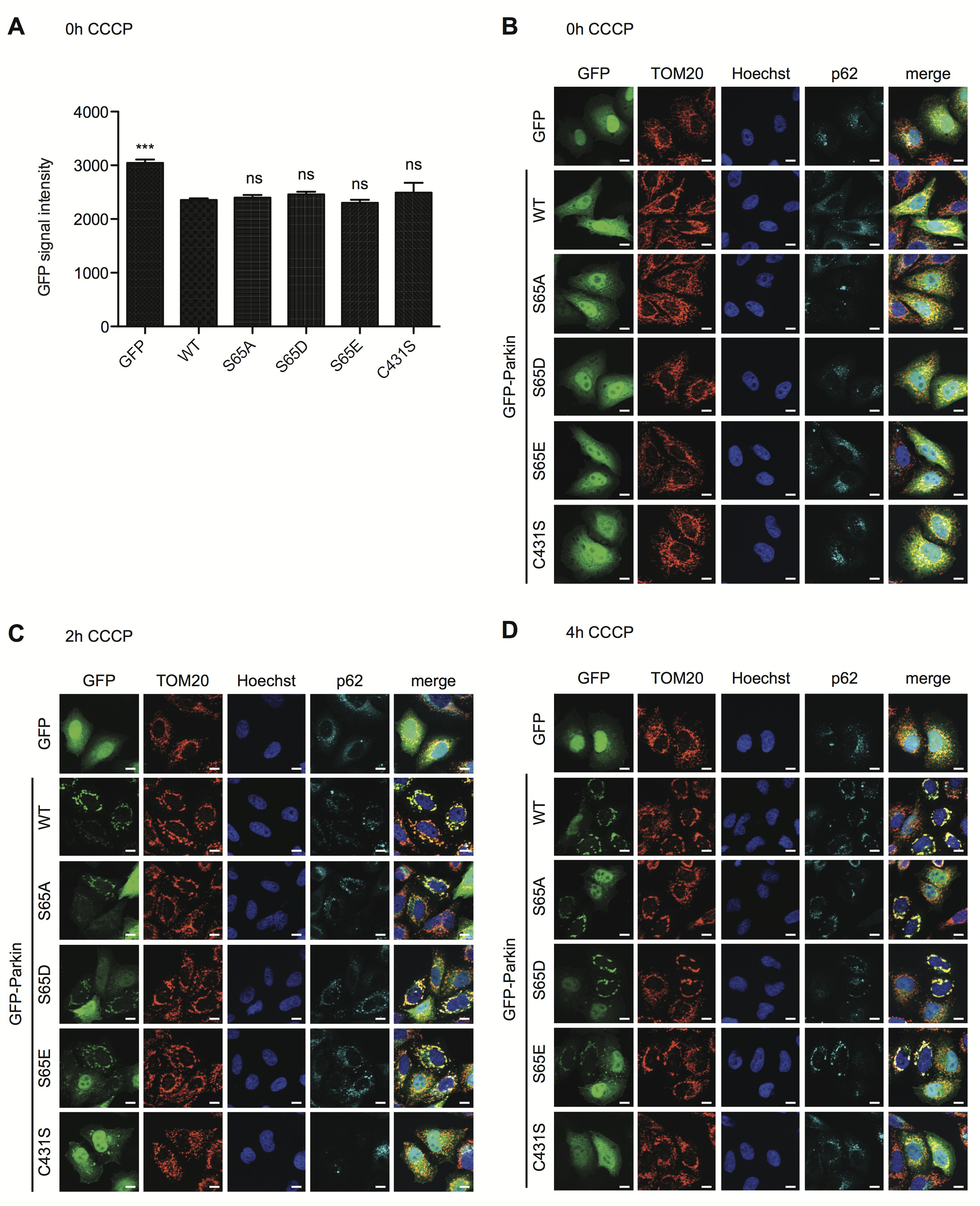

Supplement: Figure S4 — Mutations of Ser65 delay Parkin translocation, p62 recruitment and mitochondrial clustering. A) Analyzed GFP-Parkin constructs had similar expression levels. Shown is the average signal intensity of the cytoplasmic plus the nuclear GFP signal at 0 h CCCP treatment across all analyzed wells after nontransfected, GFP-negative or low level expressing cells were excluded. GFP alone showed a higher expression levels than the GFP-Parkin fusion proteins (n>16 wells, one-way ANOVA, Tukey's post-hoc, p<0.0001, F = 27.24, ns – not significant, *** p<0.0005). B–D) Shown are representative immunofluorescence images before and after 2 h or 4 h CCCP treatment. Individual and merged channels are given: GFP-Parkin in green, TOM20 (mitochondria) in red, Ub adaptor protein p62 in cyan and nuclei (Hoechst) in blue. Scale bars correspond to 10 µM. (TIF) [file pcbi.1003935.s004.tif]

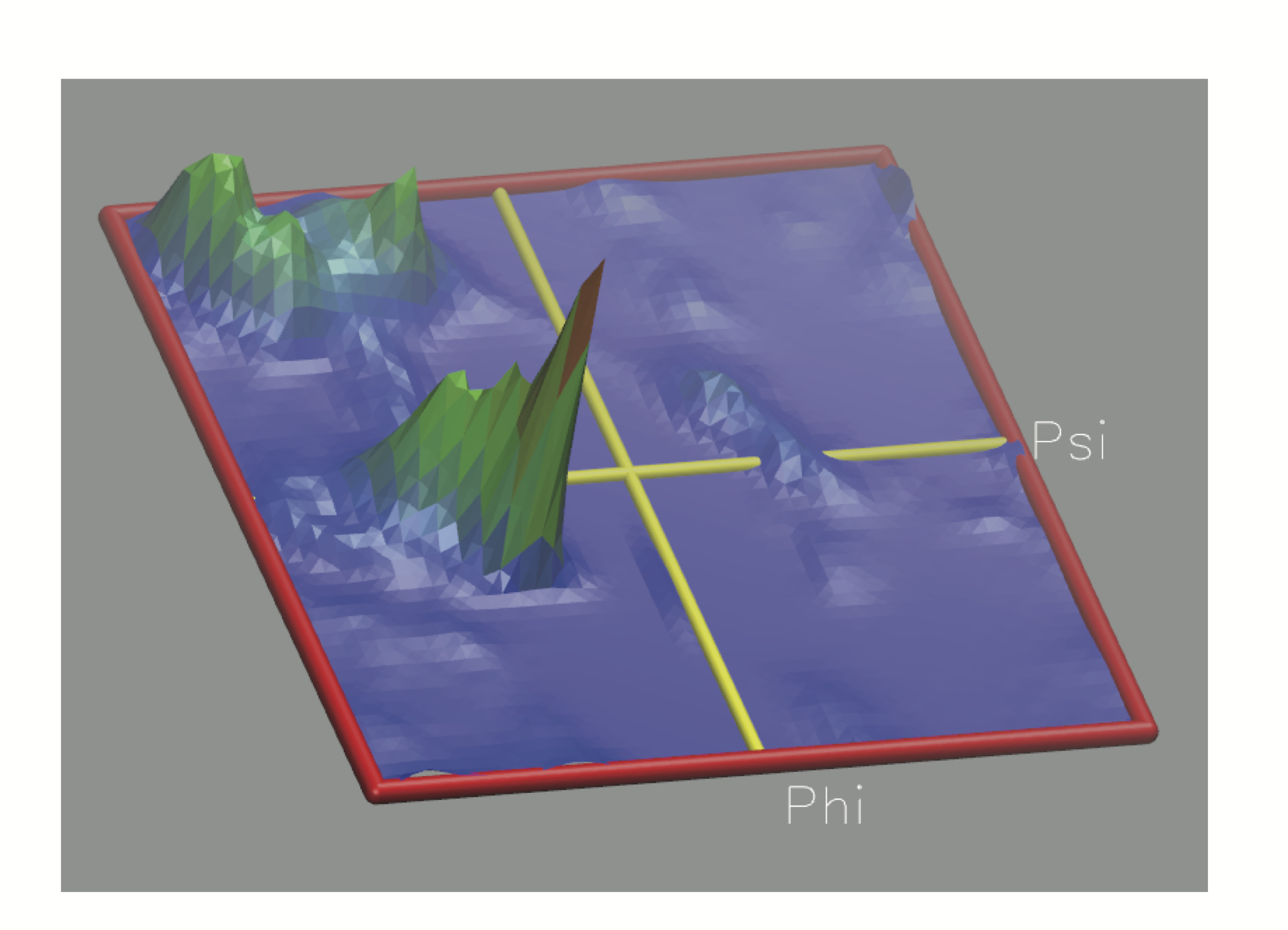

Supplement: Figure S5 — Ramanchandran plot for the average structure from MDS indicating normal distribution of Phi-Psi space for protein geometry. Additional measurements using What-If within Yasara were completed for backbone measurements, 1D/2D packing, and dihedrals/rotamers (see Methods). Both α-helical and β-sheet quadrants on the left side are well populated, random coil is shown on the right. (TIF) [file pcbi.1003935.s005.tif]

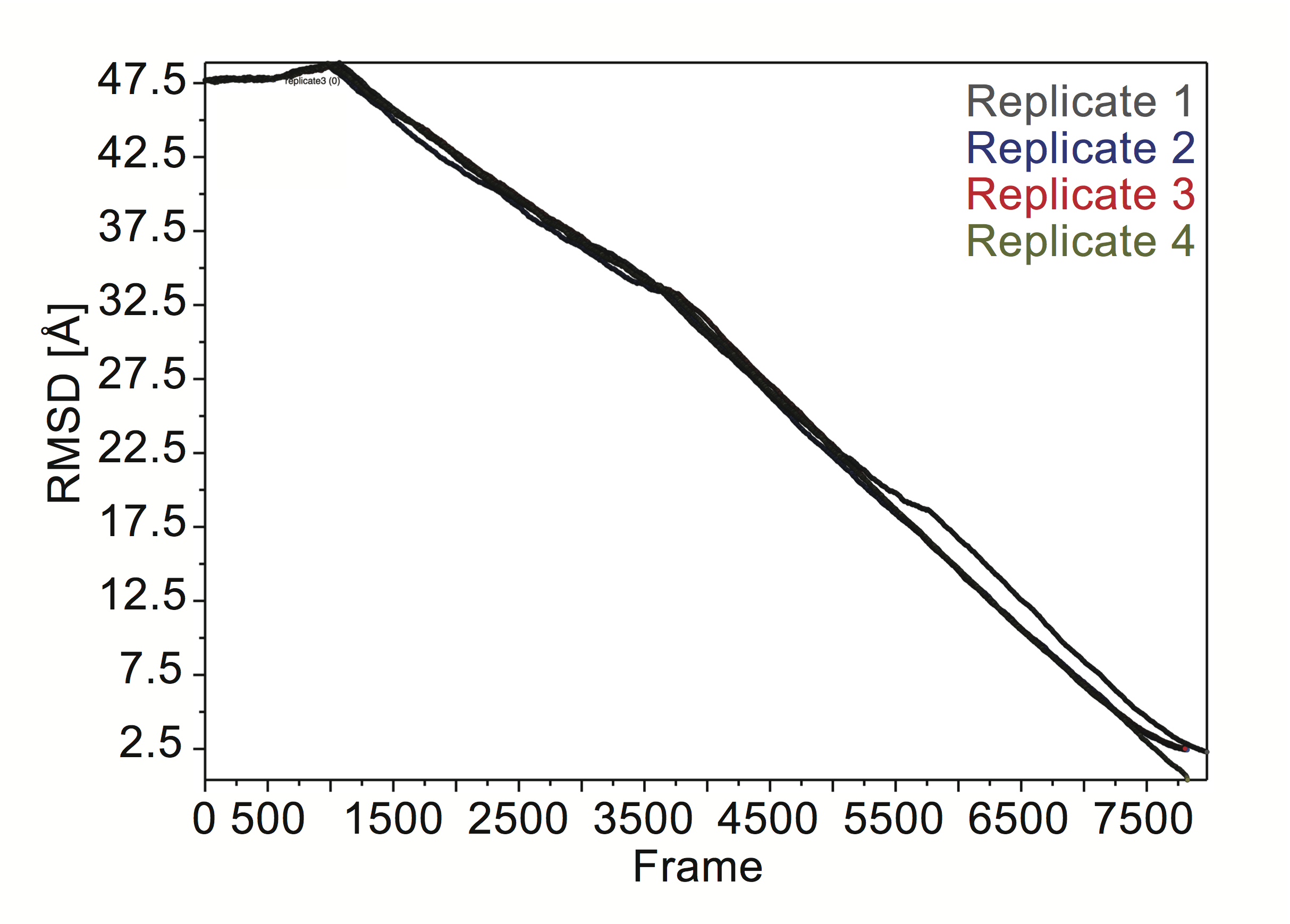

Supplement: Figure S6 — Linearity of MdMD algorithm at driving Parkin from state 1 to state 5. MdMD algorithm is applied following 10 ns production (post-equilibration) MD to allow the structure(s) to relax. After MdMD algorithm starts, the RMS rapidly and linearly descends toward State 5 with final RMSDs <3 Å for all replicates. All RMS calculations are relative to the final state (State 5) from generated models described in methods. (TIF) [file pcbi.1003935.s006.tif]

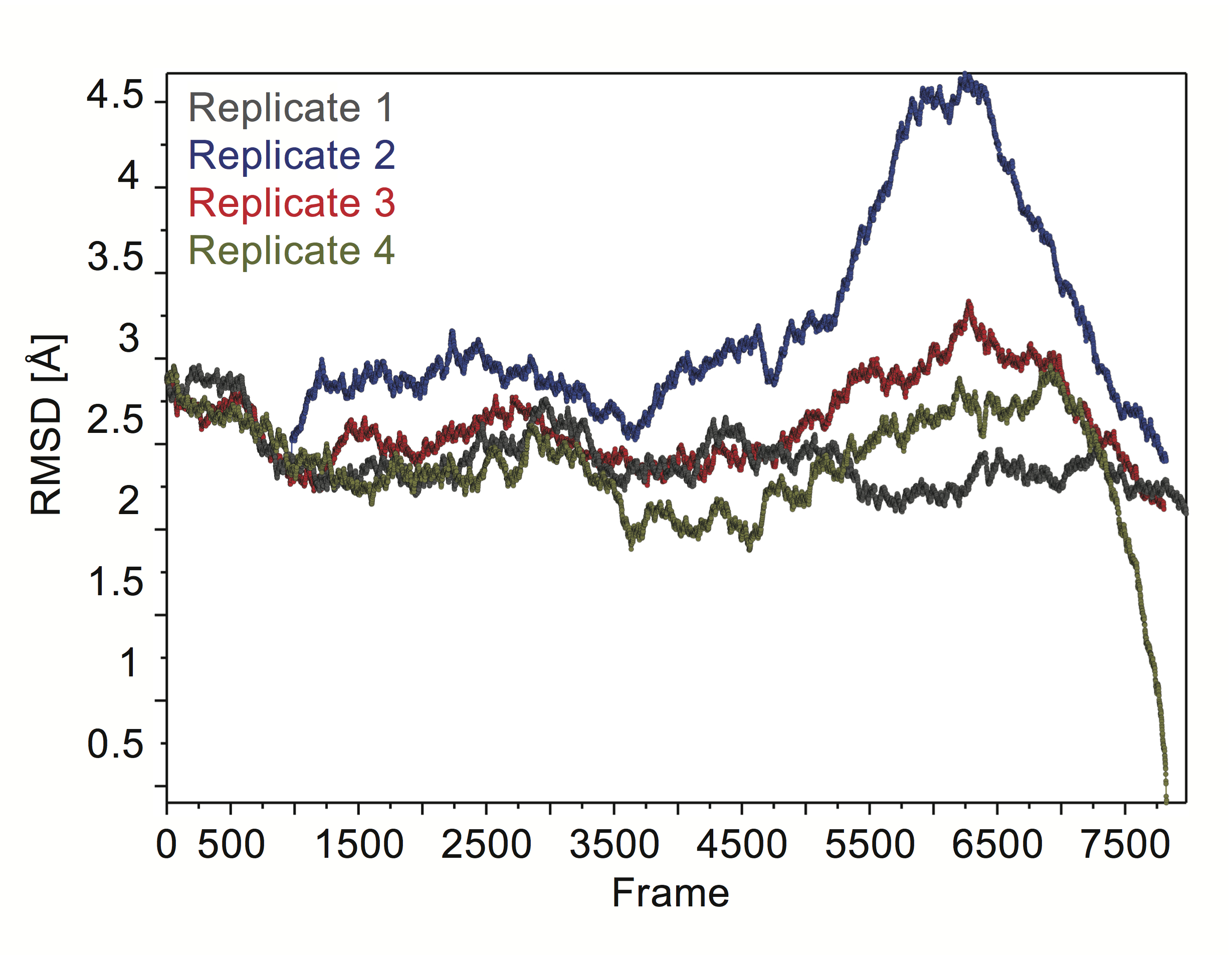

Supplement: Figure S7 — Stability of the C-terminal region of Parkin (146–465) protein during MdMDS and MDS simulations. RMSD for protein backbone of the four replicates stays between 2 and 4.5 Å. RMSD was calculated relative to the final state from MdMD with the replicate in brown indicated the reference structure (RMSD goes to zero at end). (TIF) [file pcbi.1003935.s007.tif]

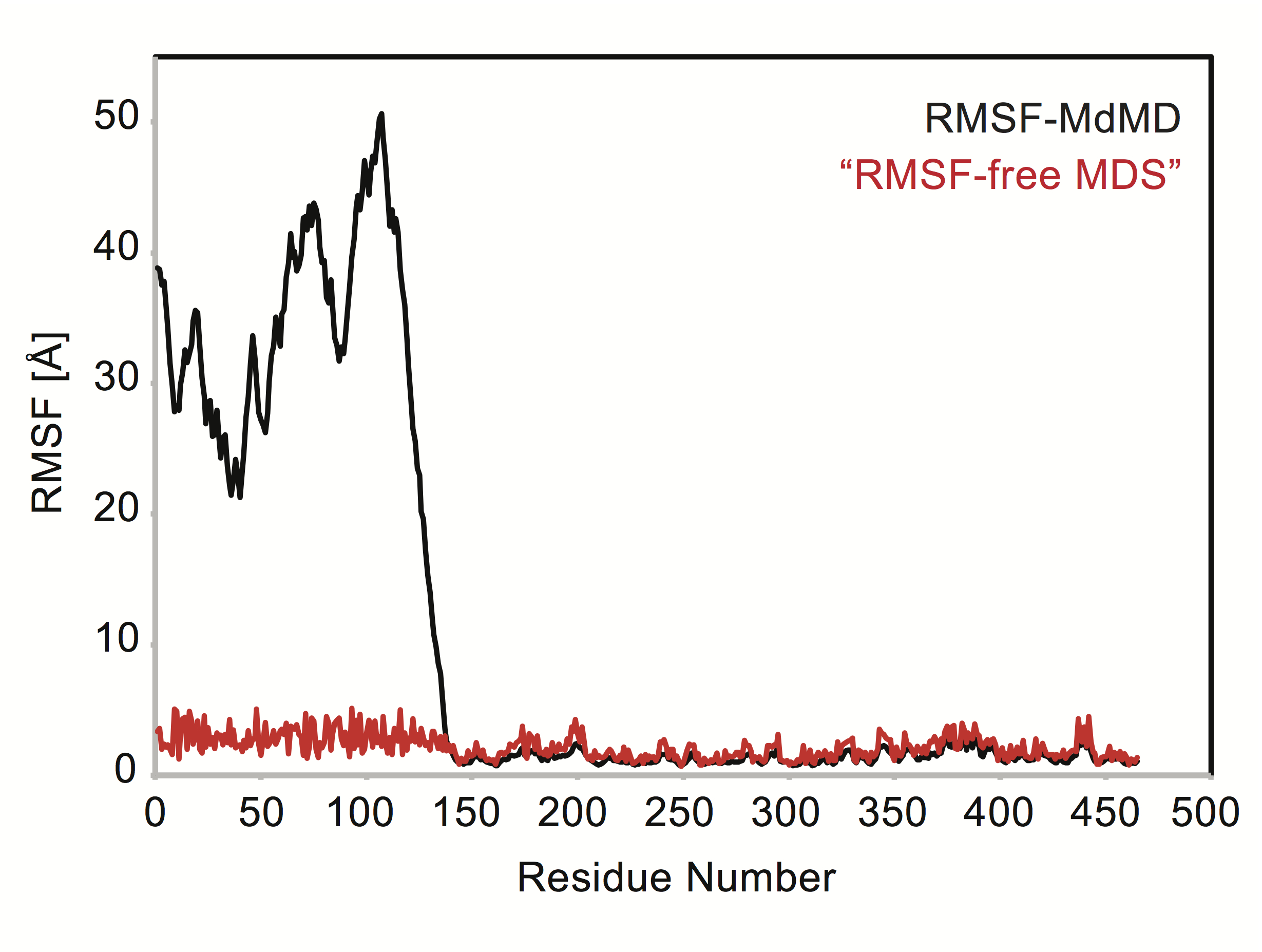

Supplement: Figure S8 — RMS fluctuation of Parkin amino acid residues during free MDS versus MdMD simulation. The fluctuation of amino acids from UBL versus residues 141–465 is shown with the MdMD line in black and the free MDS line in red. Residues 141–465 (black) stay within 4–6 Å of initial structural model. Apparently the zinc fingers greatly stabilize those regions of the structure. The UBL domain and residues through 140 show greatest fluctuation during MdMD, which traverses from IBR to C431. For the free MDS trajectory, the overall structure is stays under 7–8 Å for RMSF (red line). The red line is for Ser65 simulation and black line is for pSer65 simulation. (TIF) [file pcbi.1003935.s008.tif]

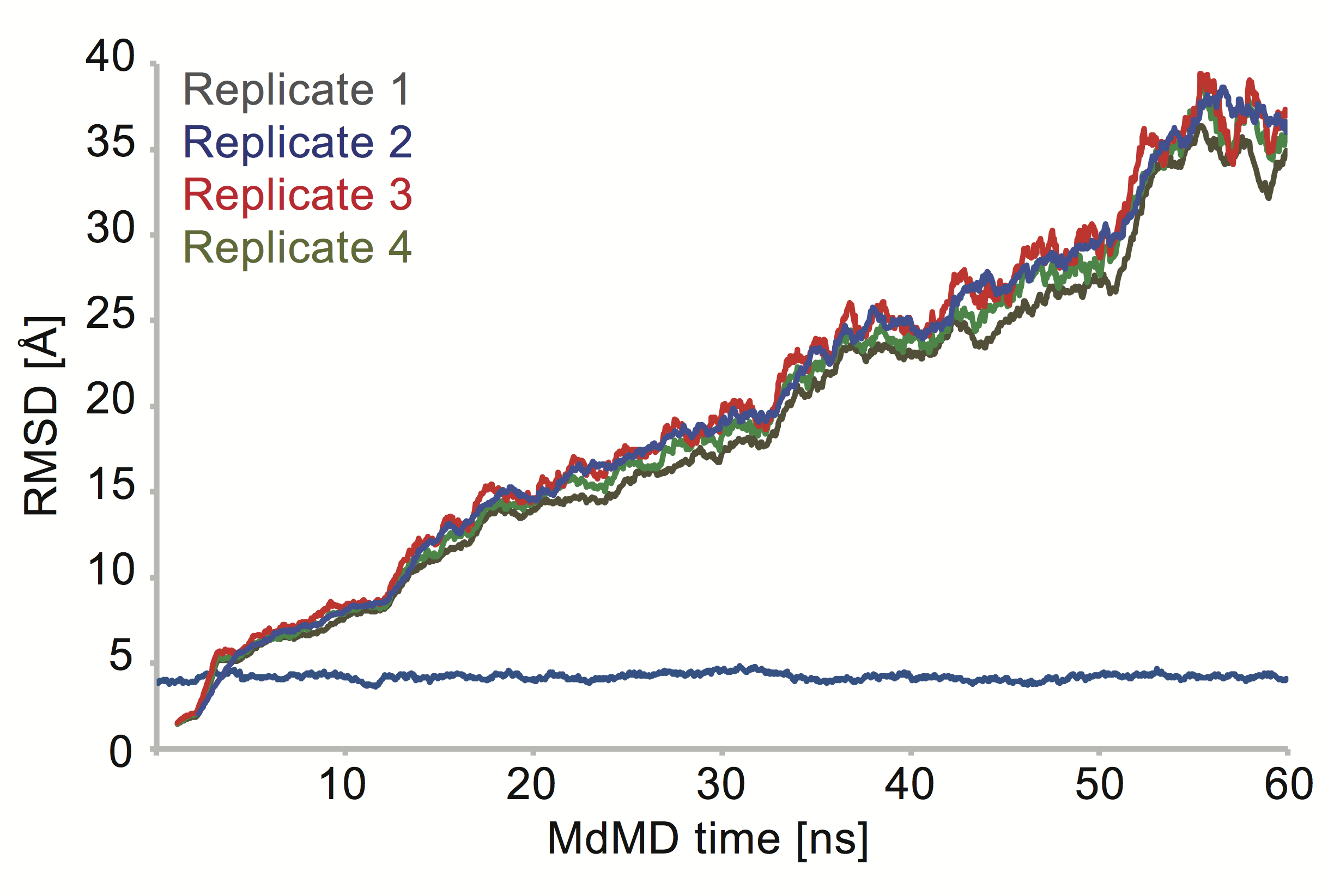

Supplement: Figure S9 — RMSD of UBL-linker versus residues 141–465 from four replicates. Residues 141–465 (blue) are <5 Å of initial structure for duration. Only local conformational fluctuations occur in the main body, which is rigidified by the presence of eight Zinc-fingers. The MdMD algorithm moves each replicate toward State 5 giving rise to large RMS changes that are shown in red, green and beige. For each replicate, the movement is almost similar giving closely spaced RMS changes during the common MD sprint interval. (TIF) [file pcbi.1003935.s009.tif]

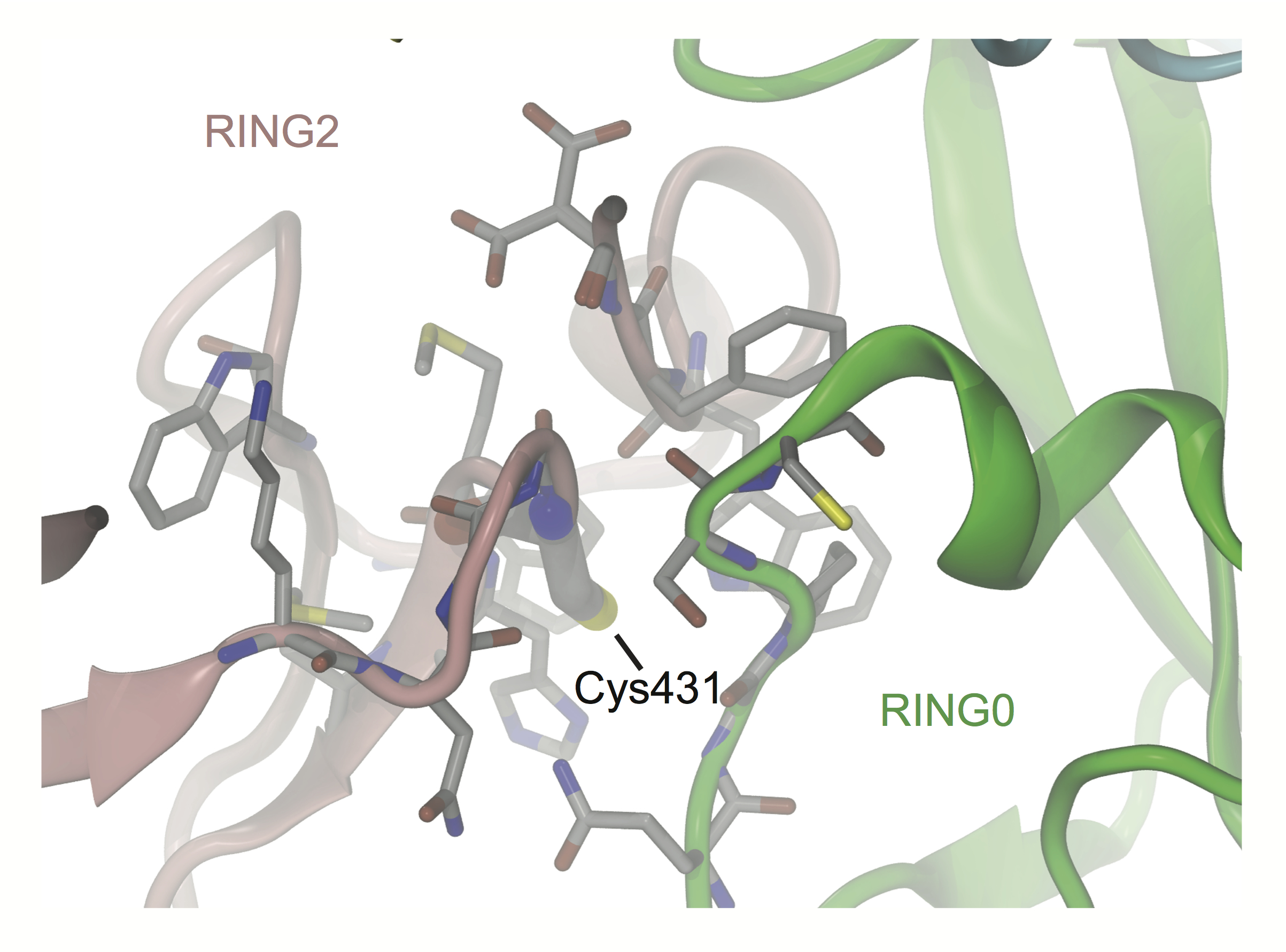

Supplement: Figure S10 — Parkin crystal structure close-up of the active site region. The active site residue for Parkin (Cys431) is given with its neighboring residues that are implicated in coordinating the thioester linkage with Ub. In this inactive state, Cys431 is not making any contacts. The C431S mutation does not result in any significant structural changes. (TIF) [file pcbi.1003935.s010.tif]
