# Supplementary material for: Phosphorylation by PINK1 Releases the UBL Domain and Initializes the Conformational Opening of the E3 Ubiquitin Ligase Parkin
Source: PLoS Comput Biol. 2014 Nov 6;10(11):e1003935. doi: 10.1371/journal.pcbi.1003935 (PMC4222639; doi:10.1371/journal.pcbi.1003935)
Supplement: Text S1 — In silico modeling of human Parkin. Part 1: The modeling of individual fragments of Parkin is provided. Part 2: The consensus model for all fragments combined into complete full-length Parkin structure is provided. (DOCX) [file pcbi.1003935.s019.docx]

**Text S1. *In silico* modeling of human Parkin.**

**Part 1: Pre-fragment modeling.**

**Structural Modeling Report for HPARKIN__TRC2013 1. The modeling target HPARKIN__TRC2013**

The three-dimensional structure of the following target sequence has been predicted by YASARA's homology modeling experiment:

>HPARKIN__TRC2013 MIVFVRFNSSHGFPVEVDSDTSIFQLKEVVAKRQGVPADQLRVIFAGKELRNDWTVQNCD LDQQSIVHIVQRPWRKGQEMNATGGDDPRNAAGGCEREPQSLTRVDLSSSVLPGDSVGLAVILHTDSRKDSPPAGSPAGRSIYNSFYVYCKGPCQRVQPGKLRVQCSTCRQATLTLTQGPSCWDDVLIPNRMSGECQSPHCPGTSAEFFFKCGAHPTSDKETSVALHLIATNSRNITCITCTDVRSPVLVFQCNSRHVICLDCFHLYCVTRLNDRQFVHDPQLGYSLPCVAGCPNSLIKELHHFRILGEEQYNRYQQYGAEECVLQMGGVLCPRPGCGAGLLPEPDQRKVTCEGGNGLGCGFAFCRECKEAYHEGECSAVFEASGTTTQAYRVDERAAEQARWEAASKETIKKTTKPCPR CHVPVEKNGGCMHMKCPQPQCRLEWCWNCGCEWNRVCMGDHWFDV

The target sequence contains 465 residues in 1 molecule.

**2. The modeling parameters**

The following parameters have been chosen for this target:

Modeling speed (slow = best): **Slow** Number of PSI-BLAST iterations in template search (PsiBLASTs): **3** Maximum allowed (PSI-)BLAST E-value to consider template (EValue Max): **3** Maximum number of templates to be used (Templates Total): **15** Maximum number of templates with same sequence (Templates SameSeq): **3** Maximum oligomerization state (OligoState): **4** (tetrameric) Maximum number of alignment variations per template: (Alignments): **15** Maximum number of conformations tried per loop (LoopSamples): **150** Maximum number of residues added to the termini (TermExtension): **15**

**3. The homology modeling templates**

Since the target sequence was the only available information, possible templates were identified by running 3 PSI-BLAST iterations to extract a position specific scoring matrix (PSSM) from UniRef90, and then searching the PDB for a match (i.e. hits with an E-value below the homology modeling cutoff 3.0).

The following 210 hits were found:

Total Template score

1 534.80 2 425.47

3 408.23 4 390.08 5 371.30

5 31.75

5 27.65

BLAST E- value

2e-56 3e-54

5e-55 4e-52 1e-54

1e-32

1e-14

Align score Cover

1837.0 81% 1715.0 66%

1728.0 66% 1688.0 65% 1604.0 67%

186.0 52%

333.0 16%

ID

4K95-L 4I1F-A

4I1H-A

4BM9- A

4K7D- C

4KBL-A

3B1L-X 4KC9-

Resolution

6.50 A 1.58 A

2.00 A 2.25 A 2.80 A

-

-

Header

Crystal Structure Of Parkin (375 residues with quality score 0.361)E3 UBIQUITIN-PROTEIN LIGASE PARKIN

Structure Of Parkin-s223p E3 Ligase (306 residues with quality score 0.377)**NOTE:** This template has been downloaded from PDB_REDO (www.cmbi.ru.nl/pdb_redo), since re-refinement improved the structure quality Z-score by 0.068.

Structure Of Parkin E3 Ligase (306 residues with quality score 0.359)**NOTE:** This template has been downloaded from PDB_REDO (www.cmbi.ru.nl/pdb_redo), since re-refinement improved the structure quality Z-score by 0.109.

Structure Of The Autoinhibited Parkin Catalytic Domain (301 residues with quality score 0.357)**NOTE:** This template has been downloaded from PDB_REDO (www.cmbi.ru.nl/pdb_redo), since re-refinement improved the structure quality Z-score by 0.216.

Crystal Structure Of Parkin C-terminal Ring Domains (304 residues with quality score 0.345)E3 UBIQUITIN-PROTEIN LIGASE PARKIN

Structure Of Hhari, A Ring-ibr-ring Ubiquitin Ligase: Autoinhibition Of An Ariadne-family E3 And Insights Into Ligation Mechanism (395 residues with quality score 0.328). **NOTE: This template was deliberately discarded:** Its total score 31.75 is lower than 30% of the best scoring X-ray template (534.80), and it newly covers only 0 target residues. It is therefore highly unlikely that this template would yield a useful model.

Crystal Structure Of Parkin Ubiquitin-Like Domain R33q Mutant (76 residues with quality score 0.508). **NOTE: This template was deliberately discarded:** Its total score 27.65 is lower than 30% of the best scoring X-ray template (534.80), and it newly covers only 3 target residues. It is therefore highly unlikely that this template would yield a useful model.

Structure Of Hhari, A Ring-ibr-ring Ubiquitin Ligase: Autoinhibition Of An Ariadne-family E3 And Insights Into Ligation Mechanism (414 residues with quality score 0.324). **NOTE: This template was deliberately discarded:** Its total score 27.37 is lower than 30% of the best

5 27.37

8e-33

163.0

337.0 115.0 115.0

113.0

113.0 115.0 115.0 115.0 115.0 115.0 116.0

113.0

119.0 116.0 116.0 110.0 114.0 113.0 110.0 113.0 124.0

52% 4KC9- - residues with quality score 0.324). **NOTE: This template was deliberately discarded:** Its total score 27.37 is lower than 30% of the best A scoring X-ray template (534.80), and it newly covers only 0 target residues. It is therefore highly unlikely that this template would yield a

useful model. 2ZEQ- Crystal Structure Of Ubiquitin-Like Domain Of Murine Parkin (78 residues with quality score 0.485). **NOTE: This template was**

16% A - **deliberately discarded:** Its total score 26.71 is lower than 30% of the best scoring X-ray template (534.80), and it newly covers only 0 target residues. It is therefore highly unlikely that this template would yield a useful model.

Crystal Structure Of The Mouse Hoil1-l-nzf In Complex With Linear Di- Ubiquitin (150 residues with quality score 0.694). **NOTE: This** 16% 3B08-G - **template was deliberately discarded:** Its total score 13.04 is lower than 30% of the best scoring X-ray template (534.80), and it newly

covers only 0 target residues. It is therefore highly unlikely that this template would yield a useful model.

3NHE- High Resolution Structure (1.26a) Of Usp2a In Complex With Ubiquitin (76 residues with resolution 1.260). **NOTE: This template was** 16% B - **deliberately discarded:** Its total score 12.88 is lower than 30% of the best scoring X-ray template (534.80), and it newly covers only 0

5 26.71 5 13.04 5 12.88

5 12.54

5 12.34 5 12.31 5 12.31 5 12.22 5 12.22 5 12.16 5 12.12

5 12.12

5 12.00 5 11.99 5 11.96 5 11.82 5 11.79 5 11.77 5 11.71 5 11.56 5 11.52

1e-14 4e-31 2e-29

2e-29

2e-29 4e-31 3e-29 2e-29 2e-29 4e-31 2e-29

2e-29

3e-29 2e-29 4e-31 2e-28 2e-29 2e-29 7e-28 2e-29 3e-26

16% 2J7Q-B -

target residues. It is therefore highly unlikely that this template would yield a useful model.

Crystal Structure Of The Ubiquitin-Specific Protease Encoded By Murine Cytomegalovirus Tegument Protein M48 In Complex With A Ubquitin-Based Suicide Substrate (75 residues with quality score 0.688). **NOTE: This template was deliberately discarded:** Its total score 12.54 is lower than 30% of the best scoring X-ray template (534.80), and it newly covers only 0 target residues. It is therefore highly unlikely that this template would yield a useful model.

1XD3- Crystal Structure Of Uchl3-Ubvme Complex (75 residues with quality score 0.677). **NOTE: This template was deliberately discarded:** Its 16% D - total score 12.34 is lower than 30% of the best scoring X-ray template (534.80), and it newly covers only 0 target residues. It is therefore

highly unlikely that this template would yield a useful model.

Crystal Structure Of The Mouse Hoil1-l-nzf In Complex With Linear Di- Ubiquitin (151 residues with quality score 0.655). **NOTE: This** 16% 3B0A-A - **template was deliberately discarded:** Its total score 12.31 is lower than 30% of the best scoring X-ray template (534.80), and it newly

covers only 0 target residues. It is therefore highly unlikely that this template would yield a useful model.

3H7P- Crystal Structure Of K63-Linked Di-Ubiquitin (75 residues with quality score 0.655). **NOTE: This template was deliberately discarded:** Its 16% A - total score 12.31 is lower than 30% of the best scoring X-ray template (534.80), and it newly covers only 0 target residues. It is therefore

highly unlikely that this template would yield a useful model.

2WWZ- Tab2 Nzf Domain In Complex With Lys63-Linked Di-Ubiquitin, P212121 (76 residues with resolution 1.400). **NOTE: This template was** 16% A - **deliberately discarded:** Its total score 12.22 is lower than 30% of the best scoring X-ray template (534.80), and it newly covers only 0

target residues. It is therefore highly unlikely that this template would yield a useful model.

2ZCC- Ubiquitin Crystallized Under High Pressure (71 residues with resolution 1.400). **NOTE: This template was deliberately discarded:** Its 16% C - total score 12.22 is lower than 30% of the best scoring X-ray template (534.80), and it newly covers only 0 target residues. It is therefore

highly unlikely that this template would yield a useful model.

3ZNZ- Crystal Structure Of Otulin Otu Domain (c129a) In Complex With Met1-di Ubiquitin (146 residues with quality score 0.647). **NOTE: This** 16% B - **template was deliberately discarded:** Its total score 12.16 is lower than 30% of the best scoring X-ray template (534.80), and it newly

covers only 0 target residues. It is therefore highly unlikely that this template would yield a useful model.

Crystal Structure Of The Mouse Tab2-Nzf In Complex With Lys63-Linked Di-Ubiquitin (77 residues with quality score 0.631). **NOTE: This** 17% 3A9J-B - **template was deliberately discarded:** Its total score 12.12 is lower than 30% of the best scoring X-ray template (534.80), and it newly

covers only 1 target residue. It is therefore highly unlikely that this template would yield a useful model.

Structural Analysis Of A Viral Otu Domain Protease From The Crimean- Congo Hemorrhagic Fever Virus In Complex With Human Ubiquitin 16% 3PRP- - (75 residues with quality score 0.665). **NOTE: This template was deliberately discarded:** Its total score 12.12 is lower than 30% of the

D best scoring X-ray template (534.80), and it newly covers only 0 target residues. It is therefore highly unlikely that this template would yield a useful model.

3ONS- Crystal Structure Of Human Ubiquitin In A New Crystal Form (72 residues with quality score 0.651). **NOTE: This template was** 15% A - **deliberately discarded:** Its total score 12.00 is lower than 30% of the best scoring X-ray template (534.80), and it newly covers only 0

target residues. It is therefore highly unlikely that this template would yield a useful model.

2ZNV- Crystal Structure Of Human Amsh-Lp Dub Domain In Complex With Lys63-Linked Ubiquitin Dimer (77 residues with quality score 0.624). 17% C - **NOTE: This template was deliberately discarded:** Its total score 11.99 is lower than 30% of the best scoring X-ray template (534.80),

and it newly covers only 0 target residues. It is therefore highly unlikely that this template would yield a useful model.

4KSL- Gumby/fam105b In Complex With Linear Di-ubiquitin (150 residues with quality score 0.657). **NOTE: This template was deliberately** 16% C - **discarded:** Its total score 11.96 is lower than 30% of the best scoring X-ray template (534.80), and it newly covers only 0 target residues. It

is therefore highly unlikely that this template would yield a useful model.

X-Ray Crystal Structure Of A Chemically Synthesized Ubiquitin (71 residues with quality score 0.675). **NOTE: This template was** 16% 1YIW-A - **deliberately discarded:** Its total score 11.82 is lower than 30% of the best scoring X-ray template (534.80), and it newly covers only 0

target residues. It is therefore highly unlikely that this template would yield a useful model.

3RUL- New Strategy To Analyze Structures Of Glycopeptide-Target Complexes (79 residues with quality score 0.609). **NOTE: This template was** 17% B - **deliberately discarded:** Its total score 11.79 is lower than 30% of the best scoring X-ray template (534.80), and it newly covers only 0

target residues. It is therefore highly unlikely that this template would yield a useful model.

Equine Arteritis Virus Papain-like Protease 2 (plp2) Covalently Bound To Ubiquitin (75 residues with resolution 1.450). **NOTE: This** 16% 4IUM-B - **template was deliberately discarded:** Its total score 11.77 is lower than 30% of the best scoring X-ray template (534.80), and it newly

covers only 0 target residues. It is therefore highly unlikely that this template would yield a useful model.

2ZCB- Crystal Structure Of Ubiquitin P37aP38A (73 residues with quality score 0.669). **NOTE: This template was deliberately discarded:** Its 16% B - total score 11.71 is lower than 30% of the best scoring X-ray template (534.80), and it newly covers only 0 target residues. It is therefore

highly unlikely that this template would yield a useful model.

3H7S- Crystal Structures Of K63-Linked Di- And Tri-Ubiquitin Reveal A Highly Extended Chain Architecture (75 residues with quality score 0.634). 16% A - **NOTE: This template was deliberately discarded:** Its total score 11.56 is lower than 30% of the best scoring X-ray template (534.80),

and it newly covers only 0 target residues. It is therefore highly unlikely that this template would yield a useful model.

4GU2- Crystal Structure Of Ubiquitin From Entamoeba Histolytica To 1.35 Angstrom (68 residues with quality score 0.600). **NOTE: This template** 15% A - **was deliberately discarded:** Its total score 11.52 is lower than 30% of the best scoring X-ray template (534.80), and it newly covers only 0

5 11.52 5 11.52 5 11.49 5 11.41 5 11.40 5 11.40 5 11.36 5 11.30 5 11.16 5 11.02 5 11.00 5 11.00 5 10.93

5 10.85 5 10.78

5 10.78 5 10.74 5 10.73 5 10.63 5 10.62 5 10.58 5 10.54

3e-26 2e-29 2e-29 1e-28 3e-26 2e-29 3e-28 2e-29 3e-29 1e-14 2e-29 2e-29 2e-28

2e-27 2e-29

7e-29 2e-27 2e-29 4e-21 7e-29 5e-26 1e-25

124.0 15% A -

3K9O- 113.0 16% B -

116.0 17% 3A9K-B -

3CMM- 121.0 16% D -

4GSW- 124.0 15% B -

3DVN- 116.0 16% X -

108.0 16% 4I6N-D -

3NOB- 113.0 16% A -

3DVN- 115.0 16% Y -

1MG8- 337.0 16% A -

116.0 16% 3LDZ-E -

115.0 16% 4AP4-F -

1S1Q- 110.0 16% -

**was deliberately discarded:** Its total score 11.52 is lower than 30% of the best scoring X-ray template (534.80), and it newly covers only 0 target residues. It is therefore highly unlikely that this template would yield a useful model.

The Crystal Structure Of E2-25k And Ubb+1 Complex (75 residues with quality score 0.632). **NOTE: This template was deliberately discarded:** Its total score 11.52 is lower than 30% of the best scoring X-ray template (534.80), and it newly covers only 0 target residues. It is therefore highly unlikely that this template would yield a useful model.

Crystal Structure Of The Mouse Tab3-Nzf In Complex With Lys63-Linked Di-Ubiquitin (77 residues with quality score 0.598). **NOTE: This template was deliberately discarded:** Its total score 11.49 is lower than 30% of the best scoring X-ray template (534.80), and it newly covers only 0 target residues. It is therefore highly unlikely that this template would yield a useful model.

Crystal Structure Of The Uba1-Ubiquitin Complex (76 residues with quality score 0.577). **NOTE: This template was deliberately discarded:** Its total score 11.41 is lower than 30% of the best scoring X-ray template (534.80), and it newly covers only 0 target residues. It is therefore highly unlikely that this template would yield a useful model.

Crystal Structure Of Ubiquitin From Entamoeba Histolytica To 2.15 Angstrom (73 residues with quality score 0.594). **NOTE: This template was deliberately discarded:** Its total score 11.40 is lower than 30% of the best scoring X-ray template (534.80), and it newly covers only 0 target residues. It is therefore highly unlikely that this template would yield a useful model.

Crystal Structure Of K63-specific Fab Apu2.16 Bound To K63-linked Di- Ubiquitin (75 residues with quality score 0.626). **NOTE: This template was deliberately discarded:** Its total score 11.40 is lower than 30% of the best scoring X-ray template (534.80), and it newly covers only 0 target residues. It is therefore highly unlikely that this template would yield a useful model.

Crystal Structure Of Trichinella Spiralis Uch37 Catalytic Domain Bound To Ubiquitin Vinyl Methyl Ester (75 residues with quality score 0.652). **NOTE: This template was deliberately discarded:** Its total score 11.36 is lower than 30% of the best scoring X-ray template (534.80), and it newly covers only 0 target residues. It is therefore highly unlikely that this template would yield a useful model.

Structure Of K11-Linked Di-Ubiquitin (77 residues with quality score 0.620). **NOTE: This template was deliberately discarded:** Its total score 11.30 is lower than 30% of the best scoring X-ray template (534.80), and it newly covers only 0 target residues. It is therefore highly unlikely that this template would yield a useful model.

Crystal Structure Of K63-specific Fab Apu2.16 Bound To K63-linked Di- Ubiquitin (76 residues with quality score 0.594). **NOTE: This template was deliberately discarded:** Its total score 11.16 is lower than 30% of the best scoring X-ray template (534.80), and it newly covers only 0 target residues. It is therefore highly unlikely that this template would yield a useful model.

Nmr Structure Of Ubiquitin-Like Domain In Murine Parkin (78 residues with quality score 0.200). **NOTE: This template was deliberately discarded:** Its total score 11.02 is lower than 30% of the best scoring X-ray template (534.80), and it newly covers only 0 target residues. It is therefore highly unlikely that this template would yield a useful model.

Crystal Structure Of Human Stam1 Vhs Domain In Complex With Ubiquitin (73 residues with quality score 0.604). **NOTE: This template was deliberately discarded:** Its total score 11.00 is lower than 30% of the best scoring X-ray template (534.80), and it newly covers only 0 target residues. It is therefore highly unlikely that this template would yield a useful model.

Rnf4 - Ubch5a - Ubiquitin Heterotrimeric Complex (77 residues with quality score 0.585). **NOTE: This template was deliberately discarded:** Its total score 11.00 is lower than 30% of the best scoring X-ray template (534.80), and it newly covers only 0 target residues. It is therefore highly unlikely that this template would yield a useful model.

Tsg101(Uev) Domain In Complex With Ubiquitin (76 residues with quality score 0.608). **NOTE: This template was deliberately discarded:** Its total score 10.93 is lower than 30% of the best scoring X-ray template (534.80), and it newly covers only 0 target residues. It

D is therefore highly unlikely that this template would yield a useful model.

105.0 16% 116.0 16%

95.0 32% 114.0 16% 113.0 16% 106.0 16% 95.0 32% 119.0 16% 117.0 15%

2FCS- B -

X-Ray Crystal Structure Of A Chemically Synthesized [l-Gln35]ubiquitin With A Cubic Space Group (73 residues with quality score 0.658). **NOTE: This template was deliberately discarded:** Its total score 10.85 is lower than 30% of the best scoring X-ray template (534.80), and it newly covers only 0 target residues. It is therefore highly unlikely that this template would yield a useful model.

X-Ray Structure Of The Non-Covalent Complex Between Ubch5a And Ubiquitin (74 residues with quality score 0.592). **NOTE: This**

3PTF- - D covers only 0 target residues. It is therefore highly unlikely that this template would yield a useful model.

3L0W- B -

3V6E-B - 4FJV-D - 1BT0-A - 3L10-B - 4II2-B - 1SIF-A -

**template was deliberately discarded:** Its total score 10.78 is lower than 30% of the best scoring X-ray template (534.80), and it newly Structure Of Split Monoubiquitinated Pcna With Ubiquitin In Position Two (166 residues with quality score 0.354). **NOTE: This template**

**was deliberately discarded:** Its total score 10.78 is lower than 30% of the best scoring X-ray template (534.80), and it newly covers only 3 target residues. It is therefore highly unlikely that this template would yield a useful model.

Crystal Structure Of Usp2 And A Mutant Form Of Ubiquitin (81 residues with quality score 0.592). **NOTE: This template was deliberately discarded:** Its total score 10.74 is lower than 30% of the best scoring X-ray template (534.80), and it newly covers only 0 target residues. It is therefore highly unlikely that this template would yield a useful model.

Crystal Structure Of Human Otubain2 And Ubiquitin Complex (82 residues with quality score 0.589). **NOTE: This template was deliberately discarded:** Its total score 10.73 is lower than 30% of the best scoring X-ray template (534.80), and it newly covers only 0 target residues. It is therefore highly unlikely that this template would yield a useful model.

Structure Of Ubiquitin-Like Protein, Rub1 (73 residues with quality score 0.639). **NOTE: This template was deliberately discarded:** Its total score 10.63 is lower than 30% of the best scoring X-ray template (534.80), and it newly covers only 0 target residues. It is therefore highly unlikely that this template would yield a useful model.

Structure Of Split Monoubiquitinated Pcna With Ubiquitin In Position One (166 residues with quality score 0.349). **NOTE: This template was deliberately discarded:** Its total score 10.62 is lower than 30% of the best scoring X-ray template (534.80), and it newly covers only 0 target residues. It is therefore highly unlikely that this template would yield a useful model.

Crystal Structure Of Ubiquitin Activating Enzyme 1 (uba1) In Complex With The Ub E2 Ubc4, Ubiquitin, And Atp/mg (80 residues with quality score 0.544). **NOTE: This template was deliberately discarded:** Its total score 10.58 is lower than 30% of the best scoring X-ray template (534.80), and it newly covers only 0 target residues. It is therefore highly unlikely that this template would yield a useful model.

Crystal Structure Of A Multiple Hydrophobic Core Mutant Of Ubiquitin (71 residues with quality score 0.590). **NOTE: This template was deliberately discarded:** Its total score 10.54 is lower than 30% of the best scoring X-ray template (534.80), and it newly covers only 0 target residues. It is therefore highly unlikely that this template would yield a useful model.

Crystal Structure Of The 9-10 8 Glycine Insertion Mutant Of Ubiquitin (84 residues with quality score 0.614). **NOTE: This template was**

5 10.44

2e-26

104.0

112.0 121.0 113.0 104.0 110.0 116.0 116.0 110.0 397.0 116.0 104.0 98.0 116.0 113.0 102.0 104.0 113.0 99.0 115.0 121.0 114.0

16% 2GBJ- - B

16% 3ZLZ-A -

4HCN- 16% B -

16% 2Y5B-F -

16% 1YJ1-C -

4BBN- 16% F -

2W9N- 16% A -

3AXC- 16% A -

3V6C- 16% B -

16% 1IYF-A -

3DVG- 16% X -

2FCM- 16% A -

2GBM- 16% B -

16% 3OJ3-D -

3MHS- 16% D -

4HK2- 16% A -

2GBR- 15% A -

16% 4LDT-B -

2GBK- 16% C -

4KSK- 16% C -

16% 2JT4-B - 15% 3AI5-A -

Crystal Structure Of The 9-10 8 Glycine Insertion Mutant Of Ubiquitin (84 residues with quality score 0.614). **NOTE: This template was deliberately discarded:** Its total score 10.44 is lower than 30% of the best scoring X-ray template (534.80), and it newly covers only 0 target residues. It is therefore highly unlikely that this template would yield a useful model.

Lys6-linked Tri-ubiquitin (73 residues with quality score 0.569). **NOTE: This template was deliberately discarded:** Its total score 10.42 is lower than 30% of the best scoring X-ray template (534.80), and it newly covers only 0 target residues. It is therefore highly unlikely that this template would yield a useful model.

Crystal Structure Of Burkholderia Pseudomallei Effector Protein Chbp In Complex With Ubiquitin (77 residues with quality score 0.524). **NOTE: This template was deliberately discarded:** Its total score 10.36 is lower than 30% of the best scoring X-ray template (534.80), and it newly covers only 0 target residues. It is therefore highly unlikely that this template would yield a useful model.

Structure Of Usp21 In Complex With Linear Diubiquitin-Aldehyde (152 residues with quality score 0.561). **NOTE: This template was deliberately discarded:** Its total score 10.36 is lower than 30% of the best scoring X-ray template (534.80), and it newly covers only 0 target residues. It is therefore highly unlikely that this template would yield a useful model.

X-Ray Crystal Structure Of A Chemically Synthesized [d-Gln35]ubiquitin (74 residues with quality score 0.624). **NOTE: This template was deliberately discarded:** Its total score 10.33 is lower than 30% of the best scoring X-ray template (534.80), and it newly covers only 0 target residues. It is therefore highly unlikely that this template would yield a useful model.

Nedd4 Hect-ub:ub Complex (76 residues with quality score 0.568). **NOTE: This template was deliberately discarded:** Its total score 10.21 is lower than 30% of the best scoring X-ray template (534.80), and it newly covers only 0 target residues. It is therefore highly unlikely that this template would yield a useful model.

Crystal Structure Of Linear Di-Ubiquitin (149 residues with quality score 0.560). **NOTE: This template was deliberately discarded:** Its total score 10.20 is lower than 30% of the best scoring X-ray template (534.80), and it newly covers only 0 target residues. It is therefore highly unlikely that this template would yield a useful model.

Crystal Structure Of Linear Diubiquitin (149 residues with quality score 0.556). **NOTE: This template was deliberately discarded:** Its total score 10.13 is lower than 30% of the best scoring X-ray template (534.80), and it newly covers only 0 target residues. It is therefore highly unlikely that this template would yield a useful model.

Crystal Structure Of Usp2 In Complex With Mutated Ubiquitin (81 residues with quality score 0.576). **NOTE: This template was deliberately discarded:** Its total score 10.08 is lower than 30% of the best scoring X-ray template (534.80), and it newly covers only 0 target residues. It is therefore highly unlikely that this template would yield a useful model.

Solution Structure Of Ubiquitin-Like Domain Of Human Parkin (76 residues with quality score 0.155). **NOTE: This template was deliberately discarded:** Its total score 10.06 is lower than 30% of the best scoring X-ray template (534.80), and it newly covers only 0 target residues. It is therefore highly unlikely that this template would yield a useful model.

Crystal Structure Of K63-Specific Fab Apu.3a8 Bound To K63-Linked Di- Ubiquitin (75 residues with quality score 0.545). **NOTE: This template was deliberately discarded:** Its total score 9.92 is lower than 30% of the best scoring X-ray template (534.80), and it newly covers only 0 target residues. It is therefore highly unlikely that this template would yield a useful model.

X-Ray Crystal Structure Of A Chemically Synthesized [d-Gln35]ubiquitin With A Cubic Space Group (73 residues with quality score 0.597). **NOTE: This template was deliberately discarded:** Its total score 9.88 is lower than 30% of the best scoring X-ray template (534.80), and it newly covers only 0 target residues. It is therefore highly unlikely that this template would yield a useful model.

Crystal Structure Of The 35-36 8 Glycine Insertion Mutant Of Ubiquitin (80 residues with quality score 0.638). **NOTE: This template was deliberately discarded:** Its total score 9.82 is lower than 30% of the best scoring X-ray template (534.80), and it newly covers only 0 target residues. It is therefore highly unlikely that this template would yield a useful model.

Crystal Structure Of The A20 Znf4 And Ubiquitin Complex (73 residues with quality score 0.537). **NOTE: This template was deliberately discarded:** Its total score 9.78 is lower than 30% of the best scoring X-ray template (534.80), and it newly covers only 0 target residues. It is therefore highly unlikely that this template would yield a useful model.

Structure Of The Saga Ubp8SGF11SUS1SGF73 DUB MODULE BOUND Ubiquitin Aldehyde (76 residues with resolution 1.890). **NOTE: This template was deliberately discarded:** Its total score 9.74 is lower than 30% of the best scoring X-ray template (534.80), and it newly covers only 0 target residues. It is therefore highly unlikely that this template would yield a useful model.

U7ub25.2540 (74 residues with quality score 0.599). **NOTE: This template was deliberately discarded:** Its total score 9.72 is lower than 30% of the best scoring X-ray template (534.80), and it newly covers only 0 target residues. It is therefore highly unlikely that this template would yield a useful model.

Crystal Structure Of The 35-36 Moad Insertion Mutant Of Ubiquitin (77 residues with quality score 0.603). **NOTE: This template was deliberately discarded:** Its total score 9.71 is lower than 30% of the best scoring X-ray template (534.80), and it newly covers only 0 target residues. It is therefore highly unlikely that this template would yield a useful model.

The Structure Of H/ceotub1-ubiquitin Aldehyde-ubch5b~ub (76 residues with resolution 1.900). **NOTE: This template was deliberately discarded:** Its total score 9.70 is lower than 30% of the best scoring X-ray template (534.80), and it newly covers only 0 target residues. It is therefore highly unlikely that this template would yield a useful model.

Crystal Structure Of The 9-10 Moad Insertion Mutant Of Ubiquitin (83 residues with quality score 0.599). **NOTE: This template was deliberately discarded:** Its total score 9.69 is lower than 30% of the best scoring X-ray template (534.80), and it newly covers only 0 target residues. It is therefore highly unlikely that this template would yield a useful model.

Gumby/fam105b In Complex With Ubiquitin (75 residues with quality score 0.515). **NOTE: This template was deliberately discarded:** Its total score 9.68 is lower than 30% of the best scoring X-ray template (534.80), and it newly covers only 0 target residues. It is therefore highly unlikely that this template would yield a useful model.

Solution Structure Of The Sla1 Sh3-3-Ubiquitin Complex (76 residues with quality score 0.488). **NOTE: This template was deliberately discarded:** Its total score 9.65 is lower than 30% of the best scoring X-ray template (534.80), and it newly covers only 0 target residues. It is therefore highly unlikely that this template would yield a useful model.

Crystal Structure Of Yeast Enhanced Green Fluorescent Protein- Ubiquitin Fusion Protein (299 residues with quality score 0.552). **NOTE: This template was deliberately discarded:** Its total score 9.61 is lower than 30% of the best scoring X-ray template (534.80), and it newly covers only 0 target residues. It is therefore highly unlikely that this template would yield a useful model.

Crystal Structure And Solution Nmr Studies Of Lys48-Linked Tetraubiquitin At Neutral Ph (76 residues with quality score 0.524). **NOTE:**

5 10.42 5 10.36 5 10.36 5 10.33 5 10.21 5 10.20 5 10.13 5 10.08 5 10.06 5 9.92 5 9.88 5 9.82 5 9.78 5 9.74 5 9.72 5 9.71 5 9.70 5 9.69 5 9.68 5 9.65 5 9.61

3e-29 1e-28 3e-31 2e-27 2e-29 5e-30 4e-31 6e-27 3e-15 2e-29 2e-27 2e-26 2e-29 2e-29 7e-24 1e-27 2e-29 1e-27 2e-29 1e-28 5e-29

5 9.59 5 9.57 5 9.53 5 9.50 5 9.47

5 9.28

5 9.19 5 9.13 5 9.11 5 9.00 5 8.96 5 8.91 5 8.79 5 8.77 5 8.75 5 8.75 5 8.56 5 8.55 5 8.54 5 8.49 5 8.44 5 8.41

5e-29 3e-07 1e-28 2e-27 2e-29

2e-28

5e-29 2e-29 2e-29 2e-28 1e-28 3e-29 2e-20 4e-31 1e-23 9e-29 1e-28 2e-29 2e-29 2e-26 2e-20 2e-29

112.0 95.0 121.0 104.0 115.0

107.0

110.0 115.0 113.0 110.0 119.0 112.0 88.0 114.0 103.0 107.0 121.0 115.0 116.0 98.0 86.0 115.0

2O6V- 16% D -

2BWF- 16% A -

16% 4II3-B -

2FCN- 16% B -

16% 1YX6-B -

3Q3F- 18% A -

1OGW- 16% A -

16% 1YX5-B -

3VDZ- 16% B -

2FCQ- 16% A -

3OLM- 16% D -

16% 1ZGU- - B

1NDD- 16% B -

15% 3U30-A - 16% 4HJK-A - 16% 2XK5-B -

1Q0W- 16% B -

4AUQ- 16% C -

16% 3K9P-B -

2GBN- 16% A -

15% 4FBJ-B - 16% 3ZNI-D -

Crystal Structure And Solution Nmr Studies Of Lys48-Linked Tetraubiquitin At Neutral Ph (76 residues with quality score 0.524). **NOTE: This template was deliberately discarded:** Its total score 9.59 is lower than 30% of the best scoring X-ray template (534.80), and it newly covers only 0 target residues. It is therefore highly unlikely that this template would yield a useful model.

Crystal Sturcture Of The Ubl Domain Of Dsk2 From S. Cerevisiae (77 residues with quality score 0.642). **NOTE: This template was deliberately discarded:** Its total score 9.57 is lower than 30% of the best scoring X-ray template (534.80), and it newly covers only 0 target residues. It is therefore highly unlikely that this template would yield a useful model.

Crystal Structure Of S. Pombe Ubiquitin Activating Enzyme 1 (uba1) In Complex With Ubiquitin And Atp/mg (76 residues with quality score 0.482). **NOTE: This template was deliberately discarded:** Its total score 9.53 is lower than 30% of the best scoring X-ray template (534.80), and it newly covers only 0 target residues. It is therefore highly unlikely that this template would yield a useful model.

X-Ray Crystal Structure Of A Chemically Synthesized [d-Val35]ubiquitin With A Cubic Space Group (73 residues with quality score 0.574). **NOTE: This template was deliberately discarded:** Its total score 9.50 is lower than 30% of the best scoring X-ray template (534.80), and it newly covers only 0 target residues. It is therefore highly unlikely that this template would yield a useful model.

Solution Structure Of S5a Uim-2UBIQUITIN COMPLEX (76 residues with quality score 0.504). **NOTE: This template was deliberately discarded:** Its total score 9.47 is lower than 30% of the best scoring X-ray template (534.80), and it newly covers only 0 target residues. It is therefore highly unlikely that this template would yield a useful model.

Engineering Domain-Swapped Binding Interfaces By Mutually Exclusive Folding: Insertion Of Ubiquitin Into Position 103 Of Barnase (188 residues with quality score 0.480). **NOTE: This template was deliberately discarded:** Its total score 9.28 is lower than 30% of the best

scoring X-ray template (534.80), and it newly covers only 0 target residues. It is therefore highly unlikely that this template would yield a useful model.

Synthetic Ubiquitin With Fluoro-Leu At 50 And 67 (76 residues with quality score 0.511). **NOTE: This template was deliberately discarded:** Its total score 9.19 is lower than 30% of the best scoring X-ray template (534.80), and it newly covers only 0 target residues. It is therefore highly unlikely that this template would yield a useful model.

Solution Structure Of S5a Uim-1UBIQUITIN COMPLEX (76 residues with quality score 0.486). **NOTE: This template was deliberately discarded:** Its total score 9.13 is lower than 30% of the best scoring X-ray template (534.80), and it newly covers only 0 target residues. It is therefore highly unlikely that this template would yield a useful model.

Tailoring Encodable Lanthanide-Binding Tags As Mri Contrast Agents: Xq-Dse3-Ubiquitin At 2.4 Angstroms (110 residues with quality score 0.500). **NOTE: This template was deliberately discarded:** Its total score 9.11 is lower than 30% of the best scoring X-ray template (534.80), and it newly covers only 0 target residues. It is therefore highly unlikely that this template would yield a useful model.

X-Ray Crystal Structure Of A Chemically Synthesized Ubiquitin With A Cubic Space Group (73 residues with quality score 0.514). **NOTE: This template was deliberately discarded:** Its total score 9.00 is lower than 30% of the best scoring X-ray template (534.80), and it newly covers only 0 target residues. It is therefore highly unlikely that this template would yield a useful model.

Structure And Function Of A Ubiquitin Binding Site Within The Catalytic Domain Of A Hect Ubiquitin Ligase (74 residues with quality score 0.473). **NOTE: This template was deliberately discarded:** Its total score 8.96 is lower than 30% of the best scoring X-ray template (534.80), and it newly covers only 0 target residues. It is therefore highly unlikely that this template would yield a useful model.

Solution Structure Of The Human Mms2-Ubiquitin Complex (76 residues with quality score 0.487). **NOTE: This template was deliberately discarded:** Its total score 8.91 is lower than 30% of the best scoring X-ray template (534.80), and it newly covers only 0 target residues. It is therefore highly unlikely that this template would yield a useful model.

Structure Of Nedd8 (76 residues with quality score 0.611). **NOTE: This template was deliberately discarded:** Its total score 8.79 is lower than 30% of the best scoring X-ray template (534.80), and it newly covers only 0 target residues. It is therefore highly unlikely that this template would yield a useful model.

Crystal Structure Of A Linear-Specific Ubiquitin Fab Bound To Linear Ubiquitin (149 residues with quality score 0.504). **NOTE: This template was deliberately discarded:** Its total score 8.77 is lower than 30% of the best scoring X-ray template (534.80), and it newly covers only 0 target residues. It is therefore highly unlikely that this template would yield a useful model.

U7ub7 Disulfide Variant (77 residues with quality score 0.520). **NOTE: This template was deliberately discarded:** Its total score 8.75 is lower than 30% of the best scoring X-ray template (534.80), and it newly covers only 0 target residues. It is therefore highly unlikely that this template would yield a useful model.

Crystal Structure Of K6-Linked Diubiquitin (75 residues with quality score 0.507). **NOTE: This template was deliberately discarded:** Its total score 8.75 is lower than 30% of the best scoring X-ray template (534.80), and it newly covers only 0 target residues. It is therefore highly unlikely that this template would yield a useful model.

Solution Structure Of Vps27 Amino-Terminal Uim-Ubiquitin Complex (76 residues with quality score 0.433). **NOTE: This template was deliberately discarded:** Its total score 8.56 is lower than 30% of the best scoring X-ray template (534.80), and it newly covers only 0 target residues. It is therefore highly unlikely that this template would yield a useful model.

Structure Of Birc7-Ubch5b-Ub Complex. (77 residues with resolution 2.180). **NOTE: This template was deliberately discarded:** Its total score 8.55 is lower than 30% of the best scoring X-ray template (534.80), and it newly covers only 0 target residues. It is therefore highly unlikely that this template would yield a useful model.

The Crystal Structure Of E2-25k And Ubiquitin Complex (72 residues with quality score 0.469). **NOTE: This template was deliberately discarded:** Its total score 8.54 is lower than 30% of the best scoring X-ray template (534.80), and it newly covers only 0 target residues. It is therefore highly unlikely that this template would yield a useful model.

Crystal Structure Of The 35-36 8 Glycine Insertion Mutant Of Ubiquitin (81 residues with quality score 0.552). **NOTE: This template was deliberately discarded:** Its total score 8.49 is lower than 30% of the best scoring X-ray template (534.80), and it newly covers only 0 target residues. It is therefore highly unlikely that this template would yield a useful model.

Structure Of The Cif:nedd8 Complex - Photorhabdus Luminescens Cycle Inhibiting Factor In Complex With Human Nedd8 (74 residues with quality score 0.634). **NOTE: This template was deliberately discarded:** Its total score 8.44 is lower than 30% of the best scoring X- ray template (534.80), and it newly covers only 0 target residues. It is therefore highly unlikely that this template would yield a useful model.

Structure Of Phosphotyr363-cbl-b - Ubch5b-ub - Zap-70 Peptide Complex (77 residues with resolution 2.210). **NOTE: This template was deliberately discarded:** Its total score 8.41 is lower than 30% of the best scoring X-ray template (534.80), and it newly covers only 0 target

5 8.41 5 8.40 5 8.36

5 8.35 5 8.30 5 8.28 5 8.18

5 8.07

5 7.98 5 7.91 5 7.78 5 7.67 5 7.66 5 7.64 5 7.64

5 7.58

5 7.56 5 7.44 5 7.38 5 7.24 5 7.17 5 7.14

2e-29 4e-31 7e-24

0.15 9e-28 4e-27 2e-20

2e-29

6e-29 3e-29 1.5 1.2 2e-29 3e-20 2e-29

1.7

2e-20 2e-29 2e-20 1.7 4e-06 4e-31

115.0 16% 3ZNI-D -

2ZVO- 116.0 16% G -

3N3K- 87.0 16% B -

3RZW- 86.0 16% C -

97.0 16% 4I6L-B -

3MTN- 99.0 16% D -

2BKR- 88.0 16% B -

110.0 16% 4DHJ- - D

112.0 16% 4JIO-U -

3DVG- 115.0 16% Y -

82.0 16% 3UIP-B -

1WYW- 82.0 16% B -

2K6D- 110.0 16% B -

4HCP- 89.0 16% B -

2OJR- 113.0 16% A -

82.0 16% 2VRR- - B

86.0 15% 4F8C-B -

3VFK- 114.0 17% A -

88.0 16% 1XT9-B -

2UYZ- 82.0 16% B -

4EEW- 72.0 15% B -

2ZVN- 116.0 16% G -

**deliberately discarded:** Its total score 8.41 is lower than 30% of the best scoring X-ray template (534.80), and it newly covers only 0 target residues. It is therefore highly unlikely that this template would yield a useful model.

Nemo Cozi Domain In Complex With Diubiquitin In C2 Space Group (146 residues with quality score 0.461). **NOTE: This template was deliberately discarded:** Its total score 8.40 is lower than 30% of the best scoring X-ray template (534.80), and it newly covers only 0 target residues. It is therefore highly unlikely that this template would yield a useful model.

The Catalytic Domain Of Usp8 In Complex With A Usp8 Specific Inhibitor (74 residues with quality score 0.604). **NOTE: This template was deliberately discarded:** Its total score 8.36 is lower than 30% of the best scoring X-ray template (534.80), and it newly covers only 0

target residues. It is therefore highly unlikely that this template would yield a useful model. Crystal Structure Of The Monobody Ysmb-9 Bound To Human Sumo1 (75 residues with quality score 0.610). **NOTE: This template was**

**deliberately discarded:** Its total score 8.35 is lower than 30% of the best scoring X-ray template (534.80), and it newly covers only 1 target residue. It is therefore highly unlikely that this template would yield a useful model.

Crystal Structure Of Otub1 In Complex With Ubiquitin Variant (75 residues with quality score 0.545). **NOTE: This template was deliberately discarded:** Its total score 8.30 is lower than 30% of the best scoring X-ray template (534.80), and it newly covers only 0 target residues. It is therefore highly unlikely that this template would yield a useful model.

Usp21 In Complex With A Ubiquitin-based, Usp21-specific Inhibitor (77 residues with quality score 0.512). **NOTE: This template was deliberately discarded:** Its total score 8.28 is lower than 30% of the best scoring X-ray template (534.80), and it newly covers only 0 target residues. It is therefore highly unlikely that this template would yield a useful model.

Nedd8 Nedp1 Complex (77 residues with quality score 0.569). **NOTE: This template was deliberately discarded:** Its total score 8.18 is lower than 30% of the best scoring X-ray template (534.80), and it newly covers only 0 target residues. It is therefore highly unlikely that this template would yield a useful model.

The Structure Of A Ceotub1 Ubiquitin Aldehyde Ubc13~ub Complex (71 residues with quality score 0.449). **NOTE: This template was deliberately discarded:** Its total score 8.07 is lower than 30% of the best scoring X-ray template (534.80), and it newly covers only 0 target residues. It is therefore highly unlikely that this template would yield a useful model.

Bro1 V Domain And Ubiquitin (75 residues with quality score 0.442). **NOTE: This template was deliberately discarded:** Its total score 7.98 is lower than 30% of the best scoring X-ray template (534.80), and it newly covers only 0 target residues. It is therefore highly unlikely that this template would yield a useful model.

Crystal Structure Of K63-Specific Fab Apu.3a8 Bound To K63-Linked Di- Ubiquitin (76 residues with quality score 0.421). **NOTE: This template was deliberately discarded:** Its total score 7.91 is lower than 30% of the best scoring X-ray template (534.80), and it newly covers only 0 target residues. It is therefore highly unlikely that this template would yield a useful model.

Complex Between Human Rangap1-sumo1, Ubc9 And The Ir1 Domain From Ranbp2 Containing Ir2 Motif Ii (78 residues with quality score 0.596). **NOTE: This template was deliberately discarded:** Its total score 7.78 is lower than 30% of the best scoring X-ray template (534.80), and it newly covers only 0 target residues. It is therefore highly unlikely that this template would yield a useful model.

Crystal Structure Of Sumo1-Conjugated Thymine Dna Glycosylase (79 residues with quality score 0.588). **NOTE: This template was deliberately discarded:** Its total score 7.67 is lower than 30% of the best scoring X-ray template (534.80), and it newly covers only 0 target residues. It is therefore highly unlikely that this template would yield a useful model.

Cin85 Sh3-C Domain In Complex With Ubiquitin (76 residues with quality score 0.426). **NOTE: This template was deliberately discarded:** Its total score 7.66 is lower than 30% of the best scoring X-ray template (534.80), and it newly covers only 0 target residues. It is therefore highly unlikely that this template would yield a useful model.

Crystal Structure Of Burkholderia Pseudomallei Effector Protein Chbp In Complex With Nedd8 (75 residues with quality score 0.547). **NOTE: This template was deliberately discarded:** Its total score 7.64 is lower than 30% of the best scoring X-ray template (534.80), and it newly covers only 0 target residues. It is therefore highly unlikely that this template would yield a useful model.

Structure Of Ubiquitin Solved By Sad Using The Lanthanide- Binding Tag (110 residues with quality score 0.419). **NOTE: This template was deliberately discarded:** Its total score 7.64 is lower than 30% of the best scoring X-ray template (534.80), and it newly covers only 0 target residues. It is therefore highly unlikely that this template would yield a useful model.

Structure Of Sumo Modified Ubc9 (79 residues with quality score 0.581). **NOTE: This template was deliberately discarded:** Its total score 7.58 is lower than 30% of the best scoring X-ray template (534.80), and it newly covers only 0 target residues. It is therefore highly unlikely that this template would yield a useful model.

Structure Of The Cif:nedd8 Complex - Yersinia Pseudotuberculosis Cycle Inhibiting Factor In Complex With Human Nedd8 (74 residues with quality score 0.568). **NOTE: This template was deliberately discarded:** Its total score 7.56 is lower than 30% of the best scoring X- ray template (534.80), and it newly covers only 0 target residues. It is therefore highly unlikely that this template would yield a useful model.

The Structure Of Monodechloro-teicoplanin In Complex With Its Ligand, Using Ubiquitin As A Ligand Carrier (79 residues with quality score 0.384). **NOTE: This template was deliberately discarded:** Its total score 7.44 is lower than 30% of the best scoring X-ray template (534.80), and it newly covers only 0 target residues. It is therefore highly unlikely that this template would yield a useful model.

Crystal Structure Of Den1 In Complex With Nedd8 (76 residues with quality score 0.513). **NOTE: This template was deliberately discarded:** Its total score 7.38 is lower than 30% of the best scoring X-ray template (534.80), and it newly covers only 0 target residues. It is therefore highly unlikely that this template would yield a useful model.

Non-Covalent Complex Between Ubc9 And Sumo1 (78 residues with quality score 0.555). **NOTE: This template was deliberately discarded:** Its total score 7.24 is lower than 30% of the best scoring X-ray template (534.80), and it newly covers only 0 target residues. It is therefore highly unlikely that this template would yield a useful model.

Crystal Structure Of The Ubl Domain Of Bag6 (75 residues with quality score 0.652). **NOTE: This template was deliberately discarded:** Its total score 7.17 is lower than 30% of the best scoring X-ray template (534.80), and it newly covers only 0 target residues. It is therefore highly unlikely that this template would yield a useful model.

Nemo Cozi Domain Incomplex With Diubiquitin In P212121 Space Group (146 residues with quality score 0.392). **NOTE: This template was deliberately discarded:** Its total score 7.14 is lower than 30% of the best scoring X-ray template (534.80), and it newly covers only 0 target residues. It is therefore highly unlikely that this template would yield a useful model.

Solution Structure Of The Designed Hydrophobic Core Mutant Of Ubiquitin, 1d7 (76 residues with quality score 0.381). **NOTE: This**

Solution Structure Of The Designed Hydrophobic Core Mutant Of Ubiquitin, 1d7 (76 residues with quality score 0.381). **NOTE: This template was deliberately discarded:** Its total score 7.10 is lower than 30% of the best scoring X-ray template (534.80), and it newly covers only 0 target residues. It is therefore highly unlikely that this template would yield a useful model.

Structure Of Appbp1-Uba3~nedd8-Nedd8-Mgatp-Ubc12(C111a), A Trapped Ubiquitin-Like Protein Activation Complex (76 residues with quality score 0.493). **NOTE: This template was deliberately discarded:** Its total score 7.09 is lower than 30% of the best scoring X-ray template (534.80), and it newly covers only 0 target residues. It is therefore highly unlikely that this template would yield a useful model.

Crystal Structure Of A Hla-B Associated Transcript 3 (Bat3) From Homo Sapiens At 1.80 A Resolution (79 residues with quality score 0.645). **NOTE: This template was deliberately discarded:** Its total score 6.79 is lower than 30% of the best scoring X-ray template (534.80), and it newly covers only 0 target residues. It is therefore highly unlikely that this template would yield a useful model.

Rotamer Strain As A Determinant Of Protein Structural Specificity (76 residues with quality score 0.338). **NOTE: This template was deliberately discarded:** Its total score 6.57 is lower than 30% of the best scoring X-ray template (534.80), and it newly covers only 0 target residues. It is therefore highly unlikely that this template would yield a useful model.

Mutations In The Hydrophobic Core Of Ubiquitin Differentially Affect Its Recognition By Receptor Proteins (76 residues with quality score 0.341). **NOTE: This template was deliberately discarded:** Its total score 6.52 is lower than 30% of the best scoring X-ray template (534.80), and it newly covers only 0 target residues. It is therefore highly unlikely that this template would yield a useful model.

Structural Dissection Of A Gating Mechanism Preventing Misactivation Of Ubiquitin By Nedd8's E1 (Appbp1- Uba3arg190gln-

5 7.10 5 7.09 5 6.79 5 6.57 5 6.52

5 6.47

5 6.28 5 6.26 5 6.13 5 6.08

5 6.01

5 5.79 5 5.77 5 5.69 5 5.62 5 5.57

5 5.46

5 5.35 5 5.31 5 5.06 5 4.97

1e-26 2e-20 1e-05 8e-27 3e-28

1e-20

2 3e-07 1.2 1.4

1e-20

1.5 2e-20 2e-24 9e-06 0.001

2e-20

2.3 2e-20 3e-19 1.5

1UD7- 114.0 16% A -

88.0 16% 2NVU-I -

4DWF- 68.0 15% A -

119.0 16% 1C3T-A -

2JWZ- 117.0 16% A -

Nedd8ala72arg) (77 residues with quality score 0.421). **NOTE: This template was deliberately discarded:** Its total score 6.47 is lower J than 30% of the best scoring X-ray template (534.80), and it newly covers only 0 target residues. It is therefore highly unlikely that this

94.0 16% 3DBR- -

75.0 17% 2IY1-D -

2BWE- 95.0 16% U -

2ASQ- 82.0 16% A -

82.0 16% 2BF8-B -

94.0 16% 3DBH-I -

2G4D- 82.0 16% D -

3GZN- 88.0 16% J -

101.0 17% 2JVC-A -

2WYQ- 61.0 15% A -

1YQB- 66.0 16% A -

90.0 16% 3DBL-I -

3KYC- 78.0 16% D -

1R4M- 88.0 16% L -

3DQV- 85.0 16% A -

1TGZ- 82.0 16% B -

template would yield a useful model.

Senp1 (Mutant) Full Length Sumo1 (83 residues with quality score 0.493). **NOTE: This template was deliberately discarded:** Its total score 6.28 is lower than 30% of the best scoring X-ray template (534.80), and it newly covers only 0 target residues. It is therefore highly unlikely that this template would yield a useful model.

The Crystal Structure Of The Complex Between The Uba And Ubl Domains Of Dsk2 (72 residues with quality score 0.420). **NOTE: This template was deliberately discarded:** Its total score 6.26 is lower than 30% of the best scoring X-ray template (534.80), and it newly covers only 0 target residues. It is therefore highly unlikely that this template would yield a useful model.

Solution Structure Of Sumo-1 In Complex With A Sumo-Binding Motif (Sbm) (77 residues with quality score 0.470). **NOTE: This template was deliberately discarded:** Its total score 6.13 is lower than 30% of the best scoring X-ray template (534.80), and it newly covers only 0 target residues. It is therefore highly unlikely that this template would yield a useful model.

Crystal Structure Of Sumo Modified Ubiquitin Conjugating Enzyme E2-25k (77 residues with quality score 0.466). **NOTE: This template was deliberately discarded:** Its total score 6.08 is lower than 30% of the best scoring X-ray template (534.80), and it newly covers only 0 target residues. It is therefore highly unlikely that this template would yield a useful model.

Structural Dissection Of A Gating Mechanism Preventing Misactivation Of Ubiquitin By Nedd8's E1 (Appbp1- Uba3arg190ala- Nedd8ala72arg) (86 residues with quality score 0.391). **NOTE: This template was deliberately discarded:** Its total score 6.01 is lower than 30% of the best scoring X-ray template (534.80), and it newly covers only 0 target residues. It is therefore highly unlikely that this template would yield a useful model.

Crystal Structure Of Human Senp1 Mutant (C603s) In Complex With Sumo-1 (78 residues with quality score 0.444). **NOTE: This template was deliberately discarded:** Its total score 5.79 is lower than 30% of the best scoring X-ray template (534.80), and it newly covers only 0 target residues. It is therefore highly unlikely that this template would yield a useful model.

Structure Of Nedd8-Activating Enzyme In Complex With Nedd8 And Mln4924 (79 residues with quality score 0.401). **NOTE: This template was deliberately discarded:** Its total score 5.77 is lower than 30% of the best scoring X-ray template (534.80), and it newly covers only 0 target residues. It is therefore highly unlikely that this template would yield a useful model.

Nmr Solution Structure Of Ubiquitin Like Protein (82 residues with quality score 0.340). **NOTE: This template was deliberately discarded:** Its total score 5.69 is lower than 30% of the best scoring X-ray template (534.80), and it newly covers only 0 target residues. It is therefore highly unlikely that this template would yield a useful model.

The Crystal Structure Of The Ubiquitin-Like (Ubl) Domain Of Hhr23a (Human Homologue A Of Rad23) (77 residues with quality score 0.595). **NOTE: This template was deliberately discarded:** Its total score 5.62 is lower than 30% of the best scoring X-ray template (534.80), and it newly covers only 0 target residues. It is therefore highly unlikely that this template would yield a useful model.

Human Ubiquilin 3 (88 residues with quality score 0.523). **NOTE: This template was deliberately discarded:** Its total score 5.57 is lower than 30% of the best scoring X-ray template (534.80), and it newly covers only 0 target residues. It is therefore highly unlikely that this template would yield a useful model.

Structural Dissection Of A Gating Mechanism Preventing Misactivation Of Ubiquitin By Nedd8's E1 (Appbp1- Uba3arg190wt- Nedd8ala72gln) (86 residues with quality score 0.371). **NOTE: This template was deliberately discarded:** Its total score 5.46 is lower than 30% of the best scoring X-ray template (534.80), and it newly covers only 0 target residues. It is therefore highly unlikely that this template would yield a useful model.

Human Sumo E1 Complex With A Sumo1-Amp Mimic (79 residues with quality score 0.431). **NOTE: This template was deliberately discarded:** Its total score 5.35 is lower than 30% of the best scoring X-ray template (534.80), and it newly covers only 0 target residues. It is therefore highly unlikely that this template would yield a useful model.

Appbp1-Uba3-Nedd8, An E1-Ubiquitin-Like Protein Complex (76 residues with quality score 0.369). **NOTE: This template was deliberately discarded:** Its total score 5.31 is lower than 30% of the best scoring X-ray template (534.80), and it newly covers only 0 target residues. It is therefore highly unlikely that this template would yield a useful model.

Structural Insights Into Nedd8 Activation Of Cullin-Ring Ligases: Conformational Control Of Conjugation (78 residues with quality score 0.364). **NOTE: This template was deliberately discarded:** Its total score 5.06 is lower than 30% of the best scoring X-ray template (534.80), and it newly covers only 0 target residues. It is therefore highly unlikely that this template would yield a useful model.

Structure Of Human Senp2 In Complex With Sumo-1 (78 residues with quality score 0.381). **NOTE: This template was deliberately discarded:** Its total score 4.97 is lower than 30% of the best scoring X-ray template (534.80), and it newly covers only 0 target residues. It is therefore highly unlikely that this template would yield a useful model.

Crystal Structure Of Ufd2 In Complex With The Ubiquitin-like (ubl) Domain Of Rad23 (72 residues with quality score 0.422). **NOTE: This**

3M62-

Crystal Structure Of Ufd2 In Complex With The Ubiquitin-like (ubl) Domain Of Rad23 (72 residues with quality score 0.422). **NOTE: This template was deliberately discarded:** Its total score 4.70 is lower than 30% of the best scoring X-ray template (534.80), and it newly covers only 0 target residues. It is therefore highly unlikely that this template would yield a useful model.

Otu Domain Of Crimean Congo Hemorrhagic Fever Virus In Complex With Isg15 (77 residues with quality score 0.570). **NOTE: This template was deliberately discarded:** Its total score 4.60 is lower than 30% of the best scoring X-ray template (534.80), and it newly covers only 0 target residues. It is therefore highly unlikely that this template would yield a useful model.

Crystal Structure Of Human Isg15 In Complex With Ns1 N-Terminal Region From Influenza Virus B, Northeast Structural Genomics Consortium Target Ids Hx6481, Hr2873, And Or2 (151 residues with quality score 0.544). **NOTE: This template was deliberately discarded:** Its total score 4.53 is lower than 30% of the best scoring X-ray template (534.80), and it newly covers only 0 target residues. It is therefore highly unlikely that this template would yield a useful model.

Solution Structure Of Ubiquitin-Like Domain Of Hhr23b Complexed With Ubiquitin-Interacting Motif Of Proteasome Subunit S5a (95 residues with quality score 0.360). **NOTE: This template was deliberately discarded:** Its total score 4.37 is lower than 30% of the best scoring X-ray template (534.80), and it newly covers only 0 target residues. It is therefore highly unlikely that this template would yield a useful model.

E2~ubiquitin-Hect (76 residues with resolution 3.100). **NOTE: This template was deliberately discarded:** Its total score 4.23 is lower than 30% of the best scoring X-ray template (534.80), and it newly covers only 0 target residues. It is therefore highly unlikely that this template would yield a useful model.

Complex Of Influenza Virus Protein With Host Anti-Viral Factor (153 residues with quality score 0.526). **NOTE: This template was deliberately discarded:** Its total score 4.19 is lower than 30% of the best scoring X-ray template (534.80), and it newly covers only 0 target residues. It is therefore highly unlikely that this template would yield a useful model.

Crystal Structure Of Human Isg15 In Complex With Ns1 N-Terminal Region From Influenza B Virus, Northeast Structural Genomics

Consortium Target Ids Hx6481, Hr2873, And Or2 (151 residues with quality score 0.502). **NOTE: This template was deliberately** D **discarded:** Its total score 4.18 is lower than 30% of the best scoring X-ray template (534.80), and it newly covers only 0 target residues. It

5 4.70 5 4.60

5 4.53

5 4.37

5 4.23 5 4.19

5 4.18

5 4.18 5 4.17 5 4.16 5 3.99

5 3.86 5 3.84

5 3.64 5 3.63 5 3.63 5 3.52 5 3.47 5 3.45 5 3.26 5 3.16

0.006 8e-05

8e-05

3e-08

2e-29 8e-05

8e-05

1.2 0.0002 3e-07 2e-29

1.4 2e-24

0.59 6e-09 8e-05 3e-08 1.5 0.0001 0.004 0.0003

73.0 15% 50.0 16%

53.0 16%

62.0 20%

115.0 16% 50.0 16%

53.0 16%

82.0 16% 53.0 13% 93.0 15% 110.0 16%

82.0 16% 90.0 11%

77.0 17% 72.0 16% 53.0 16% 68.0 17% 82.0 16% 49.0 16% 23.0 25% 53.0 13%

3M62- B -

3PHX- B -

3R66-D -

1UEL- - A

3JW0- X -

2PE6-B -

4LJO-A -

3M63- B -

4LCD- E -

2IY0-B -

1GJZ-B -

1A5R- A -

2DZI-A -

1Z2M- A -

1P1A-A -

1Z5S-B -

3PSE- B -

4AJY-B - 4LJP-A -

3RT3-B - 3SDL- -

is therefore highly unlikely that this template would yield a useful model. Non-Covalent Complex Between Human Sumo-1 And Human Ubc9 (74 residues with quality score 0.320). **NOTE: This template was**

**deliberately discarded:** Its total score 4.18 is lower than 30% of the best scoring X-ray template (534.80), and it newly covers only 0 target residues. It is therefore highly unlikely that this template would yield a useful model.

Structure Of An Active Ligase (hoip)/ubiquitin Transfer Complex (216 residues with resolution 1.560). **NOTE: This template was deliberately discarded:** Its total score 4.17 is lower than 30% of the best scoring X-ray template (534.80), and it newly covers only 0 target residues. It is therefore highly unlikely that this template would yield a useful model.

Crystal Structure Of Ufd2 In Complex With The Ubiquitin-like (ubl) Domain Of Dsk2 (72 residues with quality score 0.289). **NOTE: This template was deliberately discarded:** Its total score 4.16 is lower than 30% of the best scoring X-ray template (534.80), and it newly covers only 0 target residues. It is therefore highly unlikely that this template would yield a useful model.

Structure Of An Rsp5xubxsna3 Complex: Mechanism Of Ubiquitin Ligation And Lysine Prioritization By A Hect E3 (75 residues with resolution 3.100). **NOTE: This template was deliberately discarded:** Its total score 3.99 is lower than 30% of the best scoring X-ray template (534.80), and it newly covers only 0 target residues. It is therefore highly unlikely that this template would yield a useful model.

Senp1 (Mutant) Sumo1 Rangap (76 residues with quality score 0.296). **NOTE: This template was deliberately discarded:** Its total score 3.86 is lower than 30% of the best scoring X-ray template (534.80), and it newly covers only 0 target residues. It is therefore highly unlikely that this template would yield a useful model.

Solution Structure Of A Dimeric N-Terminal Fragment Of Human Ubiquitin (53 residues with quality score 0.389). **NOTE: This template was deliberately discarded:** Its total score 3.84 is lower than 30% of the best scoring X-ray template (534.80), and it newly covers only 0 target residues. It is therefore highly unlikely that this template would yield a useful model.

Structure Determination Of The Small Ubiquitin-Related Modifier Sumo-1, Nmr, 10 Structures (103 residues with quality score 0.282).

**NOTE: This template was deliberately discarded:** Its total score 3.64 is lower than 30% of the best scoring X-ray template (534.80), and it newly covers only 0 target residues. It is therefore highly unlikely that this template would yield a useful model.

2dziSOLUTION STRUCTURE OF THE N-Terminal Ubiquitin-Like Domain In Human Ubiquitin-Like Protein 4a (Gdx) (81 residues with quality score 0.317). **NOTE: This template was deliberately discarded:** Its total score 3.63 is lower than 30% of the best scoring X-ray template (534.80), and it newly covers only 0 target residues. It is therefore highly unlikely that this template would yield a useful model.

Crystal Structure Of Isg15, The Interferon-Induced Ubiquitin Cross Reactive Protein (152 residues with quality score 0.436). **NOTE: This template was deliberately discarded:** Its total score 3.63 is lower than 30% of the best scoring X-ray template (534.80), and it newly covers only 0 target residues. It is therefore highly unlikely that this template would yield a useful model.

Nmr Structure Of Ubiquitin-Like Domain Of Hhr23b (85 residues with quality score 0.309). **NOTE: This template was deliberately discarded:** Its total score 3.52 is lower than 30% of the best scoring X-ray template (534.80), and it newly covers only 0 target residues. It is therefore highly unlikely that this template would yield a useful model.

Crystal Structure Of A Complex Between Ubc9, Sumo-1, Rangap1 And Nup358RANBP2 (78 residues with quality score 0.266). **NOTE: This template was deliberately discarded:** Its total score 3.47 is lower than 30% of the best scoring X-ray template (534.80), and it newly covers only 0 target residues. It is therefore highly unlikely that this template would yield a useful model.

Structure Of A Viral Otu Domain Protease Bound To Interferon- Stimulated Gene 15 (Isg15) (150 residues with quality score 0.437). **NOTE: This template was deliberately discarded:** Its total score 3.45 is lower than 30% of the best scoring X-ray template (534.80), and it newly covers only 0 target residues. It is therefore highly unlikely that this template would yield a useful model.

Von Hippel-Lindau Protein-Elonginb-Elonginc Complex, Bound To Hif1- Alpha Peptide (105 residues with resolution 1.730). **NOTE: This template was deliberately discarded:** Its total score 3.26 is lower than 30% of the best scoring X-ray template (534.80), and it newly covers only 2 target residues. It is therefore highly unlikely that this template would yield a useful model.

Structure Of An Active Ligase (hoip-h889a)/ubiquitin Transfer Complex (215 residues with resolution 2.150). **NOTE: This template was deliberately discarded:** Its total score 3.16 is lower than 30% of the best scoring X-ray template (534.80), and it newly covers only 0 target residues. It is therefore highly unlikely that this template would yield a useful model.

Structure Of A Hif-1a-Pvhl-Elonginb-Elonginc Complex (106 residues with resolution 1.850). **NOTE: This template was deliberately discarded:** Its total score 3.08 is lower than 30% of the best scoring X-ray template (534.80), and it newly covers only 0 target residues. It is therefore highly unlikely that this template would yield a useful model.

Crystal Structure Of Socs-2 In Complex With Elongin-B And Elongin-C At 1.9a Resolution (103 residues with resolution 1.900). **NOTE: This template was deliberately discarded:** Its total score 3.01 is lower than 30% of the best scoring X-ray template (534.80), and it newly covers only 0 target residues. It is therefore highly unlikely that this template would yield a useful model.

Elimination Of The C-Cap In Ubiquitin Structure, Dynamics And Thermodynamic Consequences (82 residues with quality score 0.163). **NOTE: This template was deliberately discarded:** Its total score 2.99 is lower than 30% of the best scoring X-ray template (534.80), and it newly covers only 0 target residues. It is therefore highly unlikely that this template would yield a useful model.

Native Structure Of Lsd2 /aof1/kdm1b In Spacegroup Of C2221 At 2.13a (750 residues with quality score 0.456). **NOTE: This template was deliberately discarded:** Its total score 2.99 is lower than 30% of the best scoring X-ray template (534.80), and it newly covers only 0 target residues. It is therefore highly unlikely that this template would yield a useful model.

Crystal Structure Of Lsd2 (746 residues with quality score 0.339). **NOTE: This template was deliberately discarded:** Its total score 2.79 is lower than 30% of the best scoring X-ray template (534.80), and it newly covers only 0 target residues. It is therefore highly unlikely that this template would yield a useful model.

Native Structure Of Lsd2/aof1/kdm1b In Spacegroup Of I222 At 2.9a (750 residues with quality score 0.422). **NOTE: This template was deliberately discarded:** Its total score 2.77 is lower than 30% of the best scoring X-ray template (534.80), and it newly covers only 0 target residues. It is therefore highly unlikely that this template would yield a useful model.

Solution Structure Of The N-Terminal Ubiquitin-Like Domain In The Human Bat3 Protein (86 residues with quality score 0.255). **NOTE:**

**This template was deliberately discarded:** Its total score 2.69 is lower than 30% of the best scoring X-ray template (534.80), and it newly A covers only 0 target residues. It is therefore highly unlikely that this template would yield a useful model.

5 3.08 5 3.01 5 2.99 5 2.99 5 2.79 5 2.77

5 2.69 5 2.67 5 2.53 5 2.52

5 2.39 5 2.33

5 2.28

5 2.22

5 2.13 5 1.98 5 1.93

5 1.78

5 1.77 5 1.77

0.004 0.004 2e-24 0.23 1 0.23

3e-06 0.002 8e-05 0.23

6e-05 5e-06

0.004

9e-06

9e-06 4e-06 0.008

0.013

0.076 2e-29

1LM8- 23.0 25% B -

2C9W- 23.0 25% B -

1ZW7- 104.0 18% A -

4FWE- 24.0 27% A -

4GU1- 29.0 28% A -

4FWJ- 24.0 27% A -

1WX7- 69.0 18% A -

56.0 15% 2HJ8-A -

4FWF- 24.0 27% A -

1WH3- 54.0 17% A -

1WY8- 52.0 17% A -

62.0 17% 1WX9- -

24.0 21% 4BKS- -

60.0 16% 1P9D- - U

60.0 16% 1P98-A - 23.0 15% 2FAZ-A - 16.0 22% 2FNJ-B -

16.0 22% 4B9K-J -

38.0 12% 4LJQ-B -

108.0 20% 2K25-A - 4AWJ-

this template would yield a useful model.

High-Resolution Structure Of The Complex Of Hhr23a Ubiquitin-Like Domain And The C-Terminal Ubiquitin- Interacting Motif Of Proteasome Subunit S5a (78 residues with quality score 0.236). **NOTE: This template was deliberately discarded:** Its total score 2.22 is lower than 30% of the best scoring X-ray template (534.80), and it newly covers only 0 target residues. It is therefore highly unlikely that this template would yield a useful model.

High-Resolution Nmr Structure Of The Ubl-Domain Of Hhr23a (78 residues with quality score 0.226). **NOTE: This template was deliberately discarded:** Its total score 2.13 is lower than 30% of the best scoring X-ray template (534.80), and it newly covers only 0 target residues. It is therefore highly unlikely that this template would yield a useful model.

Ubiquitin-Like Domain Of Human Nuclear Zinc Finger Protein Np95 (77 residues with quality score 0.555). **NOTE: This template was deliberately discarded:** Its total score 1.98 is lower than 30% of the best scoring X-ray template (534.80), and it newly covers only 0 target residues. It is therefore highly unlikely that this template would yield a useful model.

Crystal Structure Of A B30.2SPRY DOMAIN-Containing Protein Gustavus In Complex With Elongin B And Elongin C (98 residues with resolution 1.800). **NOTE: This template was deliberately discarded:** Its total score 1.93 is lower than 30% of the best scoring X-ray template (534.80), and it newly covers only 0 target residues. It is therefore highly unlikely that this template would yield a useful model.

Pvhl-elob-eloc Complex_(2s,4r)-1-(3-amino-2-methylbenzoyl)-4- Hydroxy-n-(4-(4-methylthiazol-5-yl)benzyl) Pyrrolidine-2-carboxamide Bound (103 residues with quality score 0.516). **NOTE: This template was deliberately discarded:** Its total score 1.78 is lower than 30% of the best scoring X-ray template (534.80), and it newly covers only 0 target residues. It is therefore highly unlikely that this template would yield a useful model.

Crystal Structure Of The Catalytic Core Of E3 Ligase Hoip (192 residues with resolution 2.450). **NOTE: This template was deliberately discarded:** Its total score 1.77 is lower than 30% of the best scoring X-ray template (534.80), and it newly covers only 0 target residues. It is therefore highly unlikely that this template would yield a useful model.

Automated Nmr Structure Of The Ubb By Fapsy (103 residues with quality score 0.080). **NOTE: This template was deliberately discarded:** Its total score 1.77 is lower than 30% of the best scoring X-ray template (534.80), and it newly covers only 0 target residues. It is therefore highly unlikely that this template would yield a useful model.

Pvhl:elob:eloc Complex, In Complex With Capped Hydroxyproline (103 residues with quality score 0.495). **NOTE: This template was**

Solution Structure Of The N-Terminal Ubiquitin-Like Domain In The Human Ubiquilin 3 (Ubqln3) (106 residues with quality score 0.212). **NOTE: This template was deliberately discarded:** Its total score 2.67 is lower than 30% of the best scoring X-ray template (534.80), and it newly covers only 0 target residues. It is therefore highly unlikely that this template would yield a useful model.

Solution Nmr Structure Of The C-Terminal Domain Of The Interferon Alpha-Inducible Isg15 Protein From Homo Sapiens. Northeast Structural Genomics Target Hr2873b (75 residues with quality score 0.292). **NOTE: This template was deliberately discarded:** Its total score 2.53 is lower than 30% of the best scoring X-ray template (534.80), and it newly covers only 0 target residues. It is therefore highly unlikely that this template would yield a useful model.

Complex Structure Of Lsd2/aof1/kdm1b With H3k4 Mimic (739 residues with quality score 0.384). **NOTE: This template was deliberately discarded:** Its total score 2.52 is lower than 30% of the best scoring X-ray template (534.80), and it newly covers only 0 target residues. It is therefore highly unlikely that this template would yield a useful model.

Solution Structure Of C-Terminal Ubiquitin Like Domain Of Human 2'-5'-Oligoadenylate Synthetase-Like Protain (P59 Oasl) (87 residues

with quality score 0.257). **NOTE: This template was deliberately discarded:** Its total score 2.39 is lower than 30% of the best scoring X- ray template (534.80), and it newly covers only 0 target residues. It is therefore highly unlikely that this template would yield a useful model.

Solution Structure Of The N-Terminal Ubiquitin-Like Domain In Human Np95ICBP90-Like Ring Finger Protein (Nirf) (89 residues with quality score 0.261). **NOTE: This template was deliberately discarded:** Its total score 2.33 is lower than 30% of the best scoring X-ray template (534.80), and it newly covers only 0 target residues. It is therefore highly unlikely that this template would yield a useful model.

Von Hippel Lindau Protein:elonginb:elonginc Complex, In Complex With (2s,4r)-1-ethanoyl-n-[[4-(1,3-oxazol-5-yl)phenyl]methyl]-4-oxidanyl-

Pyrrolidine-2-carboxamide (103 residues with resolution 2.200). **NOTE: This template was deliberately discarded:** Its total score 2.28 is G lower than 30% of the best scoring X-ray template (534.80), and it newly covers only 0 target residues. It is therefore highly unlikely that

5 1.70 5 1.64 5 1.22 5 1.21 5 1.10 5 1.09 5 1.08 5 0.88 5 0.85

5 0.67

5 0.65 5 0.65 5 0.63 5 0.54 5 0.51 5 0.46 5 0.25 5 0.15 5 0.13 5 0.12 5 0.11 5 0.06

0.013 16.0 1.5 82.0 0.19 40.0 0.002 24.0 4e-07 13.0 1e-05 46.0 0.0007 67.0 0.008 16.0 0.076 21.0

2.2 22.0

2.6 7.0 2.2 24.0 0.005 17.0 0.53 23.0 0.001 21.0 0.13 22.0 0.75 6.0 0.13 29.0 0.38 18.0 0.72 9.0 0.26 5.0 2.3 6.0

4AWJ- 22% J -

16% 2IO2-B -

1V5O- 19% A -

1ZKH- 14% A -

4DBG- 15% A -

1TTN- 16% A -

15% 1J8C-A -

4JGH- 22% B -

17% 1WE6- - A

17% 1V5T-A -

18% 4E71-A -

1P0R- 14% A -

1WIM- 18% A -

1UH6- 14% A -

1WE7- 15% A -

16% 1V86-A -

4G1E- 11% B -

14% 2CT7-A -

3P1W- 2% A -

4EPO- 2% G -

4% 4KIK-A -

4B6W- 2% A -

Pvhl:elob:eloc Complex, In Complex With Capped Hydroxyproline (103 residues with quality score 0.495). **NOTE: This template was deliberately discarded:** Its total score 1.70 is lower than 30% of the best scoring X-ray template (534.80), and it newly covers only 0 target residues. It is therefore highly unlikely that this template would yield a useful model.

Crystal Structure Of Human Senp2 In Complex With Rangap1-sumo-1 (75 residues with quality score 0.126). **NOTE: This template was deliberately discarded:** Its total score 1.64 is lower than 30% of the best scoring X-ray template (534.80), and it newly covers only 0 target residues. It is therefore highly unlikely that this template would yield a useful model.

Solution Structure Of The Ubiquitin-Like Domain From Mouse Hypothetical 1700011n24rik Protein (102 residues with quality score 0.157). **NOTE: This template was deliberately discarded:** Its total score 1.22 is lower than 30% of the best scoring X-ray template (534.80), and it newly covers only 0 target residues. It is therefore highly unlikely that this template would yield a useful model.

Solution Structure Of A Human Ubiquitin-Like Domain In Sf3a1 (86 residues with quality score 0.365). **NOTE: This template was deliberately discarded:** Its total score 1.21 is lower than 30% of the best scoring X-ray template (534.80), and it newly covers only 0 target residues. It is therefore highly unlikely that this template would yield a useful model.

Crystal Structure Of Hoil-1l-Ubl Complexed With A Hoip-Uba Derivative (82 residues with quality score 0.544). **NOTE: This template was deliberately discarded:** Its total score 1.10 is lower than 30% of the best scoring X-ray template (534.80), and it newly covers only 0 target residues. It is therefore highly unlikely that this template would yield a useful model.

Solution Structure Of The Ubiquitin-Like Domain Of Human Dc- Ubp From Dendritic Cells (80 residues with quality score 0.145). **NOTE: This template was deliberately discarded:** Its total score 1.09 is lower than 30% of the best scoring X-ray template (534.80), and it newly covers only 0 target residues. It is therefore highly unlikely that this template would yield a useful model.

Solution Structure Of The Ubiquitin-Like Domain Of Hplic-2 (103 residues with quality score 0.104). **NOTE: This template was deliberately discarded:** Its total score 1.08 is lower than 30% of the best scoring X-ray template (534.80), and it newly covers only 0 target residues. It is therefore highly unlikely that this template would yield a useful model.

Structure Of The Socs2-elongin Bc Complex Bound To An N-terminal Fragment Of Cullin5 (104 residues with resolution 3.000). **NOTE: This template was deliberately discarded:** Its total score 0.88 is lower than 30% of the best scoring X-ray template (534.80), and it newly covers only 0 target residues. It is therefore highly unlikely that this template would yield a useful model.

Solution Structure Of Ubiquitin-Like Domain In Splicing Factor Aal91182 (111 residues with quality score 0.235). **NOTE: This template was deliberately discarded:** Its total score 0.85 is lower than 30% of the best scoring X-ray template (534.80), and it newly covers only 0 target residues. It is therefore highly unlikely that this template would yield a useful model.

Solution Structure Of The Ubiquitin-Like Domain From Mouse Hypothetical 8430435i17rik Protein (90 residues with quality score 0.177). **NOTE: This template was deliberately discarded:** Its total score 0.67 is lower than 30% of the best scoring X-ray template (534.80), and it newly covers only 0 target residues. It is therefore highly unlikely that this template would yield a useful model.

Crystal Structure Of The Rho Gtpase Binding Domain Of Plexin B2 (90 residues with quality score 0.515). **NOTE: This template was deliberately discarded:** Its total score 0.65 is lower than 30% of the best scoring X-ray template (534.80), and it newly covers only 0 target residues. It is therefore highly unlikely that this template would yield a useful model.

Solution Structure Of Ubl5 A Human Ubiquitin-Like Protein (73 residues with quality score 0.199). **NOTE: This template was deliberately discarded:** Its total score 0.65 is lower than 30% of the best scoring X-ray template (534.80), and it newly covers only 0 target residues. It is therefore highly unlikely that this template would yield a useful model.

Solution Structure Of The Ring Finger Domain Of The Human Ubcm4-Interacting Protein 4 (94 residues with quality score 0.202). **NOTE: This template was deliberately discarded:** Its total score 0.63 is lower than 30% of the best scoring X-ray template (534.80), and it newly covers only 0 target residues. It is therefore highly unlikely that this template would yield a useful model.

Solution Structure Of The Murine Ubiquitin-Like 5 Protein From Riken Cdna 0610031k06 (100 residues with quality score 0.170). **NOTE: This template was deliberately discarded:** Its total score 0.54 is lower than 30% of the best scoring X-ray template (534.80), and it newly covers only 0 target residues. It is therefore highly unlikely that this template would yield a useful model.

Solution Structure Of Ubiquitin-Like Domain In Sf3a120 (115 residues with quality score 0.164). **NOTE: This template was deliberately discarded:** Its total score 0.51 is lower than 30% of the best scoring X-ray template (534.80), and it newly covers only 0 target residues. It is therefore highly unlikely that this template would yield a useful model.

Solution Structure Of The Ubiquitin Domain From Mouse D7wsu128e Protein (95 residues with quality score 0.131). **NOTE: This template was deliberately discarded:** Its total score 0.46 is lower than 30% of the best scoring X-ray template (534.80), and it newly covers only 0 target residues. It is therefore highly unlikely that this template would yield a useful model.

Crystal Structure Of Integrin Alpha V Beta 3 With Coil-Coiled Tag (709 residues with quality score 0.359). **NOTE: This template was deliberately discarded:** Its total score 0.25 is lower than 30% of the best scoring X-ray template (534.80), and it newly covers only 0 target residues. It is therefore highly unlikely that this template would yield a useful model.

Solution Structure Of The Ibr Domain Of The Ring Finger Protein 31 Protein (86 residues with quality score 0.038). **NOTE: This template was deliberately discarded:** Its total score 0.15 is lower than 30% of the best scoring X-ray template (534.80), and it newly covers only 0 target residues. It is therefore highly unlikely that this template would yield a useful model.

Crystal Structure Of Rab Gdi From Plasmodium Falciparum, Pfl2060c (442 residues with quality score 0.497). **NOTE: This template was deliberately discarded:** Its total score 0.13 is lower than 30% of the best scoring X-ray template (534.80), and it newly covers only 0 target residues. It is therefore highly unlikely that this template would yield a useful model.

Crystal Structure Of Rnf8 Bound To The Ubc13MMS2 HETERODIMER (135 residues with quality score 0.615). **NOTE: This template was deliberately discarded:** Its total score 0.12 is lower than 30% of the best scoring X-ray template (534.80), and it newly covers only 0 target residues. It is therefore highly unlikely that this template would yield a useful model.

Human Ikb Kinase Beta (619 residues with quality score 0.553). **NOTE: This template was deliberately discarded:** Its total score 0.11 is lower than 30% of the best scoring X-ray template (534.80), and it newly covers only 0 target residues. It is therefore highly unlikely that this template would yield a useful model.

Architecture Of Trypanosoma Brucei Tubulin-binding Cofactor B (86 residues with quality score 0.491). **NOTE: This template was deliberately discarded:** Its total score 0.06 is lower than 30% of the best scoring X-ray template (534.80), and it newly covers only 0 target residues. It is therefore highly unlikely that this template would yield a useful model.

Crystal Structure Of Atbag1 In Complex With Hsp70 (177 residues with quality score 0.687). **NOTE: This template was deliberately**

5 0.04 5 0.02 5 0.02 5 0.02

5 0.01

5 0.01 5 0.01 5 0.01 5 0.00 5 -1.74 5 -4.19

4HWI- 1.9 3.0 2% B -

1QZE- 2e-06 6.0 2% A -

0.41 4.0 1% 4KIK-B - 0.84 8.0 3% 2CT2-A -

Crystal Structure Of Atbag1 In Complex With Hsp70 (177 residues with quality score 0.687). **NOTE: This template was deliberately discarded:** Its total score 0.04 is lower than 30% of the best scoring X-ray template (534.80), and it newly covers only 0 target residues. It is therefore highly unlikely that this template would yield a useful model.

Hhr23a Protein Structure Based On Residual Dipolar Coupling Data (214 residues with quality score 0.185). **NOTE: This template was deliberately discarded:** Its total score 0.02 is lower than 30% of the best scoring X-ray template (534.80), and it newly covers only 0 target residues. It is therefore highly unlikely that this template would yield a useful model.

Human Ikb Kinase Beta (650 residues with quality score 0.530). **NOTE: This template was deliberately discarded:** Its total score 0.02 is lower than 30% of the best scoring X-ray template (534.80), and it newly covers only 0 target residues. It is therefore highly unlikely that this template would yield a useful model.

Solution Structure Of The Ring Domain Of The Tripartite Motif Protein 32 (88 residues with quality score 0.104). **NOTE: This template was deliberately discarded:** Its total score 0.02 is lower than 30% of the best scoring X-ray template (534.80), and it newly covers only 0 target residues. It is therefore highly unlikely that this template would yield a useful model.

Insulin Receptor (Ir) Ectodomain In Complex With Fab's (807 residues with quality score 0.065). **NOTE: This template was deliberately discarded:** Its total score 0.01 is lower than 30% of the best scoring X-ray template (534.80), and it newly covers only 0 target residues. It

2.6 9.0 2% 2DTG- - E is therefore highly unlikely that this template would yield a useful model.

3LOH- 2.4 9.0 2% E -

3SHQ- 0.031 3.0 1% A -

1OQY- 2e-06 6.0 2% A -

4E3C- 0.35 3.0 0% C -

1WD2- 1e-09 144.0 12% A -

2JMO- 3e-09 435.0 17% A -

Structure Of The Insulin Receptor Ectodomain, Including Ct P (825 residues with quality score 0.059). **NOTE: This template was deliberately discarded:** Its total score 0.01 is lower than 30% of the best scoring X-ray template (534.80), and it newly covers only 0 target residues. It is therefore highly unlikely that this template would yield a useful model.

Crystal Structure Of Ublcp1 (299 residues with quality score 0.392). **NOTE: This template was deliberately discarded:** Its total score 0.01 is lower than 30% of the best scoring X-ray template (534.80), and it newly covers only 0 target residues. It is therefore highly unlikely that this template would yield a useful model.

Structure Of The Dna Repair Protein Hhr23a (363 residues with quality score 0.050). **NOTE: This template was deliberately discarded:** Its total score 0.01 is lower than 30% of the best scoring X-ray template (534.80), and it newly covers only 0 target residues. It is therefore highly unlikely that this template would yield a useful model.

X-ray Crystal Structure Of Human Ikk2 In An Active Conformation (624 residues with quality score 0.148). **NOTE: This template was deliberately discarded:** Its total score 0.00 is lower than 30% of the best scoring X-ray template (534.80), and it newly covers only 0 target residues. It is therefore highly unlikely that this template would yield a useful model.

Solution Structure Of The C-Terminal Ring From A Ring-Ibr- Ring (Triad) Motif (60 residues with quality score -0.104). **NOTE: This template was deliberately discarded:** Its total score -1.74 is lower than 30% of the best scoring X-ray template (534.80), and it newly covers only 0 target residues. It is therefore highly unlikely that this template would yield a useful model.

Ibr Domain Of Human Parkin (80 residues with quality score -0.056). **NOTE: This template was deliberately discarded:** Its total score -4.19 is lower than 30% of the best scoring X-ray template (534.80), and it newly covers only 0 target residues. It is therefore highly unlikely that this template would yield a useful model.

The 'Total score' in the second column is simply the coverage. This makes sure that good template structures are used even if the alignment score is lower. The quality score ranges from 0.000 (terrible) to 1.000 (perfect). The target coverage can be artificially low if the alignment scores so badly, that unaligned overhangs on both sides are more favorable.

**4. The secondary structure prediction**

To aid alignment correction and loop modeling, a secondary structure prediction for the target sequence had to be obtained. This was achieved by running PSI-BLAST to create a target sequence profile and feeding it to the PSI-Pred secondary structure prediction algorithm [Jones DT, J.Mol.Biol. **292**:195-202].

The resulting prediction is listed below, the lines 'PreHel', 'PreStr' and 'PreCoi' indicate the estimated probability for the three secondary structure classes helix, strand and coil.

Sequence: MIVFVRFNSSHGFPVEVDSDTSIFQLKEVVAKRQGVPADQLRVIFAGKELRNDWTVQNCDLDQQSIVHIVQRPWRKGQEMNATGGDDPRNAAGGCEREPQSLTRVDLSSSVLPGDSVGLAVILHTDSRKDSPPAGSPAGRSIYNSFYVYCKGPCQRVQPGKLRVQCSTCRQATLTLTQ SecStr : CEEEEECCCCCEEEEEECCCCCHHHHHHHHHHHHCCCCCCEEEEECCCCCCCCCCCCCCCCCCCCEEEEEECCCCCCCCCCCCCCCCCCCCCCCCCCCCCCCCCCCCCCCCCCCCCCCCEEEECCCCCCCCCCCCCCCCCCCCCCCCCCCCCCCCCCCCCCCCCCCCCCCCCCCCCCC PreHel : 0000000000000000000013999999998875000110000000000000111332000000000000000111010011000000001000000001223311000000000111111111100000000000000000000100111000000001122321344443232100 PreStr : 0999994100168999510000000000000000000002578984244210123333200001389998722110112211000000000000011111111221111121100122357764210000000000000123211223321001222210123443100001232221 PreCoi : 9000005899831000489986000000000224999987310015744668764445688998610001277778877778899999988899888898776567888888899877431135788899999999998876788775578988666667654446544455545678

**5. The target sequence profile**

To help align target and templates, a target sequence profile has been created from the following multiple sequence alignment, which is built from related UniRef90 sequences. This alignment has also been saved as hParkin__TRC2013_profile.ali. The color codes are: negative, positive, hydrophilic and hydrophobic.

**Target** : MIVFVRFNSSHGFPVEVDSDTSIFQLKEVVAKRQGVPADQLRVIFAGKELRNDWTVQNCDLDQQSIVHIVQRPWRKGQEMNATGGDDPRNAAGGCEREPQSLTRVDLSSSVLPGDSVGLAVILHTDSRKDSPPAGSPAGRSIYNSFYVYCKGPCQRVQPGKLRVQCSTCRQAT O60260 : MIVFVRFNSSHGFPVEVDSDTSIFQLKEVVAKRQGVPADQLRVIFAGKELRNDWTVQNCDLDQQSIVHIVQRPWRKGQEMNATGGDDPRNAAGGCEREPQSLTRVDLSSSVLPGDSVGLAVILHTDSRKDSPPAGSPAGRSIYNSFYVYCKGPCQRVQPGKLRVQCSTCRQAT B9VH11 : MIVFVRFNSSHGFPVEVDSDTSIFQLKEVVAKRQGVPTDQLRVIFAGKELRNDWTVQNCDLDQQSIVHIVQRPRRKGQEMNATGGDNARNTAGGCEREPQSLTRVDLSSSVLPGDSVGLAVILHTDSRNDSPPAGSP..RPIYNSFYVYCKGPCQRVQPGKLRVQCSTCRQAT UPI00017: ..VFVRFNSSHGFPVEVDSNTSIFQLKEAVAKRQGVPADQLRVIFAGKDLRNDLTVQSCDLDQQSIVHVVLRPQRKDQETNTPGGDKPQSA.GGSEREPESLTRVDLSSSILPTHSVGLAVILNSDCKNDVPPPGRPAGRSTYNSFYVYCKGPCQRVQPGKLRVRCSTCQQAT UPI0000E: ........................................................QHCDLDQQSIVHIVQRPWRKGQEMNATGGDDPRNAAGGCEREPQSLTRVDLSSSVLPGDSVGLAVILHTDSRKDSPPAGSPAGRSIYNSFYVYCKGPCQRVQPGKLRVQCSTCGQAT UPI0001D: ..MFVRFNSSHGFPVEVDSNTSIFQLKEVVAKRQGVPADQLHVIFAGKELRNDLTVQSCDLDQQSIVHVVLRPWREGQEREATRGDSPQKAMDGPEREPESLTRVDLSSSILPAHSVGLAVILNND.......AGRPAGRGTYNSFYVYCKGPCQRVQPGKLRVQCSTCRQAT Q1WDP3 : MIVFVRFNSSHGFPVEVDSDTSIFQLKEVVAKRQGVPADQLRVIFAGKELRNDLTVQRCDLDQQSIVHVVLRPQRNGQERGVAAGHRP........REPASLTRVDLSGSVLPGDAVGLAVILQDDSADGAAPAGRPADRPTNKSFYVYCKGPCQRVQPGKLRVRCSTCQQAT Q9WVS6 : MIVFVRFNSSYGFPVEVDSDTSILQLKEVVAKRQGVPADQLRVIFAGKELPNHLTVQNCDLEQQSIVHIVQRPRRRSHETNASGGDEPQSTSEGSIWESRSLTRVDLSSHTLPVDSVGLAVILDTDSKRDSEAARGP.....YNSFFIYCKGPCHKVQPGKLRVQCGTCKQAT F1MA69 : .LVFVRFNSSYGFPVEVDSDTSIFQLKEVVAKRQGVPADQLRVIFAGKELQNHLT..NCDLEQQSIVHIVQRPQRKSHETNASGGDKPQSTPEGSIWEPRSLTRVDLSSHILPADSVGLAVILDTDSKSDSEAARGPEAKPTYHSFFVYCKGPCHKVQPGKLRVQCGTCRQAT UPI00020: ..MFVRFNSSHGFPVEVEADTNIFQLKEVVAKRQGVPADQLHVIFAGKELRNDLTLQNCDLDQQSIVHVVQRAQRGDQKEAMSGQNDPGHSRGVIGREPESLTRVDLSSSILPAYSVGLAVILENEDKDDSPPAG...GTPTYNSFYVFCKGGCQGVQPGKLRVRCSTCKQAT

product of the BLAST alignment score, the WHAT_CHECK [Hooft et al., Nature **381**:272] quality score in the PDBFinder2 database and the target

UPI00020: ..MFVRFNSSHGFPVEVEADTNIFQLKEVVAKRQGVPADQLHVIFAGKELRNDLTLQNCDLDQQSIVHVVQRAQRGDQKEAMSGQNDPGHSRGVIGREPESLTRVDLSSSILPAYSVGLAVILENEDKDDSPPAG...GTPTYNSFYVFCKGGCQGVQPGKLRVRCSTCKQAT UPI0000E: MKVFVRFNSNHGFPVEVDSDTSIFQLKEVVARRQGVPADQLCVIFAGKELRNDWTVQSCDLDQQSIVHIVLRPRRKGPE.....GHSPRPAWGRSDREPESLTRVDLSSSMLPADSVGLAVILQDGEESGASSARRPAGRPTYNSFYVYCKGPCQGVQPGKLRVRCSTCQQAT UPI00019: ..VFVRFNSSHGFPVEVGSDSSILQLKEAVAQRQGVPADQLRVIFAGRELSNDLTLQNCDLAQQSIVHIVESPQKNSQDKEKTEYSCVGGVPKALKREPESLTRIDLSTSILPSVSAGLAVI...DPGKNMPFADSPASRASYNSFYVFCKNFCQAVKPGKLRVRCSVCKQGT F1NWU0 : ..VFVRFNSSHGFPVELGLDASILQLKEAVAQRQGVPADQLRVIFAGRELSNDLTLQNCDLVQQSIVHIVQ.......DKDETEDNHAGGILKTLERVPESLTRIDLSSSILPSLSAGLAVILDTKEPNISPPSEKSAGAASYNSFYVFCKNFCQAVKPGKLRVRCNECKQGT

D3JW61 : MKVFVRFNSNHGFPVEVDSDTSIFQLKEVVARRQGVPADQLCVIFAGKELRNDWTVQSCDLDQQSIVHIVLRPRRKG..........PRPAWGRSDRELESLTRVDLSSSVLPADSVGLAVILQDGEESGASSARRPAGRPTYNSFYVYCKGPCQGVQPGKLRVRCSTCQQAT D3JZW7 : MIVFVRFNSSHGFPVEVDSDTSIFQLKEVVAKRQGVPADQLRVIFAGKELRNDWTVQNCDLDQQSIVHIVQRPWRKGQEMNATGGDDPRNAAGGCEREPQSLTRVDLSSSVLPGDSVGLAVILHTDSRKDSPPAGSPAGRSIYNSFYVYCKGPCQRVQPGKLRVQCSTCRQAT Q561U2 : MIVFVRFNSSHGFPVELEQGASVSELKEAVGRLQGVQSDQLRVIFAGRELCNESTLQGCDLPEQSTVHVVLPP...............RRLGSG.......LTRLDLSSSRQTTASEGLAVILETEASRREDTAG.......HSSFYVFCKTVCKAIQPGKLRVRCKDCKQGT D3JVU6 : MIVFVRFNSSYGFPVEVDSDTSIFQLKEVVAKRQGVPADQLRVIFAGKELQNHLTVQNCDLEQQSIVHIVQRPQRKSHETNASGGDKPQSTPEGSIWEPRSLTRVDLSSHILPADSVGLAVILDTDSKSDSEAARGPA....YHSFFVYCKGPCHKVQPGKLRVQCGTCRQAT UPI00020: ..VFVRFNSSHGFPVEIDSDTSIFQLKEAVAKRQGVPADQLRVIFAGKELRNDLTVQSCDLPQQSIVHVIQTP.....................................................................YNSFYVFCKSFCQAVKPGKLRVHCRTCKQGT Q5VVX4 : MIVFVRFNSSHGFPVEVDSDTSIFQLKEVVAKRQGVPADQLRVIFAGKELRNDWTVQNCDLDQQSIVHIVQRPWRKGQEMNATGGDDPRNAAGGCEREPQSLTRVDLSSSVLPGDSVGLAVILHTDSRKDSPPAGSPAGRSIYNSFYVYCKGPCQRVQPGKLRVQCSTCRQAT Q5VVX3 : ................................................................................................................................................................................. Q19K47 : MIVFVRFNSSHGFPVEVDSDTSIFQLKEVVAKRQGVPADQLRVIFAGKELRNDLTVQRCDLDQQSIVHVVLRPQRNGQERGVAAGHRP........REPASLTRVDLSGSVLPGDAVGLAVILQDDSADGAAPAGRP.....NKSFYVYCKGPCQRVQPGKLRVRCSTCQQAT D3JW62 : MIVFVRFNSSYGFPVEVDSDTSIFQLKEVVAKRQGVPADQLRVIFAGKELQNHLTVQNCDLEQQSIVHIVQRPQRKSHETNASGGDKPQSTPEGSIWEPRSLTRVDLSSHILPADSVGLAVILDTDSKSDSEAARGPAAKPTYHSFFVYCKGPCHKVQPGKLRVQCGTCRQAT UPI00016: ..VFVRYNLGPEVVVELQEEATVAELKEVVGQQQGVQPDLLRVLFAGRELKSTSTLQGCDLPEQSTVH...................PEHLSQGEEENHDSLTRLDLSASRLPTTSSTLGVILERNDSEGSAGAKDHSVR....TFFVYCK..CKLVQPGKLRVRCRSCRQAT D2Y182 : MKVFVRFNSNHGFPVEVDSDTSIFQLKEVVARRQGVPADQLCVIFAGKELRNDWTVQSCDLDQQSIVHIVLRPRRKGPE.......SPRPAWGRSDRELESLTRVDLSSSVLPADSVGLAVILQDGEESGASSARRPAGRPTYNSFYVYCKGPCQGVQPGKLRVRCSTCQQAT UPI00017: ..VIVRYNLGPEVVVEVQEEATVAELKEVVARQQGVQPERLRVLFAGRELKSTSTLQDCDLPEQSTVH...........................EEDHDSLTRLDLSSSRLTTTTSGLAVIL.................SV...FFVYCK..CKSIQPGKLRVRCRSCRQTT Q5J4W3 : MIVFVRYNLGPEVVVELQEEATVAELKEVVGQQQGVQPDLLRVLFAGRELKSTSTLQGCDLPEQSTVH...................PEHLSQGEEENHDSLTRLDLSASRLPTTSSTLGVI.......................FFVYCK..CKLVQPGKLRVRCRSCRQAT C1BKS6 : MIVYVRFNSSHGFPLELEEGTSIAKLKETVGRLQGVQGEHLRVIFAGRELRSDSTLQGCDLPEQSTVHVVLPP...........................SLTRLDLSASRLPTTSTGLAVILETDEHRARELAEEHAGPRPHSSFYVYCKSVCGAIQPAKLRVRCSVCKQGT D3JZW4 : MIVFVRFNSSYGFPVEVDSDTSIFQLKEVVAKRQGVPADQLRVIFAGKELQNHLTVQNCDLEQQSIVHIVQRPQRKSHETNASGGDKPQSTPEGSIWEPRSLTRVDLSSHILPADSVGLAVILDTDSKSDSEAARGPAAKPTYHSFFVYCKGPCHKVQPGKLRVQCGTCRQAT E9G0L1 : ..VHIKAAGGKTFLIQMSRDWDVARIKKFIAPKVGLKVEDISIILAGKSLADDLLLEECDLGHNSILNAVKLKVIKKADTTSS..............................................................FYVYCEFPCKSIQSGKLRVRCSLCKAGA UPI00005: ...............................................................................................................................RSPETP........LHPSFYVYCKSHCRSVQPGKLRVCCQTCKDNA D2Y181 : MKVFVRFNSNHGFPVEVDSDTSIFQLKEVVARRQGVPADQLCVIFAGKELRNDWTVQSCDLDQQSIVHIVLRPRRKGPE........PRPAWGRSDRELESLTRVDLSSSVLPADSVGLAVILQDGEESGASSARRPAGRPTYNSFYVYCKGPCQGVQPGKLRVRCSTCQQAT C3Z502 : ..VMVRFNSNHSFLVTVHTSWTIARFKQEVGRTQGVPSGQIHILFAGRDLSDSLRIEDCQLGQQTVIHAI................DARSSA......PRSLS.........................................SYFVFCKRPCKAVRPGKLRVRCGTCRQTT Q7KTX7 : ..IYVKTNTGKTLTVNLEPQWDIKNVKELVAPQLGLQPDDLKIIFAGKELSDATTIEQCDLGQQSVLH.............................................................................FFVHC...CDKLCNGKLRVRCALCKGGA B0X8E8 : ..IYVKSNTGSTLSVDLEPHMDIKDVKEIVAPQLGLAPDELKIIFAGKELSDTITISECDLGQQSIIH....PTIPSPSKNFQSNGKRRLNSIISEESPEEPYPAGSSK....................................FFVYC...CEKVCTGKLRVRCGICRSGA Q17DC3 : ..IYVKTNTGNTLSVDLEPHMDIKDVKEIVAPQLGLAPGELKIIFAGKELSDTITISECDLGQQSIIHAV................................................................GSPARERRKAHFFVYC...CEKVCTGKLRVRCGICKSGA Q4S2K5 : ............................................................................................................................................................VQPERLRVLFAGRELKS E3WZE5 : ..VYVKTNTGSTLAVSLEPHMEIKEVKEMVAPQLGLEPAELKIIFAGRELSDTTTIRECDLGQQSIIHAV....RRGQQKQSLGG.................................................GAPAER.....FFVYC...CEKVCTGKLRVRCGICGSGA D3K2X0 : MIVFVRFNSSHGFPVEVDSDTSIFQLKEVVAKRQGVPADQLRVIFAGKELRNDWTVQNCDLDQQSIVHIVQRPWRKGQEMNATGGDDPRNAAGGCEREPQSLTRVDLSSSVLPGDSVGLAVILHTDSRKDSPPAGSPAGRSIYNSFYVYCKGPCQRVQPGKLRVQCSTCRQAT C3XVY8 : MQVFVRFNSHHSFPVDVDSSWSVLQLKEVLAARQQVPPAEIRIIFAGRELRDSFIIGECDLASHSIVHVV..........................QEPQSLTRVDL......................................FFVYCKQQCKDVQPGKLRVSCATCKQGT UPI00016: ..VFVRYNLGPEVVVELQEEATVAELKEVVGQQQGVQPDLLRVLFAGRELKSTSTLQGCDLPEQSTVH.....................HLSQGEEENHDSLTRLDLSASRLPTTSSTLGVIL......DSEGVGATAGGGGADTFFVYCK..CKLVQPGKLRVRCRSCRQAT B3RY20 : ..........................................................................................................................................GHNISSRFYVYCK..CGTVTPGKLRARCSKCKESS UPI00016: .................................................................................................................................................................................

**6. The initial homology models**

For each of the templates listed above, models were built. Either a single model if the alignment was certain, or a number of alternative models if the alignment was ambiguous.

**6.1. Homology model 1/6, based on template 4K95-L**

This model is a **monomer**, and based on the following profile-profile alignment: (To improve readability, only up to ten lines of the complete target multiple sequence alignment (MSA) shown above are also displayed here (upside down). The template MSA has been shrunk and focused

on structurally aligned PDB entries. The actual target-template alignment can be found in the middle at the line named 'Match'.)

D3K2X0 : MIVFVRFNSSHGFPVEVDSDTSIFQLKEVVAKRQGVPADQLRVIFAGKELRNDWTVQNCDLDQQSIVHIVQRPWRKGQEMNATGGDDPRNAAGGCEREPQSLTRVDLSSSVLPGDSVGLAVILHTDSRKDSPPAGSPAGRSIYNSFYVYCKGPCQRVQPGKLRVQCSTCRQAT B0X8E8 : ..IYVKSNTGSTLSVDLEPHMDIKDVKEIVAPQLGLAPDELKIIFAGKELSDTITISECDLGQQSIIH....PTIPSPSKNFQSNGKRRLNSIISEESPEEPYPAGSSK....................................FFVYC...CEKVCTGKLRVRCGICRSGA UPI00005: ...............................................................................................................................RSPETP........LHPSFYVYCKSHCRSVQPGKLRVCCQTCKDNA Q5J4W3 : MIVFVRYNLGPEVVVELQEEATVAELKEVVGQQQGVQPDLLRVLFAGRELKSTSTLQGCDLPEQSTVH...................PEHLSQGEEENHDSLTRLDLSASRLPTTSSTLGVI.......................FFVYCK..CKLVQPGKLRVRCRSCRQAT D3JW62 : MIVFVRFNSSYGFPVEVDSDTSIFQLKEVVAKRQGVPADQLRVIFAGKELQNHLTVQNCDLEQQSIVHIVQRPQRKSHETNASGGDKPQSTPEGSIWEPRSLTRVDLSSHILPADSVGLAVILDTDSKSDSEAARGPAAKPTYHSFFVYCKGPCHKVQPGKLRVQCGTCRQAT UPI00020: ..VFVRFNSSHGFPVEIDSDTSIFQLKEAVAKRQGVPADQLRVIFAGKELRNDLTVQSCDLPQQSIVHVIQTP.....................................................................YNSFYVFCKSFCQAVKPGKLRVHCRTCKQGT D3JW61 : MKVFVRFNSNHGFPVEVDSDTSIFQLKEVVARRQGVPADQLCVIFAGKELRNDWTVQSCDLDQQSIVHIVLRPRRKG..........PRPAWGRSDRELESLTRVDLSSSVLPADSVGLAVILQDGEESGASSARRPAGRPTYNSFYVYCKGPCQGVQPGKLRVRCSTCQQAT UPI00020: ..MFVRFNSSHGFPVEVEADTNIFQLKEVVAKRQGVPADQLHVIFAGKELRNDLTLQNCDLDQQSIVHVVQRAQRGDQKEAMSGQNDPGHSRGVIGREPESLTRVDLSSSILPAYSVGLAVILENEDKDDSPPAG...GTPTYNSFYVFCKGGCQGVQPGKLRVRCSTCKQAT UPI0001D: ..MFVRFNSSHGFPVEVDSNTSIFQLKEVVAKRQGVPADQLHVIFAGKELRNDLTVQSCDLDQQSIVHVVLRPWREGQEREATRGDSPQKAMDGPEREPESLTRVDLSSSILPAHSVGLAVILNND.......AGRPAGRGTYNSFYVYCKGPCQRVQPGKLRVQCSTCRQAT O60260 : MIVFVRFNSSHGFPVEVDSDTSIFQLKEVVAKRQGVPADQLRVIFAGKELRNDWTVQNCDLDQQSIVHIVQRPWRKGQEMNATGGDDPRNAAGGCEREPQSLTRVDLSSSVLPGDSVGLAVILHTDSRKDSPPAGSPAGRSIYNSFYVYCKGPCQRVQPGKLRVQCSTCRQAT SecStr : CEEEEECCCCCEEEEEECCCCCHHHHHHHHHHHHCCCCCCEEEEECCCCCCCCCCCCCCCCCCCCEEEEEECCCCCCCCCCCCCCCCCCCCCCCCCCCCCCCCCCCCCCCCCCCCCCCCEEEECCCCCCCCCCCCCCCCCCCCCCCCCCCCCCCCCCCCCCCCCCCCCCCCCCCCCC **Target** : MIVFVRFNSSHGFPVEVDSDTSIFQLKEVVAKRQGVPADQLRVIFAGKELRNDWTVQNCDLDQQSIVHIVQRPWRKGQEMNATGGDDPRNAAGGCEREPQSLTRVDLSSSVLPGDSVGLAVILHTDSRKDSPPAGSPAGRSIYNSFYVYCKGPCQRVQPGKLRVQCSTCRQAT **Match :** MIVFVRFNSS|GFPVEVDSDTSIFQLKEVVAKRQGVPADQLRVIFAGKEL:N TVQNCDL|QQSIVHIVQR Y:SF|VYCKGPC:|VQPGKLRVQC:TCRQATLTL: **Template**: MIVFVRFNSSYGFPVEVDSDTSIFQLKEVVAKRQGVPADQLRVIFAGKELQNHLTVQNCDLEQQSIVHIVQR.....................................................................TYHSFFVYCKGPCHKVQPGKLRVQCGTCRQAT SecStr : EEEEEECCCCCCEEEEETTTTCHHHHHHHHHHHHCCTTTEEEEEETTECCTTTTTTTTCCCCCCEEEEEEEC.....................................................................CCCCEEEEECCCCEEEEEEEEEEEECCCCCCEEECC UPI0000E: .......................................................................R.....................................................................IYNSFYVYCKGPCQRVQPGKLRVQCSTCGQAT D3JW61 : MKVFVRFNSNHGFPVEVDSDTSIFQLKEVVARRQGVPADQLCVIFAGKELRNDWTVQSCDLDQQSIVHIVGR.....................................................................TYNSFYVYCKGPCQGVQPGKLRVRCSTCQQAT UPI00017: ................................................................................................................................................TFFVYCKS.CKSIQPGKLRVRCRSCRQTT UPI00016: ..VFVRYNLGPEVVVELQEEATVAELKEVVGQQQGVQPDLLRVLFAGRELKSTSTLQGCDLPEQSTVHIIRL.....................................................................TTSSFFVYCKS.CKLVQPGKLRVRCRSCRQAT Q9JK66-6: .................................................................................................................................................................................

C3Z502 : ..VMVRFNSNHSFLVTVHTSWTIARFKQEVGRTQGVPSGQIHILFAGRDLSDSLRIEDCQLGQQTVIHAISR.....................................................................SLPSYFVFCKRPCKAVRPGKLRVRCGTCRQTT F5H8K6 : ................................................................................................................................................................................. C3XVY8 : MQVFVRFNSHHSFPVDVDSSWSVLQLKEVLAARQQVPPAEIRIIFAGRELRDSFIIGECDLASHSIVHVVRR.....................................................................TSHGFFVYCKQQCKDVQPGKLRVSCATCKQGT UPI00016: ................................................................................................................................................................................. A8VFH0 : ................................................................................................................................................................................. C1BKS6 : MIVYVRFNSSHGFPLELEEGTSIAKLKETVGRLQGVQGEHLRVIFAGRELRSDSTLQGCDLPEQSTVHVVLP.....................................................................PHSSFYVYCKSVCGAIQPAKLRVRCSVCKQGT Q17DC3 : ..IYVKTNTGNTLSVDLEPHMDIKDVKEIVAPQLGLAPGELKIIFAGKELSDTITISECDLGQQSIIHAVKA.....................................................................TIPKFFVYCS.QCEKVCTGKLRVRCGICKSGA B0X8E8 : ..IYVKSNTGSTLSVDLEPHMDIKDVKEIVAPQLGLAPDELKIIFAGKELSDTITISECDLGQQSIIHAVKR.....................................................................TIPSFFVYCS.QCEKVCTGKLRVRCGICRSGA

.

.

C Q C

.

.

. .

B0X8E8 : ..IYVKSNTGSTLSVDLEPHMDIKDVKEIVAPQLGLAPDELKIIFAGKELSDTITISECDLGQQSIIHAVKR.....................................................................TIPSFFVYCS.QCEKVCTGKLRVRCGICRSGA E7ER41 : .......................................................................R.....................................................................IYNSFYVYCKGPCQRVQPGKLRVQCSTCRQAT B4PE67 : ..IYVKTNTGKTLTVNLEPQWDIKNVKELVAPQLGLQPDDLKIIFAGKELSDATTIEQCDLGQQSILHAVQR.....................................................................KIQSFFVHCS.QCDKLCNGKLRVRCALCKGGA B4DGN4 : .................................................................................................................................................................................. F1MIV9 : .................................................................................................................................................................................. UPI0001C: .................................................................................................................................................................................. Q0IZN5 : .................................................................................................................................................................................. Q653S7 : ..................................................................................................................................................................................

**NOTE:** To save space, only 20 of 122 template profile alignments are shown above. The complete set of alignments can be found in the file hParkin__TRC2013_4k95-l_profile.ali.

In the alignment above, 374 of 465 target residues (80.4%) are aligned to template residues. Among these aligned residues, the sequence identity is 90.9% and the sequence similarity is 94.9% ('similar' means that the BLOSUM62 score is > 0). The following 3 loops had to be modeled:

Loop N-terminal anchor Loop sequence

1 2 3

EGECS QARWE HIVQR

C-terminal anchor RVDER TTKPC

AVFEASGTTTQAY AASKETIKK PWRKGQEMNATGGDDPRNAAGGCEREPQSLTRVDLSSSVLPGDSVGLAVILHTDSRKDSPPAGSPAGRS IYNSF

After the side-chains had been built, optimized and fine-tuned, all newly modeled parts were subjected to a combined steepest descent and simulated annealing minimization (i.e. the backbone atoms of aligned residues were kept fixed to avoid potential damage).

The resulting half-refined model has been saved as hParkin__TRC2013_4k95-l_refined050.yob and obtained the following quality Z-scores:

Check type Dihedrals Packing 1D Packing 3D Overall

Quality Z-score 1.382 -2.053 -1.872

-1.471

Comment Optimal Poor Satisfactory Satisfactory

Then a full unrestrained simulated annealing minimization was run for the entire model. The result has been saved as hParkin__TRC2013_4k95-l_refined100.yob, the corresponding Z-scores are listed below:

Check type Dihedrals Packing 1D Packing 3D Overall

Quality Z-score 1.413 -1.647 -1.992

-1.364

Comment Optimal Satisfactory Satisfactory Satisfactory

Since the overall quality Z-score improved to -1.364 during the minimization, this fully refined model has been accepted as the final one for this template and alignment. The final model for this template and alignment has been saved as hParkin__TRC2013_4k95-l.yob, and is shown below, together with a plot of its overall quality Z-score, shown per residue:

**6.2. Homology model 2/6, based on template 4I1F-A**

This model is a **monomer**, and based on the following profile-profile alignment:

(To improve readability, only up to ten lines of the complete target multiple sequence alignment (MSA) shown above are also displayed here (upside down). The template MSA has been shrunk and focused on structurally aligned PDB entries. The actual target-template alignment can be found in the middle at the line named 'Match'.)

D3K2X0 : B0X8E8 : UPI00005: Q5J4W3 : D3JW62 : UPI00020: D3JW61 : UPI00020: UPI0001D: O60260 : SecStr : **Target** : **Match : Template**: SecStr : O60260 : B9VH11 : Q1WDP3 : D3JVU6 : UPI00020: Q5J4W3 : D3JW61 : Q4S2K5 : C3XVY8 : E2AFR5 : Q19K47 : UPI00016: UPI00017: Q7Q591 : Q8K5C2 : E7ER41 : E3WZE5 : F1Q9C4 :

E3LYI0 : Q9XUS3 :

MIVFVRFNSSHGFPVEVDSDTSIFQLKEVVAKRQGVPADQLRVIFAGKELRNDWTVQNCDLDQQSIVHIVQRPWRKGQEMNATGGDDPRNAAGGCEREPQSLTRVDLSSSVLPGDSVGLAVILHTDSRKDSPPAGSPAGRSIYNSFYVYCKGPCQRVQPGKLRVQCSTCRQAT ..IYVKSNTGSTLSVDLEPHMDIKDVKEIVAPQLGLAPDELKIIFAGKELSDTITISECDLGQQSIIH....PTIPSPSKNFQSNGKRRLNSIISEESPEEPYPAGSSK....................................FFVYC...CEKVCTGKLRVRCGICRSGA ...............................................................................................................................RSPETP........LHPSFYVYCKSHCRSVQPGKLRVCCQTCKDNA MIVFVRYNLGPEVVVELQEEATVAELKEVVGQQQGVQPDLLRVLFAGRELKSTSTLQGCDLPEQSTVH...................PEHLSQGEEENHDSLTRLDLSASRLPTTSSTLGVI.......................FFVYCK..CKLVQPGKLRVRCRSCRQAT MIVFVRFNSSYGFPVEVDSDTSIFQLKEVVAKRQGVPADQLRVIFAGKELQNHLTVQNCDLEQQSIVHIVQRPQRKSHETNASGGDKPQSTPEGSIWEPRSLTRVDLSSHILPADSVGLAVILDTDSKSDSEAARGPAAKPTYHSFFVYCKGPCHKVQPGKLRVQCGTCRQAT ..VFVRFNSSHGFPVEIDSDTSIFQLKEAVAKRQGVPADQLRVIFAGKELRNDLTVQSCDLPQQSIVHVIQTP.....................................................................YNSFYVFCKSFCQAVKPGKLRVHCRTCKQGT MKVFVRFNSNHGFPVEVDSDTSIFQLKEVVARRQGVPADQLCVIFAGKELRNDWTVQSCDLDQQSIVHIVLRPRRKG..........PRPAWGRSDRELESLTRVDLSSSVLPADSVGLAVILQDGEESGASSARRPAGRPTYNSFYVYCKGPCQGVQPGKLRVRCSTCQQAT ..MFVRFNSSHGFPVEVEADTNIFQLKEVVAKRQGVPADQLHVIFAGKELRNDLTLQNCDLDQQSIVHVVQRAQRGDQKEAMSGQNDPGHSRGVIGREPESLTRVDLSSSILPAYSVGLAVILENEDKDDSPPAG...GTPTYNSFYVFCKGGCQGVQPGKLRVRCSTCKQAT ..MFVRFNSSHGFPVEVDSNTSIFQLKEVVAKRQGVPADQLHVIFAGKELRNDLTVQSCDLDQQSIVHVVLRPWREGQEREATRGDSPQKAMDGPEREPESLTRVDLSSSILPAHSVGLAVILNND.......AGRPAGRGTYNSFYVYCKGPCQRVQPGKLRVQCSTCRQAT MIVFVRFNSSHGFPVEVDSDTSIFQLKEVVAKRQGVPADQLRVIFAGKELRNDWTVQNCDLDQQSIVHIVQRPWRKGQEMNATGGDDPRNAAGGCEREPQSLTRVDLSSSVLPGDSVGLAVILHTDSRKDSPPAGSPAGRSIYNSFYVYCKGPCQRVQPGKLRVQCSTCRQAT CEEEEECCCCCEEEEEECCCCCHHHHHHHHHHHHCCCCCCEEEEECCCCCCCCCCCCCCCCCCCCEEEEEECCCCCCCCCCCCCCCCCCCCCCCCCCCCCCCCCCCCCCCCCCCCCCCCEEEECCCCCCCCCCCCCCCCCCCCCCCCCCCCCCCCCCCCCCCCCCCCCCCCCCCCCCC MIVFVRFNSSHGFPVEVDSDTSIFQLKEVVAKRQGVPADQLRVIFAGKELRNDWTVQNCDLDQQSIVHIVQRPWRKGQEMNATGGDDPRNAAGGCEREPQSLTRVDLSSSVLPGDSVGLAVILHTDSRKDSPPAGSPAGRSIYNSFYVYCKGPCQRVQPGKLRVQCSTCRQAT

SIYNSFYVYCKGPCQRVQPGKLRVQCSTCRQATLTLTQ ............................................................................................................................................SIYNSFYVYCKGPCQRVQPGKLRVQCSTCRQAT

............................................................................................................................................CCCCCEEEEECCCCCEEEEEEEEEEECCCCCCEEEECC ............................................................................................................................................SIYNSFYVYCKGPCQRVQPGKLRVQCSTCRQAT .............................................................................................................................................IYNSFYVYCKGPCQRVQPGKLRVQCSTCRQAT ................................................................................................................................................SFYVYCKGPCQRVQPGKLRVRCSTCQQAT ..............................................................................................................................................YHSFFVYCKGPCHKVQPGKLRVQCGTCRQAT ............................................................................................................................................STYNSFYVFCKSFCQAVKPGKLRVHCRTCKQGT ...............................................................................................................................................STFFVYCKS.CKLVQPGKLRVRCRSCRQAT ..............................................................................................................................................YNSFYVYCKGPCQGVQPGKLRVRCSTCQQAT ..........................................................................................................................................................QGVQPERLRVLFAGRELKS .................................................................................................................................................FFVYCKQQCKDVQPGKLRVSCATCKQGT .................................................................................................................................................FYVYCSAPCKNVTAGKLRVKCARCGSGA ................................................................................................................................................SFYVYCKGPCQRVQPGKLRVRCSTCQQAT .................................................................................................................................................................................. ................................................................................................................................................NFYVYCTSPCANTTVGKIRVRCFKCKSGA .................................................................................................................................................FFVYCS.QCEKVCTGKLRVRCGICGSGA .................................................................................................................................................................................. ............................................................................................................................................SIYNSFYVYCKGPCQRVQPGKLRVQCSTCRQAT .................................................................................................................................................FFVYCS.QCEKVCTGKLRVRCGICGSGA ..................................................................................................................................................................................

............................................................................................................................................SILGSFYVWCK.KCDDVQRGKLRVYCQNCASTS ............................................................................................................................................SILGSFYVWCKN.CDDVKRGKLRVYCQKCSSTS

**NOTE:** To save space, only 20 of 72 template profile alignments are shown above. The complete set of alignments can be found in the file hParkin__TRC2013_4i1f-a_profile.ali.

In the alignment above, 305 of 465 target residues (65.6%) are aligned to template residues. Among these aligned residues, the sequence identity is 99.7% and the sequence similarity is 99.7% ('similar' means that the BLOSUM62 score is > 0). The following 4 loops had to be modeled:

Loop N-terminal anchor 1 None 2 EGECS 3 GAHPT

4 RKVTC

Loop sequence DSRKDSPPAGSPAGR AVFEASGTTTQ SDKET

EGGNGL

C-terminal anchor SIYNS AYRVD SVALH

GCGFA

After the side-chains had been built, optimized and fine-tuned, all newly modeled parts were subjected to a combined steepest descent and simulated annealing minimization (i.e. the backbone atoms of aligned residues were kept fixed to avoid potential damage).

The resulting half-refined model has been saved as hParkin__TRC2013_4i1f-a_refined050.yob and obtained the following quality Z-scores:

Check type Dihedrals Packing 1D Packing 3D Overall

Quality Z-score 1.424 -0.756 -1.424

-0.750

Comment Optimal Good Satisfactory Good

Overall

-0.750

Good

Then a full unrestrained simulated annealing minimization was run for the entire model. The result has been saved as hParkin__TRC2013_4i1f-a_refined100.yob, the corresponding Z-scores are listed below:

Unfortunately the overall quality Z-score decreased to -0.917 during the minimization, making it likely that the model moved into the wrong direction, away from the real structure. It was therefore discarded, and the half-refined model has been accepted as the final one for this template and alignment instead.

**NOTE:** 125 N-terminal residues have been skipped entirely and are not part of the model, as requested by the 'TermExtension' and 'LoopLenMax' parameters. Since YASARA does not yet perform ab initio structure prediction, it would not make sense to append very long tails. If a 'tail' happens to contain an entire domain, it can usually be found in another model and joined manually, provided that the 'Templates' parameter has been set large enough to include also the other template. Nevertheless 15 terminal residues were modeled, but excluded from the Z-scores in both tables above, to minimize noise from terminal regions.

The final model for this template and alignment has been saved as hParkin__TRC2013_4i1f-a.yob, and is shown below, together with a plot of its overall quality Z-score, shown per residue:

**6.3. Homology model 3/6, based on template 4I1H-A**

This model is a **monomer**, and based on the following profile-profile alignment:

(To improve readability, only up to ten lines of the complete target multiple sequence alignment (MSA) shown above are also displayed here (upside down). The template MSA has been shrunk and focused on structurally aligned PDB entries. The actual target-template alignment can be found in the middle at the line named 'Match'.)

Check type

Quality Z-score

Comment

Dihedrals

1.547

Optimal

Packing 1D

-0.761

Good

Packing 3D

-1.817

Satisfactory

Overall

-0.917

Good

D3K2X0 : B0X8E8 : UPI00005: Q5J4W3 : D3JW62 : UPI00020: D3JW61 : UPI00020: UPI0001D: O60260 : SecStr : **Target** : **Match : Template**: SecStr :

MIVFVRFNSSHGFPVEVDSDTSIFQLKEVVAKRQGVPADQLRVIFAGKELRNDWTVQNCDLDQQSIVHIVQRPWRKGQEMNATGGDDPRNAAGGCEREPQSLTRVDLSSSVLPGDSVGLAVILHTDSRKDSPPAGSPAGRSIYNSFYVYCKGPCQRVQPGKLRVQCSTCRQAT ..IYVKSNTGSTLSVDLEPHMDIKDVKEIVAPQLGLAPDELKIIFAGKELSDTITISECDLGQQSIIH....PTIPSPSKNFQSNGKRRLNSIISEESPEEPYPAGSSK....................................FFVYC...CEKVCTGKLRVRCGICRSGA ...............................................................................................................................RSPETP........LHPSFYVYCKSHCRSVQPGKLRVCCQTCKDNA MIVFVRYNLGPEVVVELQEEATVAELKEVVGQQQGVQPDLLRVLFAGRELKSTSTLQGCDLPEQSTVH...................PEHLSQGEEENHDSLTRLDLSASRLPTTSSTLGVI.......................FFVYCK..CKLVQPGKLRVRCRSCRQAT MIVFVRFNSSYGFPVEVDSDTSIFQLKEVVAKRQGVPADQLRVIFAGKELQNHLTVQNCDLEQQSIVHIVQRPQRKSHETNASGGDKPQSTPEGSIWEPRSLTRVDLSSHILPADSVGLAVILDTDSKSDSEAARGPAAKPTYHSFFVYCKGPCHKVQPGKLRVQCGTCRQAT ..VFVRFNSSHGFPVEIDSDTSIFQLKEAVAKRQGVPADQLRVIFAGKELRNDLTVQSCDLPQQSIVHVIQTP.....................................................................YNSFYVFCKSFCQAVKPGKLRVHCRTCKQGT MKVFVRFNSNHGFPVEVDSDTSIFQLKEVVARRQGVPADQLCVIFAGKELRNDWTVQSCDLDQQSIVHIVLRPRRKG..........PRPAWGRSDRELESLTRVDLSSSVLPADSVGLAVILQDGEESGASSARRPAGRPTYNSFYVYCKGPCQGVQPGKLRVRCSTCQQAT ..MFVRFNSSHGFPVEVEADTNIFQLKEVVAKRQGVPADQLHVIFAGKELRNDLTLQNCDLDQQSIVHVVQRAQRGDQKEAMSGQNDPGHSRGVIGREPESLTRVDLSSSILPAYSVGLAVILENEDKDDSPPAG...GTPTYNSFYVFCKGGCQGVQPGKLRVRCSTCKQAT ..MFVRFNSSHGFPVEVDSNTSIFQLKEVVAKRQGVPADQLHVIFAGKELRNDLTVQSCDLDQQSIVHVVLRPWREGQEREATRGDSPQKAMDGPEREPESLTRVDLSSSILPAHSVGLAVILNND.......AGRPAGRGTYNSFYVYCKGPCQRVQPGKLRVQCSTCRQAT MIVFVRFNSSHGFPVEVDSDTSIFQLKEVVAKRQGVPADQLRVIFAGKELRNDWTVQNCDLDQQSIVHIVQRPWRKGQEMNATGGDDPRNAAGGCEREPQSLTRVDLSSSVLPGDSVGLAVILHTDSRKDSPPAGSPAGRSIYNSFYVYCKGPCQRVQPGKLRVQCSTCRQAT CEEEEECCCCCEEEEEECCCCCHHHHHHHHHHHHCCCCCCEEEEECCCCCCCCCCCCCCCCCCCCEEEEEECCCCCCCCCCCCCCCCCCCCCCCCCCCCCCCCCCCCCCCCCCCCCCCCEEEECCCCCCCCCCCCCCCCCCCCCCCCCCCCCCCCCCCCCCCCCCCCCCCCCCCCCC MIVFVRFNSSHGFPVEVDSDTSIFQLKEVVAKRQGVPADQLRVIFAGKELRNDWTVQNCDLDQQSIVHIVQRPWRKGQEMNATGGDDPRNAAGGCEREPQSLTRVDLSSSVLPGDSVGLAVILHTDSRKDSPPAGSPAGRSIYNSFYVYCKGPCQRVQPGKLRVQCSTCRQAT

SIYNSFYVYCKGPCQRVQPGKLRVQCSTCRQATLTLT ............................................................................................................................................SIYNSFYVYCKGPCQRVQPGKLRVQCSTCRQAT

............................................................................................................................................CCCCCEEEEECCCCEEEEEEEEEEEECCCCCCEEEEC

C Q C

SecStr : ............................................................................................................................................CCCCCEEEEECCCCEEEEEEEEEEEECCCCCCEEEECC O60260 : ............................................................................................................................................SIYNSFYVYCKGPCQRVQPGKLRVQCSTCRQAT B9VH11 : .............................................................................................................................................IYNSFYVYCKGPCQRVQPGKLRVQCSTCRQAT Q1WDP3 : ................................................................................................................................................SFYVYCKGPCQRVQPGKLRVRCSTCQQAT UPI00020: ..............................................................................................................................................YNSFYVFCKGGCQGVQPGKLRVRCSTCKQAT D3JVU6 : ..............................................................................................................................................YHSFFVYCKGPCHKVQPGKLRVQCGTCRQAT Q5J4W3 : ...............................................................................................................................................STFFVYCKS.CKLVQPGKLRVRCRSCRQAT UPI00005: ............................................................................................................................................SLHPSFYVYCKSHCRSVQPGKLRVCCQTCKDNA Q19K47 : ................................................................................................................................................SFYVYCKGPCQRVQPGKLRVRCSTCQQAT C3XVY8 : .................................................................................................................................................FFVYCKQQCKDVQPGKLRVSCATCKQGT UPI00016: ...............................................................................................................................................DTFFVYCKS.CKLVQPGKLRVRCRSCRQAT Q7KTX7 : .................................................................................................................................................FFVHCS.QCDKLCNGKLRVRCALCKGGA E7ER41 : ............................................................................................................................................SIYNSFYVYCKGPCQRVQPGKLRVQCSTCRQAT Q7Q591 : .................................................................................................................................................FFVYCS.QCEKVCTGKLRVRCGICGSGA UPI00017: ................................................................................................................................................NFYVYCTSPCANTTVGKIRVRCFKCKSGA C1BKS6 : ..............................................................................................................................................HSSFYVYCKSVCGAIQPAKLRVRCSVCKQGT Q8K5C2 : .................................................................................................................................................................................. UPI00005: .................................................................................................................................................................................. Q17DC5 : .................................................................................................................................................................................. Q9XUS3 : ............................................................................................................................................SILGSFYVWCKN.CDDVKRGKLRVYCQKCSSTS A8XSA1 : ............................................................................................................................................SILGSFYVWCKS.CDDVRRGKLRVYCQNCESTS

**NOTE:** To save space, only 20 of 74 template profile alignments are shown above. The complete set of alignments can be found in the file hParkin__TRC2013_4i1h-a_profile.ali.

In the alignment above, 306 of 465 target residues (65.8%) are aligned to template residues. Among these aligned residues, the sequence identity is 100.0% and the sequence similarity is 100.0% ('similar' means that the BLOSUM62 score is > 0). The following 3 loops had to be modeled:

Loop N-terminal anchor Loop sequence C-terminal anchor

1 2 3

None EGECS RKVTC

DSRKDSPPAGSPAGR SIYNS AVFEASGTTTQAYR VDERA EGGNGL GCGFA

After the side-chains had been built, optimized and fine-tuned, all newly modeled parts were subjected to a combined steepest descent and simulated annealing minimization (i.e. the backbone atoms of aligned residues were kept fixed to avoid potential damage).

The resulting half-refined model has been saved as hParkin__TRC2013_4i1h-a_refined050.yob and obtained the following quality Z-scores:

Check type Dihedrals Packing 1D Packing 3D Overall

Quality Z-score 1.291 -0.990 -1.447

-0.872

Comment Optimal Good Satisfactory Good

Then a full unrestrained simulated annealing minimization was run for the entire model. The result has been saved as hParkin__TRC2013_4i1h-a_refined100.yob, the corresponding Z-scores are listed below:

Comment Optimal Good Satisfactory Satisfactory

Unfortunately and the half-refined model has been accepted as the final one for this template and alignment instead.

**NOTE:** 125 N-terminal residues have been skipped entirely and are not part of the model, as requested by the 'TermExtension' and 'LoopLenMax' parameters. Since YASARA does not yet perform ab initio structure prediction, it would not make sense to append very long tails. If a 'tail' happens to contain an entire domain, it can usually be found in another model and joined manually, provided that the 'Templates' parameter has been set large enough to include also the other template. Nevertheless 15 terminal residues were modeled, but excluded from the Z-scores in both tables above, to minimize noise from terminal regions.

The final model for this template and alignment has been saved as hParkin__TRC2013_4i1h-a.yob, and is shown below, together with a plot of its overall quality Z-score, shown per residue:

Check type Dihedrals Packing 1D Packing 3D Overall

Quality Z-score 1.437 -0.995 -1.902

-1.064

the overall quality Z-score decreased to -1.064 during the minimization, making it likely that the model moved into the wrong direction, away from the real structure. It was therefore discarded,

**6.4. Homology model 4/6, based on template 4BM9-A, alignment variant 01**

This model is a **monomer**, and based on the following profile-profile alignment:

(To improve readability, only up to ten lines of the complete target multiple sequence alignment (MSA) shown above are also displayed here (upside down). The template MSA has been shrunk and focused on structurally aligned PDB entries. The actual target-template alignment can be found in the middle at the line named 'Match'.)

D3K2X0 : B0X8E8 : UPI00005: Q5J4W3 : D3JW62 : UPI00020: D3JW61 : UPI00020: UPI0001D: O60260 : SecStr : **Target** : **Match : Template**: SecStr : O60260 : B9VH11 : Q1WDP3 : UPI00017: D3JVU6 : UPI00020: D3JW61 : Q9JK66-6: Q4S2K5 : E9G0L1 : UPI00016: B3RY20 : E2AFR5 : Q17DC3 : UPI00017: E2BWM9 : E9JCM0 : UPI00015: UPI0001C: B4H5X6 :

MIVFVRFNSSHGFPVEVDSDTSIFQLKEVVAKRQGVPADQLRVIFAGKELRNDWTVQNCDLDQQSIVHIVQRPWRKGQEMNATGGDDPRNAAGGCEREPQSLTRVDLSSSVLPGDSVGLAVILHTDSRKDSPPAGSPAGRSIYNSFYVYCKGPCQRVQPGKLRVQCSTCRQAT ..IYVKSNTGSTLSVDLEPHMDIKDVKEIVAPQLGLAPDELKIIFAGKELSDTITISECDLGQQSIIH....PTIPSPSKNFQSNGKRRLNSIISEESPEEPYPAGSSK....................................FFVYC...CEKVCTGKLRVRCGICRSGA ...............................................................................................................................RSPETP........LHPSFYVYCKSHCRSVQPGKLRVCCQTCKDNA MIVFVRYNLGPEVVVELQEEATVAELKEVVGQQQGVQPDLLRVLFAGRELKSTSTLQGCDLPEQSTVH...................PEHLSQGEEENHDSLTRLDLSASRLPTTSSTLGVI.......................FFVYCK..CKLVQPGKLRVRCRSCRQAT MIVFVRFNSSYGFPVEVDSDTSIFQLKEVVAKRQGVPADQLRVIFAGKELQNHLTVQNCDLEQQSIVHIVQRPQRKSHETNASGGDKPQSTPEGSIWEPRSLTRVDLSSHILPADSVGLAVILDTDSKSDSEAARGPAAKPTYHSFFVYCKGPCHKVQPGKLRVQCGTCRQAT ..VFVRFNSSHGFPVEIDSDTSIFQLKEAVAKRQGVPADQLRVIFAGKELRNDLTVQSCDLPQQSIVHVIQTP.....................................................................YNSFYVFCKSFCQAVKPGKLRVHCRTCKQGT MKVFVRFNSNHGFPVEVDSDTSIFQLKEVVARRQGVPADQLCVIFAGKELRNDWTVQSCDLDQQSIVHIVLRPRRKG..........PRPAWGRSDRELESLTRVDLSSSVLPADSVGLAVILQDGEESGASSARRPAGRPTYNSFYVYCKGPCQGVQPGKLRVRCSTCQQAT ..MFVRFNSSHGFPVEVEADTNIFQLKEVVAKRQGVPADQLHVIFAGKELRNDLTLQNCDLDQQSIVHVVQRAQRGDQKEAMSGQNDPGHSRGVIGREPESLTRVDLSSSILPAYSVGLAVILENEDKDDSPPAG...GTPTYNSFYVFCKGGCQGVQPGKLRVRCSTCKQAT ..MFVRFNSSHGFPVEVDSNTSIFQLKEVVAKRQGVPADQLHVIFAGKELRNDLTVQSCDLDQQSIVHVVLRPWREGQEREATRGDSPQKAMDGPEREPESLTRVDLSSSILPAHSVGLAVILNND.......AGRPAGRGTYNSFYVYCKGPCQRVQPGKLRVQCSTCRQAT MIVFVRFNSSHGFPVEVDSDTSIFQLKEVVAKRQGVPADQLRVIFAGKELRNDWTVQNCDLDQQSIVHIVQRPWRKGQEMNATGGDDPRNAAGGCEREPQSLTRVDLSSSVLPGDSVGLAVILHTDSRKDSPPAGSPAGRSIYNSFYVYCKGPCQRVQPGKLRVQCSTCRQAT CEEEEECCCCCEEEEEECCCCCHHHHHHHHHHHHCCCCCCEEEEECCCCCCCCCCCCCCCCCCCCEEEEEECCCCCCCCCCCCCCCCCCCCCCCCCCCCCCCCCCCCCCCCCCCCCCCCEEEECCCCCCCCCCCCCCCCCCCCCCCCCCCCCCCCCCCCCCCCCCCCCCCCCCCCCC MIVFVRFNSSHGFPVEVDSDTSIFQLKEVVAKRQGVPADQLRVIFAGKELRNDWTVQNCDLDQQSIVHIVQRPWRKGQEMNATGGDDPRNAAGGCEREPQSLTRVDLSSSVLPGDSVGLAVILHTDSRKDSPPAGSPAGRSIYNSFYVYCKGPCQRVQPGKLRVQCSTCRQAT

IYNSFYVYCKGPCQRVQPGKLRVQCSTCRQATLTLT .............................................................................................................................................IYNSFYVYCKGPCQRVQPGKLRVQCSTCRQAT

.............................................................................................................................................CCCCEEEEECCCCEEEEEEEEEEEECCCCCCEEEEC .............................................................................................................................................IYNSFYVYCKGPCQRVQPGKLRVQCSTCRQAT .............................................................................................................................................IYNSFYVYCKGPCQRVQPGKLRVQCSTCRQAT ................................................................................................................................................SFYVYCKGPCQRVQPGKLRVRCSTCQQAT ..............................................................................................................................................YNSFYVYCKGPCQRVQPGKLRVRCSTCQQAT ..............................................................................................................................................YHSFFVYCKGPCHKVQPGKLRVQCGTCRQAT ..............................................................................................................................................YNSFYVFCKSFCQAVKPGKLRVHCRTCKQGT ..............................................................................................................................................YNSFYVYCKGPCQGVQPGKLRVRCSTCQQAT ................................................................................................................................................................................. ..........................................................................................................................................................QGVQPERLRVLFAGRELKS .................................................................................................................................................FYVYCEFPCKSIQSGKLRVRCSLCKAGA ................................................................................................................................................................................. .............................................................................................................................................ISSRFYVYCKR.CGTVTPGKLRARCSKCKESS .................................................................................................................................................FYVYCSAPCKNVTAGKLRVKCARCGSGA .................................................................................................................................................FFVYCS.QCEKVCTGKLRVRCGICKSGA ................................................................................................................................................NFYVYCTSPCANTTVGKIRVRCFKCKSGA .................................................................................................................................................FYIYCPGPCKIVTAGKLRVKCAKCNSGA .................................................................................................................................................FYVYCSAPCKDVTAGKLRVKCAKCGSGA .................................................................................................................................................FYVYCSYPCKSVEFGKLRVRCQDCGGGA .................................................................................................................................................FYVFCK.MCADVRLGKLRVCCQQCQAAT .................................................................................................................................................FFVHC.GQCDKLCNGKLRVRCSLCKGGA

**NOTE:** To save space, only 20 of 70 template profile alignments are shown above. The complete set of alignments can be found in the file hParkin__TRC2013_4bm9-a_profile.ali.

C Q C

. .

In the alignment above, 301 of 465 target residues (64.7%) are aligned to template residues. Among these aligned residues, the sequence identity is 100.0% and the sequence similarity is 100.0% ('similar' means that the BLOSUM62 score is > 0). The following 4 loops had to be modeled:

Loop N-terminal anchor 1 None 2 QARWE 3 AVFEA

4 KVTCE

Loop sequence SRKDSPPAGSPAGRS AASKETIK SGTTTQA GGNGLGCG

C-terminal anchor IYNSF KTTKP YRVDE

FAFCR

After the side-chains had been built, optimized and fine-tuned, all newly modeled parts were subjected to a combined steepest descent and simulated annealing minimization (i.e. the backbone atoms of aligned residues were kept fixed to avoid potential damage).

The resulting half-refined model has been saved as hParkin__TRC2013_4bm9-a01_refined050.yob and obtained the following quality Z-scores:

Check type Dihedrals Packing 1D Packing 3D Overall

Quality Z-score 0.964 -1.349 -1.787

-1.217

Comment Optimal Satisfactory Satisfactory Satisfactory

Then a full unrestrained simulated annealing minimization was run for the entire model. The result has been saved as hParkin__TRC2013_4bm9-a01_refined100.yob, the corresponding Z-scores are listed below:

Comment Optimal Satisfactory Poor Satisfactory

Unfortunately and the half-refined model has been accepted as the final one for this template and alignment instead.

**NOTE:** 126 N-terminal residues have been skipped entirely and are not part of the model, as requested by the 'TermExtension' and 'LoopLenMax' parameters. Since YASARA does not yet perform ab initio structure prediction, it would not make sense to append very long tails. If a 'tail' happens to contain an entire domain, it can usually be found in another model and joined manually, provided that the 'Templates' parameter has been set large enough to include also the other template. Nevertheless 15 terminal residues were modeled, but excluded from the Z-scores in both tables above, to minimize noise from terminal regions.

The final model for this template and alignment has been saved as hParkin__TRC2013_4bm9-a01.yob, and is shown below, together with a plot of its overall quality Z-score, shown per residue:

Check type Dihedrals Packing 1D Packing 3D Overall

Quality Z-score 1.114 -1.127 -2.041

-1.227

the overall quality Z-score decreased to -1.227 during the minimization, making it likely that the model moved into the wrong direction, away from the real structure. It was therefore discarded,

**6.5. Homology model 5/6, based on template 4BM9-A, alignment variant 02**

This model is a **monomer**, and based on the following profile-profile alignment:

(To improve readability, only up to ten lines of the complete target multiple sequence alignment (MSA) shown above are also displayed here (upside down). The template MSA has been shrunk and focused on structurally aligned PDB entries. The actual target-template alignment can be found in the middle at the line named 'Match'.)

D3K2X0 : B0X8E8 : UPI00005: Q5J4W3 : D3JW62 : UPI00020: D3JW61 : UPI00020: UPI0001D: O60260 : SecStr : **Target** : **Match : Template**: SecStr : O60260 : B9VH11 : Q1WDP3 : UPI00017: D3JVU6 : UPI00020: D3JW61 : Q9JK66-6: Q4S2K5 : E9G0L1 : UPI00016: B3RY20 : E2AFR5 : Q17DC3 : UPI00017: E2BWM9 : E9JCM0 : UPI00015: UPI0001C: B4H5X6 :

MIVFVRFNSSHGFPVEVDSDTSIFQLKEVVAKRQGVPADQLRVIFAGKELRNDWTVQNCDLDQQSIVHIVQRPWRKGQEMNATGGDDPRNAAGGCEREPQSLTRVDLSSSVLPGDSVGLAVILHTDSRKDSPPAGSPAGRSIYNSFYVYCKGPCQRVQPGKLRVQCSTCRQAT ..IYVKSNTGSTLSVDLEPHMDIKDVKEIVAPQLGLAPDELKIIFAGKELSDTITISECDLGQQSIIH....PTIPSPSKNFQSNGKRRLNSIISEESPEEPYPAGSSK....................................FFVYC...CEKVCTGKLRVRCGICRSGA ...............................................................................................................................RSPETP........LHPSFYVYCKSHCRSVQPGKLRVCCQTCKDNA MIVFVRYNLGPEVVVELQEEATVAELKEVVGQQQGVQPDLLRVLFAGRELKSTSTLQGCDLPEQSTVH...................PEHLSQGEEENHDSLTRLDLSASRLPTTSSTLGVI.......................FFVYCK..CKLVQPGKLRVRCRSCRQAT MIVFVRFNSSYGFPVEVDSDTSIFQLKEVVAKRQGVPADQLRVIFAGKELQNHLTVQNCDLEQQSIVHIVQRPQRKSHETNASGGDKPQSTPEGSIWEPRSLTRVDLSSHILPADSVGLAVILDTDSKSDSEAARGPAAKPTYHSFFVYCKGPCHKVQPGKLRVQCGTCRQAT ..VFVRFNSSHGFPVEIDSDTSIFQLKEAVAKRQGVPADQLRVIFAGKELRNDLTVQSCDLPQQSIVHVIQTP.....................................................................YNSFYVFCKSFCQAVKPGKLRVHCRTCKQGT MKVFVRFNSNHGFPVEVDSDTSIFQLKEVVARRQGVPADQLCVIFAGKELRNDWTVQSCDLDQQSIVHIVLRPRRKG..........PRPAWGRSDRELESLTRVDLSSSVLPADSVGLAVILQDGEESGASSARRPAGRPTYNSFYVYCKGPCQGVQPGKLRVRCSTCQQAT ..MFVRFNSSHGFPVEVEADTNIFQLKEVVAKRQGVPADQLHVIFAGKELRNDLTLQNCDLDQQSIVHVVQRAQRGDQKEAMSGQNDPGHSRGVIGREPESLTRVDLSSSILPAYSVGLAVILENEDKDDSPPAG...GTPTYNSFYVFCKGGCQGVQPGKLRVRCSTCKQAT ..MFVRFNSSHGFPVEVDSNTSIFQLKEVVAKRQGVPADQLHVIFAGKELRNDLTVQSCDLDQQSIVHVVLRPWREGQEREATRGDSPQKAMDGPEREPESLTRVDLSSSILPAHSVGLAVILNND.......AGRPAGRGTYNSFYVYCKGPCQRVQPGKLRVQCSTCRQAT MIVFVRFNSSHGFPVEVDSDTSIFQLKEVVAKRQGVPADQLRVIFAGKELRNDWTVQNCDLDQQSIVHIVQRPWRKGQEMNATGGDDPRNAAGGCEREPQSLTRVDLSSSVLPGDSVGLAVILHTDSRKDSPPAGSPAGRSIYNSFYVYCKGPCQRVQPGKLRVQCSTCRQAT CEEEEECCCCCEEEEEECCCCCHHHHHHHHHHHHCCCCCCEEEEECCCCCCCCCCCCCCCCCCCCEEEEEECCCCCCCCCCCCCCCCCCCCCCCCCCCCCCCCCCCCCCCCCCCCCCCCEEEECCCCCCCCCCCCCCCCCCCCCCCCCCCCCCCCCCCCCCCCCCCCCCCCCCCCCCC MIVFVRFNSSHGFPVEVDSDTSIFQLKEVVAKRQGVPADQLRVIFAGKELRNDWTVQNCDLDQQSIVHIVQRPWRKGQEMNATGGDDPRNAAGGCEREPQSLTRVDLSSSVLPGDSVGLAVILHTDSRKDSPPAGSPAGRSIYNSFYVYCKGPCQRVQPGKLRVQCSTCRQAT

IYNSFYVYCKGPCQRVQPGKLRVQCSTCRQATLTLTQ .............................................................................................................................................IYNSFYVYCKGPCQRVQPGKLRVQCSTCRQAT

.............................................................................................................................................CCCCEEEEECCCCEEEEEEEEEEEECCCCCCEEEECC .............................................................................................................................................IYNSFYVYCKGPCQRVQPGKLRVQCSTCRQAT .............................................................................................................................................IYNSFYVYCKGPCQRVQPGKLRVQCSTCRQAT ................................................................................................................................................SFYVYCKGPCQRVQPGKLRVRCSTCQQAT ..............................................................................................................................................YNSFYVYCKGPCQRVQPGKLRVRCSTCQQAT ..............................................................................................................................................YHSFFVYCKGPCHKVQPGKLRVQCGTCRQAT ..............................................................................................................................................YNSFYVFCKSFCQAVKPGKLRVHCRTCKQGT ..............................................................................................................................................YNSFYVYCKGPCQGVQPGKLRVRCSTCQQAT .................................................................................................................................................................................. ..........................................................................................................................................................QGVQPERLRVLFAGRELKS .................................................................................................................................................FYVYCEFPCKSIQSGKLRVRCSLCKAGA .................................................................................................................................................................................. .............................................................................................................................................ISSRFYVYCKR.CGTVTPGKLRARCSKCKESS .................................................................................................................................................FYVYCSAPCKNVTAGKLRVKCARCGSGA .................................................................................................................................................FFVYCS.QCEKVCTGKLRVRCGICKSGA ................................................................................................................................................NFYVYCTSPCANTTVGKIRVRCFKCKSGA .................................................................................................................................................FYIYCPGPCKIVTAGKLRVKCAKCNSGA .................................................................................................................................................FYVYCSAPCKDVTAGKLRVKCAKCGSGA .................................................................................................................................................FYVYCSYPCKSVEFGKLRVRCQDCGGGA .................................................................................................................................................FYVFCK.MCADVRLGKLRVCCQQCQAAT .................................................................................................................................................FFVHC.GQCDKLCNGKLRVRCSLCKGGA

**NOTE:** To save space, only 20 of 70 template profile alignments are shown above. The complete set of alignments can be found in the file hParkin__TRC2013_4bm9-a_profile.ali.

In the alignment above, 301 of 465 target residues (64.7%) are aligned to template residues. Among these aligned residues, the sequence identity is 99.7% and the sequence similarity is 99.7% ('similar' means that the BLOSUM62 score is > 0). The following 5 loops had to be modeled:

Loop N-terminal anchor 1 QARWE 2 None 3 AVFEA

4 ETIKK 5 KVTCE

Loop sequence AASKETI SRKDSPPAGSPAGRS SGTTTQA

TTK GGNGLGCG

C-terminal anchor KKTTK IYNSF YRVDE

PCPRC FAFCR

After the side-chains had been built, optimized and fine-tuned, all newly modeled parts were subjected to a combined steepest descent and simulated annealing minimization (i.e. the backbone atoms of aligned residues were kept fixed to avoid potential damage).

The resulting half-refined model has been saved as hParkin__TRC2013_4bm9-a02_refined050.yob and obtained the following quality Z-scores:

Check type Quality Z-score Comment Dihedrals 0.956 Optimal

Dihedrals

0.956

Optimal

Packing 1D

-1.393

Satisfactory

Packing 3D

-1.933

Satisfactory

Overall

-1.304

Satisfactory

Then a full unrestrained simulated annealing minimization was run for the entire model. The result has been saved as hParkin__TRC2013_4bm9-a02_refined100.yob, the corresponding Z-scores are listed below:

Since the overall quality Z-score improved to -1.284 during the minimization, this fully refined model has been accepted as the final one for this template and alignment.

**NOTE:** 126 N-terminal residues have been skipped entirely and are not part of the model, as requested by the 'TermExtension' and 'LoopLenMax' parameters. Since YASARA does not yet perform ab initio structure prediction, it would not make sense to append very long tails. If a 'tail' happens to contain an entire domain, it can usually be found in another model and joined manually, provided that the 'Templates' parameter has been set large enough to include also the other template. Nevertheless 15 terminal residues were modeled, but excluded from the Z-scores in both tables above, to minimize noise from terminal regions.

The final model for this template and alignment has been saved as hParkin__TRC2013_4bm9-a02.yob, and is shown below, together with a plot of its overall quality Z-score, shown per residue:

**6.6. Homology model 6/6, based on template 4K7D-C**

This model is a **monomer**, and based on the following profile-profile alignment: (To improve readability, only up to ten lines of the complete target multiple sequence alignment (MSA) shown above are also displayed here (upside down). The template MSA has been shrunk and focused

on structurally aligned PDB entries. The actual target-template alignment can be found in the middle at the line named 'Match'.)

D3K2X0 : MIVFVRFNSSHGFPVEVDSDTSIFQLKEVVAKRQGVPADQLRVIFAGKELRNDWTVQNCDLDQQSIVHIVQRPWRKGQEMNATGGDDPRNAAGGCEREPQSLTRVDLSSSVLPGDSVGLAVILHTDSRKDSPPAGSPAGRSIYNSFYVYCKGPCQRVQPGKLRVQCSTCRQAT B0X8E8 : ..IYVKSNTGSTLSVDLEPHMDIKDVKEIVAPQLGLAPDELKIIFAGKELSDTITISECDLGQQSIIH....PTIPSPSKNFQSNGKRRLNSIISEESPEEPYPAGSSK....................................FFVYC...CEKVCTGKLRVRCGICRSGA UPI00005: ...............................................................................................................................RSPETP........LHPSFYVYCKSHCRSVQPGKLRVCCQTCKDNA Q5J4W3 : MIVFVRYNLGPEVVVELQEEATVAELKEVVGQQQGVQPDLLRVLFAGRELKSTSTLQGCDLPEQSTVH...................PEHLSQGEEENHDSLTRLDLSASRLPTTSSTLGVI.......................FFVYCK..CKLVQPGKLRVRCRSCRQAT D3JW62 : MIVFVRFNSSYGFPVEVDSDTSIFQLKEVVAKRQGVPADQLRVIFAGKELQNHLTVQNCDLEQQSIVHIVQRPQRKSHETNASGGDKPQSTPEGSIWEPRSLTRVDLSSHILPADSVGLAVILDTDSKSDSEAARGPAAKPTYHSFFVYCKGPCHKVQPGKLRVQCGTCRQAT

Check type

Quality Z-score

Comment

Dihedrals

1.328

Optimal

Packing 1D

-1.158

Satisfactory

Packing 3D

-2.203

Poor

Overall

-1.284

Satisfactory

D3JW62 : UPI00020: D3JW61 : UPI00020: UPI0001D: O60260 : SecStr : **Target** : **Match : Template**: SecStr : Q9WVS6 : UPI00017: O60260 : D3JVU6 : F1NWU0 : Q5VVX3 : D3JW61 : E9G0L1 : D3JZW7 : C3XVY8 : UPI00016: Q7KTX7 : B3RY20 : Q7Q591 : D2Y182 : B4PE67 : E9JCM0 : F1Q9C4 : B4H5X6 : A8XSA1 :

MIVFVRFNSSYGFPVEVDSDTSIFQLKEVVAKRQGVPADQLRVIFAGKELQNHLTVQNCDLEQQSIVHIVQRPQRKSHETNASGGDKPQSTPEGSIWEPRSLTRVDLSSHILPADSVGLAVILDTDSKSDSEAARGPAAKPTYHSFFVYCKGPCHKVQPGKLRVQCGTCRQAT ..VFVRFNSSHGFPVEIDSDTSIFQLKEAVAKRQGVPADQLRVIFAGKELRNDLTVQSCDLPQQSIVHVIQTP.....................................................................YNSFYVFCKSFCQAVKPGKLRVHCRTCKQGT MKVFVRFNSNHGFPVEVDSDTSIFQLKEVVARRQGVPADQLCVIFAGKELRNDWTVQSCDLDQQSIVHIVLRPRRKG..........PRPAWGRSDRELESLTRVDLSSSVLPADSVGLAVILQDGEESGASSARRPAGRPTYNSFYVYCKGPCQGVQPGKLRVRCSTCQQAT ..MFVRFNSSHGFPVEVEADTNIFQLKEVVAKRQGVPADQLHVIFAGKELRNDLTLQNCDLDQQSIVHVVQRAQRGDQKEAMSGQNDPGHSRGVIGREPESLTRVDLSSSILPAYSVGLAVILENEDKDDSPPAG...GTPTYNSFYVFCKGGCQGVQPGKLRVRCSTCKQAT ..MFVRFNSSHGFPVEVDSNTSIFQLKEVVAKRQGVPADQLHVIFAGKELRNDLTVQSCDLDQQSIVHVVLRPWREGQEREATRGDSPQKAMDGPEREPESLTRVDLSSSILPAHSVGLAVILNND.......AGRPAGRGTYNSFYVYCKGPCQRVQPGKLRVQCSTCRQAT MIVFVRFNSSHGFPVEVDSDTSIFQLKEVVAKRQGVPADQLRVIFAGKELRNDWTVQNCDLDQQSIVHIVQRPWRKGQEMNATGGDDPRNAAGGCEREPQSLTRVDLSSSVLPGDSVGLAVILHTDSRKDSPPAGSPAGRSIYNSFYVYCKGPCQRVQPGKLRVQCSTCRQAT CEEEEECCCCCEEEEEECCCCCHHHHHHHHHHHHCCCCCCEEEEECCCCCCCCCCCCCCCCCCCCEEEEEECCCCCCCCCCCCCCCCCCCCCCCCCCCCCCCCCCCCCCCCCCCCCCCCEEEECCCCCCCCCCCCCCCCCCCCCCCCCCCCCCCCCCCCCCCCCCCCCCCCCCCCCCC MIVFVRFNSSHGFPVEVDSDTSIFQLKEVVAKRQGVPADQLRVIFAGKELRNDWTVQNCDLDQQSIVHIVQRPWRKGQEMNATGGDDPRNAAGGCEREPQSLTRVDLSSSVLPGDSVGLAVILHTDSRKDSPPAGSPAGRSIYNSFYVYCKGPCQRVQPGKLRVQCSTCRQAT

Y:SF|VYCKGPC:|VQPGKLRVQC:TCRQATLTL:Q .............................................................................................................................................TYHSFFVYCKGPCHKVQPGKLRVQCGTCRQAT

.............................................................................................................................................CCCCEEEEECCCCEEEEEEEEEEEECCCCCCEEECCC .............................................................................................................................................TYNSFFIYCKGPCHKVQPGKLRVQCGTCKQAT .............................................................................................................................................TYNSFYVYCKGPCQRVQPGKLRVRCSTCQQAT ..............................................................................................................................................YNSFYVYCKGPCQRVQPGKLRVQCSTCRQAT .............................................................................................................................................TYHSFFVYCKGPCHKVQPGKLRVQCGTCRQAT .............................................................................................................................................SYNSFYVFCKNFCQAVKPGKLRVRCNECKQGT .................................................................................................................................................................................. .............................................................................................................................................TYNSFYVYCKGPCQGVQPGKLRVRCSTCQQAT .................................................................................................................................................FYVYCEFPCKSIQSGKLRVRCSLCKAGA ..............................................................................................................................................YNSFYVYCKGPCQRVQPGKLRVQCSTCRQAT .................................................................................................................................................FFVYCKQQCKDVQPGKLRVSCATCKQGT .................................................................................................................................................................................. .................................................................................................................................................FFVHCS.QCDKLCNGKLRVRCALCKGGA .................................................................................................................................................FYVYCKR.CGTVTPGKLRARCSKCKESS .................................................................................................................................................FFVYCS.QCEKVCTGKLRVRCGICGSGA .............................................................................................................................................TYNSFYVYCKGPCQGVQPGKLRVRCSTCQQAT .................................................................................................................................................FFVHCS.QCDKLCNGKLRVRCALCKGGA .................................................................................................................................................FYVYCSAPCKDVTAGKLRVKCAKCGSGA .................................................................................................................................................................................. .................................................................................................................................................FFVHC.GQCDKLCNGKLRVRCSLCKGGA ................................................................................................................................................SFYVWCKS.CDDVRRGKLRVYCQNCESTS

**NOTE:** To save space, only 20 of 69 template profile alignments are shown above. The complete set of alignments can be found in the file hParkin__TRC2013_4k7d-c_profile.ali.

In the alignment above, 302 of 465 target residues (64.9%) are aligned to template residues. Among these aligned residues, the sequence identity is 90.4% and the sequence similarity is 94.4% ('similar' means that the BLOSUM62 score is > 0). The following 3 loops had to be modeled:

Loop N-terminal anchor Loop sequence C-terminal anchor

1 2 3

None EGECS QARWE

SRKDSPPAGSPAGRS IYNSF AVFEASGTTTQAY RVDER AASKETIKK TTKPC

After the side-chains had been built, optimized and fine-tuned, all newly modeled parts were subjected to a combined steepest descent and simulated annealing minimization (i.e. the backbone atoms of aligned residues were kept fixed to avoid potential damage).

The resulting half-refined model has been saved as hParkin__TRC2013_4k7d-c_refined050.yob and obtained the following quality Z-scores:

Check type Dihedrals Packing 1D Packing 3D Overall

Quality Z-score 1.081 -0.657 -1.540

-0.815

Comment Optimal Good Satisfactory Good

Then a full unrestrained simulated annealing minimization was run for the entire model. The result has been saved as hParkin__TRC2013_4k7d-c_refined100.yob, the corresponding Z-scores are listed below:

Comment Optimal Good Satisfactory Good

Unfortunately and the half-refined model has been accepted as the final one for this template and alignment instead.

**NOTE:** 126 N-terminal residues have been skipped entirely and are not part of the model, as requested by the 'TermExtension' and 'LoopLenMax' parameters. Since YASARA does not yet perform ab initio structure prediction, it would not make sense to append very long tails. If a 'tail' happens to contain an entire domain, it can usually be found in another model and joined manually, provided that the

Check type Dihedrals Packing 1D Packing 3D Overall

Quality Z-score 1.139 -0.452 -1.862

-0.877

the overall quality Z-score decreased to -0.877 during the minimization, making it likely that the model moved into the wrong direction, away from the real structure. It was therefore discarded,

structure prediction, it would not make sense to append very long tails. If a 'tail' happens to contain an entire domain, it can usually be found in another model and joined manually, provided that the 'Templates' parameter has been set large enough to include also the other template. Nevertheless 15 terminal residues were modeled, but excluded from the Z-scores in both tables above, to minimize noise from terminal regions.

The final model for this template and alignment has been saved as hParkin__TRC2013_4k7d-c.yob, and is shown below, together with a plot of its overall quality Z-score, shown per residue:

**7. The model ranking**

The following table lists the 6 models sorted by their overall quality Z-scores. The models have been superposed and saved together as hParkin__TRC2013.sce.

Rank Z-score Structure 1 -0.750

2 -0.815 3 -0.872 4 -1.217 5 -1.284 6 -1.364

State monomeric

monomeric monomeric monomeric monomeric monomeric

Model ID 4I1F-A

4K7D-C 4I1H-A 4BM9-A01 4BM9-A02 4K95-L

Filename

hParkin__TRC2013_4i1f-a.yob hParkin__TRC2013_4k7d-c.yob hParkin__TRC2013_4i1h-a.yob hParkin__TRC2013_4bm9-a01.yob hParkin__TRC2013_4bm9-a02.yob hParkin__TRC2013_4k95-l.yob

Original number 2

6 3 4 5 1

Residues 141-465

142-465 141-465 142-465 142-465 1-465

Comment Good

Good Good Satisfactory Satisfactory Satisfactory

**8. The hybrid model**

Finally, YASARA tried to combine the best parts of the 6 models to obtain a hybrid model, hoping to increase the models (**note** that the first transfer is simply the initial model considered most suitable for hybridization, and that the scores in the right column are not comparable to the Z-scores listed further above, since they now penalize missing and very exposed residues):

Transfer First residue Last residue Length From model Score 1 1 465 465 4K95-L **-1.793**

accuracy beyond each of the contributors. The following fragments were copied from other

1 1 465 465 4K95-L **-1.793**

350 9 401 6 188 8 311 5 232 5

7 324 The resulting hybrid model obtained the

4I1F-A 4I1F-A 4BM9-A02 4BM9-A02 4I1H-A

**-1.758 -1.755** -1.780 -1.769 -1.775

2 342 3 396 4 181 5 307 6 228

328 5

accepted accepted rejected rejected rejected

4I1H-A following quality Z-scores (this time the score includes floppy terminal tails):

-1.764

rejected

Check type Dihedrals Packing 1D Packing 3D Overall

Quality Z-score 0.881 -1.440 -2.208

Comment Optimal Satisfactory Poor Satisfactory

-1.460 The following figure shows the initial model in blue, and all hybridized parts in a different color.

This hybrid model with Z-score -1.460 was saved as the final one, hParkin__TRC2013.yob

**NOTE:** If the hybrid model scores worse than the model from which it was initially derived, this is not necessarily a bad thing, since the hybrid model often covers more residues.

**NOTE:** A Z-score describes how many standard deviations the model quality is away from the average high-resolution X-ray structure. Negative values indicate that the homology model looks worse than a high-resolution X-ray structure. The overall Z-scores for all models have been calculated as the weighted averages of the individual Z-scores using the formula Overall = 0.145*Dihedrals + 0.390*Packing1D + 0.465*Packing3D. The overall score thus captures the correctness of backbone- (Ramachandran plot) and side-chain dihedrals, as well as packing interactions. It applies to globular proteins only, and can be mislead by artificial structures like long single alpha helices (which have perfect dihedrals and are free of packing errors, since there is no packing).

Yasara Thu Dec 12 16:41:03 2013

Part 2: Consensus modeling of fragments.

**Structural Modeling Report for HUMANPARKIN-AUG2013-TRC 1. The structural modeling target HUMANPARKIN-AUG2013-TRC**

The three-dimensional structure of the following target sequence has been predicted by YASARA's homology modeling experiment:

>HUMANPARKIN-AUG2013-TRC MIVFVRFNSSHGFPVEVDSDTSIFQLKEVVAKRQGVPADQLRVIFAGKELRNDWTVQNCD LDQQSIVHIVQRPWRKGQEMNATGGDDPRNAAGGCEREPQSLTRVDLSSSVLPGDSVGLA VILHTDSRKDSPPAGSPAGRSIYNSFYVYCKGPCQRVQPGKLRVQCSTCRQATLTLTQGP SCWDDVLIPNRMSGECQSPHCPGTSAEFFFKCGAHPTSDKETSVALHLIATNSRNITCIT CTDVRSPVLVFQCNSRHVICLDCFHLYCVTRLNDRQFVHDPQLGYSLPCVAGCPNSLIKE LHHFRILGEEQYNRYQQYGAEECVLQMGGVLCPRPGCGAGLLPEPDQRKVTCEGGNGLGC GFAFCRECKEAYHEGECSAVFEASGTTTQAYRVDERAAEQARWEAASKETIKKTTKPCPR CHVPVEKNGGCMHMKCPQPQCRLEWCWNCGCEWNRVCMGDHWFDV

The target sequence contains 465 residues in 1 molecule.

**2. The modeling parameters**

The following parameters have been chosen for this target:

Modeling speed (slow = best): **Slow** Number of PSI-BLAST iterations in template search (PsiBLASTs): **8** Maximum allowed (PSI-)BLAST E-value to consider template (EValue Max): **5** Maximum number of templates to be used (Templates Total): **25** Maximum number of templates with same sequence (Templates SameSeq): **1** Maximum oligomerization state (OligoState): **4** (tetrameric) Maximum number of alignment variations per template: (Alignments): **15** Maximum number of conformations tried per loop (LoopSamples): **100** Maximum number of residues added to the termini (TermExtension): **15**

**3. The homology modeling templates**

The following 2 template structures were provided directly:

**4. The secondary structure prediction**

To aid alignment correction and loop modeling, a secondary structure prediction for the target sequence had to be obtained. This was achieved by running PSI-BLAST to create a target sequence profile and feeding it to the PSI-Pred secondary structure prediction algorithm [Jones DT, J.Mol.Biol. **292**:195-202].

The resulting prediction is listed below, the lines 'PreHel', 'PreStr' and 'PreCoi' indicate the estimated probability for the three secondary structure classes helix, strand and coil.

Sequence: MIVFVRFNSSHGFPVEVDSDTSIFQLKEVVAKRQGVPADQLRVIFAGKELRNDWTVQNCDLDQQSIVHIVQRPWRKGQEMNATGGDDPRNAAGGCEREPQSLTRVDLSSSVLPGDSVGLAVILHTDSRKDSPPAGSPAGRSIYNSFYVYCKGPCQRVQPGKLRVQCSTCRQATLTLTQGPSCWDDVLIPNRMSGECQSPHCPGTSAEFFFKCGAHPTSDKETSVALHLIATNSRN

SecStr : CEEEEECCCCCEEEEEECCCCCHHHHHHHHHHHHCCCCCCEEEEECCCCCCCCCCCCCCCCCCCCEEEEEECCCCCCCCCCCCCCCCCCCCCCCCCCCCCCCCCCCCCCCCCCCCCCCCEEEECCCCCCCCCCCCCCCCCCCCCCCCCCCCCCCCCCCCCCCCCCCCCCCCCCCCCCCCCCCCCCCCCCCCCCCCCCCCCCCCCCCHHHHHCCCCCCCCCHHHHHHHCCCCCCCC PreHel : 0000000000000000000013999999998875000110000000000000111332000000000000000111010011000000001000000001223311000000000111111111100000000000000000000100111000000001122321344443232100000012321112111010000000001345554310000013556675543100000 PreStr : 0999994100168999510000000000000000000002578984244210123333200001389998722110112211000000000000011111111221111121100122357764210000000000000123211223321001222210123443100001232221001211112322222111100000000011111110000000001231123320012 PreCoi : 9000005899831000489986000000000224999987310015744668764445688998610001277778877778899999988899888898776567888888899877431135788899999999998876788775578988666667654446544455545678998776666666677888899999998643344589999987532112344579987

**5. The target sequence profile**

To help align target and templates, a target sequence profile has been created from the following multiple sequence alignment, which is built from related UniRef90 sequences. This alignment has also been saved as humanParkin-Aug2013-TRC_profile.ali. The color codes are: negative, positive, hydrophilic and hydrophobic.

**Target** : MIVFVRFNSSHGFPVEVDSDTSIFQLKEVVAKRQGVPADQLRVIFAGKELRNDWTVQNCDLDQQSIVHIVQRPWRKGQEMNATGGDDPRNAAGGCEREPQSLTRVDLSSSVLPGDSVGLAVILHTDSRKDSPPAGSPAGRSIYNSFYVYCKGPCQRVQPGKLRVQCSTCRQATLTLTQGPSCWDDVLIPNRMSGECQSPHCPGTSAE**F**FF**KC**GA**H**PTSDKETSVA**L**HLIAT O60260 : MIVFVRFNSSHGFPVEVDSDTSIFQLKEVVAKRQGVPADQLRVIFAGKELRNDWTVQNCDLDQQSIVHIVQRPWRKGQEMNATGGDDPRNAAGGCEREPQSLTRVDLSSSVLPGDSVGLAVILHTDSRKDSPPAGSPAGRSIYNSFYVYCKGPCQRVQPGKLRVQCSTCRQATLTLTQGPSCWDDVLIPNRMSGECQSPHCPGTSAE**F**FF**KC**GA**H**PTSDKETSVA**L**HLIAT B9VH11 : MIVFVRFNSSHGFPVEVDSDTSIFQLKEVVAKRQGVPTDQLRVIFAGKELRNDWTVQNCDLDQQSIVHIVQRPRRKGQEMNATGGDNARNTAGGCEREPQSLTRVDLSSSVLPGDSVGLAVILHTDSRNDSPPAGSP..RPIYNSFYVYCKGPCQRVQPGKLRVQCSTCRQATLTLTQGPSCWDDVLIPNRMSGECQSPHCPGTTAE**F**FF**KC**GA**H**PTSDKETSVA**L**HLIAT UPI00017: ..VFVRFNSSHGFPVEVDSNTSIFQLKEAVAKRQGVPADQLRVIFAGKDLRNDLTVQSCDLDQQSIVHVVLRPQRKDQETNTPGGDKPQSA.GGSEREPESLTRVDLSSSILPTHSVGLAVILNSDCKNDVPPPGRPAGRSTYNSFYVYCKGPCQRVQPGKLRVRCSTCQQATLTLAQGPSCWEDVLIPNRMSGECQSPNCPGTRAE**F**FF**KC**GA**H**PTADKETSVA**L**NLITT UPI0000E: ........................................................QHCDLDQQSIVHIVQRPWRKGQEMNATGGDDPRNAAGGCEREPQSLTRVDLSSSVLPGDSVGLAVILHTDSRKDSPPAGSPAGRSIYNSFYVYCKGPCQRVQPGKLRVQCSTCGQATLTLTQGPSCWDDVLIPNRMSGECQSPHCPGTSAE**F**FF**KC**GA**H**PTSDKETSVA**L**HLIAT UPI0001D: ..MFVRFNSSHGFPVEVDSNTSIFQLKEVVAKRQGVPADQLHVIFAGKELRNDLTVQSCDLDQQSIVHVVLRPWREGQEREATRGDSPQKAMDGPEREPESLTRVDLSSSILPAHSVGLAVILNND.......AGRPAGRGTYNSFYVYCKGPCQRVQPGKLRVQCSTCRQATLTLAQGPSCWDDVLIPNRMTGECQSANCPGTAAE**F**FF**KC**GA**H**PTSDKETSVA**L**NLITT Q1WDP3 : MIVFVRFNSSHGFPVEVDSDTSIFQLKEVVAKRQGVPADQLRVIFAGKELRNDLTVQRCDLDQQSIVHVVLRPQRNGQERGVAAGHRP........REPASLTRVDLSGSVLPGDAVGLAVILQDDSADGAAPAGRPADRPTNKSFYVYCKGPCQRVQPGKLRVRCSTCQQATLTLTQGPSCWDDVLIPNRMSGECQSPNCPGTTAE**F**FF**KC**GA**H**PTSDKETSVA**L**NLITT Q9WVS6 : MIVFVRFNSSYGFPVEVDSDTSILQLKEVVAKRQGVPADQLRVIFAGKELPNHLTVQNCDLEQQSIVHIVQRPRRRSHETNASGGDEPQSTSEGSIWESRSLTRVDLSSHTLPVDSVGLAVILDTDSKRDSEAARGP.....YNSFFIYCKGPCHKVQPGKLRVQCGTCKQATLTLAQGPSCWDDVLIPNRMSGECQSPDCPGTRAE**F**FF**KC**GA**H**PTSDKDTSVA**L**NLIT F1MA69 : .LVFVRFNSSYGFPVEVDSDTSIFQLKEVVAKRQGVPADQLRVIFAGKELQNHLT..NCDLEQQSIVHIVQRPQRKSHETNASGGDKPQSTPEGSIWEPRSLTRVDLSSHILPADSVGLAVILDTDSKSDSEAARGPEAKPTYHSFFVYCKGPCHKVQPGKLRVQCGTCRQATLTLAQGPSCWDDVLIPNRMSGECQSPDCPGTRAE**F**FF**KC**GA**H**PTSDKDTSVA**L**NLIT UPI00020: ..MFVRFNSSHGFPVEVEADTNIFQLKEVVAKRQGVPADQLHVIFAGKELRNDLTLQNCDLDQQSIVHVVQRAQRGDQKEAMSGQNDPGHSRGVIGREPESLTRVDLSSSILPAYSVGLAVILENEDKDDSPPAG...GTPTYNSFYVFCKGGCQGVQPGKLRVRCSTCKQATLILDQGPSCWDDVLISNKISGVCHFPDCNGTGAE**F**YF**KC**GA**H**PTSANETSVA**L**NLITT UPI0000E: MKVFVRFNSNHGFPVEVDSDTSIFQLKEVVARRQGVPADQLCVIFAGKELRNDWTVQSCDLDQQSIVHIVLRPRRKGPE.....GHSPRPAWGRSDREPESLTRVDLSSSMLPADSVGLAVILQDGEESGASSARRPAGRPTYNSFYVYCKGPCQGVQPGKLRVRCSTCQQATLTLAQGPSCWEDVLIPNRMSGECQSPNCPGTRAE**F**FF**KC**GA**H**PTSDKETSVA**L**NLITT UPI00019: ..VFVRFNSSHGFPVEVGSDSSILQLKEAVAQRQGVPADQLRVIFAGRELSNDLTLQNCDLAQQSIVHIVESPQKNSQDKEKTEYSCVGGVPKALKREPESLTRIDLSTSILPSVSAGLAVI...DPGKNMPFADSPASRASYNSFYVFCKNFCQAVKPGKLRVRCSVCKQGTLTLARGPSCWDDVLIPNRIGGVCQSQGCNGNVAE**F**YF**KC**GA**H**PTTDSETSVA**L**NLVTT F1NWU0 : ..VFVRFNSSHGFPVELGLDASILQLKEAVAQRQGVPADQLRVIFAGRELSNDLTLQNCDLVQQSIVHIVQ.......DKDETEDNHAGGILKTLERVPESLTRIDLSSSILPSLSAGLAVILDTKEPNISPPSEKSAGAASYNSFYVFCKNFCQAVKPGKLRVRCNECKQGTLTLARGPSCWDDVLIPNRITGVCQSPDCSGNVAE**F**YF**KC**GA**H**PTTDSETSVA**L**NLVTT D3JW61 : MKVFVRFNSNHGFPVEVDSDTSIFQLKEVVARRQGVPADQLCVIFAGKELRNDWTVQSCDLDQQSIVHIVLRPRRKG..........PRPAWGRSDRELESLTRVDLSSSVLPADSVGLAVILQDGEESGASSARRPAGRPTYNSFYVYCKGPCQGVQPGKLRVRCSTCQQATLTLAQGPSCWEDVLIPNRMSGECQSPNCPGTRAE**F**FF**KC**GA**H**PTSDKETSVA**L**NLITT D3JZW7 : MIVFVRFNSSHGFPVEVDSDTSIFQLKEVVAKRQGVPADQLRVIFAGKELRNDWTVQNCDLDQQSIVHIVQRPWRKGQEMNATGGDDPRNAAGGCEREPQSLTRVDLSSSVLPGDSVGLAVILHTDSRKDSPPAGSPAGRSIYNSFYVYCKGPCQRVQPGKLRVQCSTCRQATLTLTQGPSCWDDVLIPNRMSGECQSPHCPGTSAE**F**FF**KC**GA**H**PTSDKETSVA**L**HLIAT Q561U2 : MIVFVRFNSSHGFPVELEQGASVSELKEAVGRLQGVQSDQLRVIFAGRELCNESTLQGCDLPEQSTVHVVLPP...............RRLGSG.......LTRLDLSSSRQTTASEGLAVILETEASRREDTAG.......HSSFYVFCKTVCKAIQPGKLRVRCKDCKQGTLTLSRGPSCWDDVLLPNRIHGVCQSQGCNGRLAE**F**YL**KC**AS**H**PTCDNDTSVA**L**DLIMP D3JVU6 : MIVFVRFNSSYGFPVEVDSDTSIFQLKEVVAKRQGVPADQLRVIFAGKELQNHLTVQNCDLEQQSIVHIVQRPQRKSHETNASGGDKPQSTPEGSIWEPRSLTRVDLSSHILPADSVGLAVILDTDSKSDSEAARGPA....YHSFFVYCKGPCHKVQPGKLRVQCGTCRQATLTL..GPSCWDDVLIPNRMSGECQSPDCPGTRAE**F**FF**KC**GA**H**PTSDKDTSVA**L**NLIT UPI00020: ..VFVRFNSSHGFPVEIDSDTSIFQLKEAVAKRQGVPADQLRVIFAGKELRNDLTVQSCDLPQQSIVHVIQTP.....................................................................YNSFYVFCKSFCQAVKPGKLRVHCRTCKQGTLTLSRGPSCWEDVLIPNRITGVCQSRNCNGEVAE**F**YF**KC**GA**H**PTSDSETSVA**L**NLITT Q5VVX4 : MIVFVRFNSSHGFPVEVDSDTSIFQLKEVVAKRQGVPADQLRVIFAGKELRNDWTVQNCDLDQQSIVHIVQRPWRKGQEMNATGGDDPRNAAGGCEREPQSLTRVDLSSSVLPGDSVGLAVILHTDSRKDSPPAGSPAGRSIYNSFYVYCKGPCQRVQPGKLRVQCSTCRQATLTLTQ............................E**F**FF**KC**GA**H**PTSDKETSVA**L**HLIAT Q5VVX3 : ...............................................................................................................................................................................................................**F**FF**KC**GA**H**PTSDKETSVA**L**HLIAT Q19K47 : MIVFVRFNSSHGFPVEVDSDTSIFQLKEVVAKRQGVPADQLRVIFAGKELRNDLTVQRCDLDQQSIVHVVLRPQRNGQERGVAAGHRP........REPASLTRVDLSGSVLPGDAVGLAVILQDDSADGAAPAGRP.....NKSFYVYCKGPCQRVQPGKLRVRCSTCQQATLTLTQGPSCWDDVLIPNRMSGECQSPNCPGTTAE**F**FF**KC**GA**H**PTSDKETSVA**L**NLITT D3JW62 : MIVFVRFNSSYGFPVEVDSDTSIFQLKEVVAKRQGVPADQLRVIFAGKELQNHLTVQNCDLEQQSIVHIVQRPQRKSHETNASGGDKPQSTPEGSIWEPRSLTRVDLSSHILPADSVGLAVILDTDSKSDSEAARGPAAKPTYHSFFVYCKGPCHKVQPGKLRVQCGTCRQATLTLAQ............................E**F**FF**KC**GA**H**PTSDKDTSVA**L**NLIT

Template

Object

Name

Resolution

Amino acids

1

1

T001

2.00 A

306

2

2

T002

Unknown or NMR

141

I

E 0 5 4

D3JW62 : MIVFVRFNSSYGFPVEVDSDTSIFQLKEVVAKRQGVPADQLRVIFAGKELQNHLTVQNCDLEQQSIVHIVQRPQRKSHETNASGGDKPQSTPEGSIWEPRSLTRVDLSSHILPADSVGLAVILDTDSKSDSEAARGPAAKPTYHSFFVYCKGPCHKVQPGKLRVQCGTCRQATLTLAQ............................E**F**FF**KC**GA**H**PTSDKDTSVA**L**NLIT UPI00016: ..VFVRYNLGPEVVVELQEEATVAELKEVVGQQQGVQPDLLRVLFAGRELKSTSTLQGCDLPEQSTVH...................PEHLSQGEEENHDSLTRLDLSASRLPTTSSTLGVILERNDSEGSAGAKDHSVR....TFFVYCK..CKLVQPGKLRVRCRSCRQATLTLSRGPSCWDDVLLQSRVHGVCHSDGCHGTEAE**F**YM**KC**AS**H**PTSDNDHSVA**L**DLIMT D2Y182 : MKVFVRFNSNHGFPVEVDSDTSIFQLKEVVARRQGVPADQLCVIFAGKELRNDWTVQSCDLDQQSIVHIVLRPRRKGPE.......SPRPAWGRSDRELESLTRVDLSSSVLPADSVGLAVILQDGEESGASSARRPAGRPTYNSFYVYCKGPCQGVQPGKLRVRCSTCQQATLTLAQGPSCWEDVLIPNRMSGECQSPNCPGTRAE**F**FF**KC**GA**H**PTSDKETSVA**L**NLITT UPI00017: ..VIVRYNLGPEVVVEVQEEATVAELKEVVARQQGVQPERLRVLFAGRELKSTSTLQDCDLPEQSTVH...........................EEDHDSLTRLDLSSSRLTTTTSGLAVIL.................SV...FFVYCK..CKSIQPGKLRVRCRSCRQTTLTLSRGPSCWDDVLLRNRLHGVCHSDGCHGTEAE**F**YM**KC**AR**H**PTSDSDHSVA**L**DLIMT Q5J4W3 : MIVFVRYNLGPEVVVELQEEATVAELKEVVGQQQGVQPDLLRVLFAGRELKSTSTLQGCDLPEQSTVH...................PEHLSQGEEENHDSLTRLDLSASRLPTTSSTLGVI.......................FFVYCK..CKLVQPGKLRVRCRSCRQATLTLSRGPSCWDDVLLQSRVHGVCHSDGCHGTEAE**F**YM**KC**AS**H**PTSDNDHSVA**L**DLIMT C1BKS6 : MIVYVRFNSSHGFPLELEEGTSIAKLKETVGRLQGVQGEHLRVIFAGRELRSDSTLQGCDLPEQSTVHVVLPP...........................SLTRLDLSASRLPTTSTGLAVILETDEHRARELAEEHAGPRPHSSFYVYCKSVCGAIQPAKLRVRCSVCKQGTLTLSRGPSCWDDVLLPERIHGVCQSEGCHGRIAE**F**YL**KC**AT**H**PTSDDDTSVA**L**DLIMP D3JZW4 : MIVFVRFNSSYGFPVEVDSDTSIFQLKEVVAKRQGVPADQLRVIFAGKELQNHLTVQNCDLEQQSIVHIVQRPQRKSHETNASGGDKPQSTPEGSIWEPRSLTRVDLSSHILPADSVGLAVILDTDSKSDSEAARGPAAKPTYHSFFVYCKGPCHKVQPGKLRVQCGTCRQATLTLAQGPSCWDDVLIPNRMSGECQSPDCPGTRAE**F**FF**KC**GA**H**PTSDKDTSVA**L**NLIT E9G0L1 : ..VHIKAAGGKTFLIQMSRDWDVARIKKFIAPKVGLKVEDISIILAGKSLADDLLLEECDLGHNSILNAVKLKVIKKADTTSS..............................................................FYVYCEFPCKSIQSGKLRVRCSLCKAGAFTVDRDPCCWDDVLRPEQVSGTCQMEDCDHSWAE**F**YF**KC**AH**H**P....DSTLP**L**NLVKS UPI00005: ...............................................................................................................................RSPETP........LHPSFYVYCKSHCRSVQPGKLRVCCQTCKDNAFIVKEDPVCWDDVILSNRISGSCFVPGCQGQKAE**F**FF**KC**SS**H**ASSVNDQFTV**L**PLVK D2Y181 : MKVFVRFNSNHGFPVEVDSDTSIFQLKEVVARRQGVPADQLCVIFAGKELRNDWTVQSCDLDQQSIVHIVLRPRRKGPE........PRPAWGRSDRELESLTRVDLSSSVLPADSVGLAVILQDGEESGASSARRPAGRPTYNSFYVYCKGPCQGVQPGKLRVRCSTCQQATLTLAQGPSCWEDVLIPNRMSGECQSPNCPGTRAE**F**FF**KC**GA**H**PTSDKETSVA**L**NLITT C3Z502 : ..VMVRFNSNHSFLVTVHTSWTIARFKQEVGRTQGVPSGQIHILFAGRDLSDSLRIEDCQLGQQTVIHAI................DARSSA......PRSLS.........................................SYFVFCKRPCKAVRPGKLRVRCGTCRQTTLTLSRDPNCWEDVLVPGKIQGRCLSRGCPGTVAE**F**YF**KC**AD**H**HTSEDDTSVA**L**PLVKS Q7KTX7 : ..IYVKTNTGKTLTVNLEPQWDIKNVKELVAPQLGLQPDDLKIIFAGKELSDATTIEQCDLGQQSVLH.............................................................................FFVHC...CDKLCNGKLRVRCALCKGGAFTVHRDPECWDDVLKSRRIPGHCE.........E**F**FF**KC**AE**H**.......AAP**L**NLIKN B0X8E8 : ..IYVKSNTGSTLSVDLEPHMDIKDVKEIVAPQLGLAPDELKIIFAGKELSDTITISECDLGQQSIIH....PTIPSPSKNFQSNGKRRLNSIISEESPEEPYPAGSSK....................................FFVYC...CEKVCTGKLRVRCGICRSGAFTVHRDPESWDDVLKRKRITGHCENYE......E**F**YF**KC**AE**H**..........**L**NLIK Q17DC3 : ..IYVKTNTGNTLSVDLEPHMDIKDVKEIVAPQLGLAPGELKIIFAGKELSDTITISECDLGQQSIIHAV................................................................GSPARERRKAHFFVYC...CEKVCTGKLRVRCGICKSGAFTVHRDPACWDDVLKKKRITGHCENYEIP.....**F**YF**KC**SE**H**...EKDFAAP**L**NLIK Q4S2K5 : ............................................................................................................................................................VQPERLRVLFAGRELKSTSTLQGPSCWDDVLLRNRLHGVCHSDGCHGTEAE**F**YM**KC**AR**H**PTSDSDHSVA**L**DLIMT E3WZE5 : ..VYVKTNTGSTLAVSLEPHMEIKEVKEMVAPQLGLEPAELKIIFAGRELSDTTTIRECDLGQQSIIHAV....RRGQQKQSLGG.................................................GAPAER.....FFVYC...CEKVCTGKLRVRCGICGSGAFTVHRDPACWDDVLKRKRITGHCEQN.......E**F**FF**KC**SE**H**S...KDFAAP**L**NLIK D3K2X0 : MIVFVRFNSSHGFPVEVDSDTSIFQLKEVVAKRQGVPADQLRVIFAGKELRNDWTVQNCDLDQQSIVHIVQRPWRKGQEMNATGGDDPRNAAGGCEREPQSLTRVDLSSSVLPGDSVGLAVILHTDSRKDSPPAGSPAGRSIYNSFYVYCKGPCQRVQPGKLRVQCSTCRQATLTLTQ............................E**F**FF**KC**GA**H**PTSDKETSVA**L**HLIAT C3XVY8 : MQVFVRFNSHHSFPVDVDSSWSVLQLKEVLAARQQVPPAEIRIIFAGRELRDSFIIGECDLASHSIVHVV..........................QEPQSLTRVDL......................................FFVYCKQQCKDVQPGKLRVSCATCKQGTLT...............................E**F**YF**KC**AA**H**PTQEEDTAAA**L**YMIKS UPI00016: ..VFVRYNLGPEVVVELQEEATVAELKEVVGQQQGVQPDLLRVLFAGRELKSTSTLQGCDLPEQSTVH.....................HLSQGEEENHDSLTRLDLSASRLPTTSSTLGVIL......DSEGVGATAGGGGADTFFVYCK..CKLVQPGKLRVRCRSCRQATLTL..............................E**F**YM**KC**AS**H**PTSDNDHSVA**L**DLIMT B3RY20 : ..........................................................................................................................................GHNISSRFYVYCK..CGTVTPGKLRARCSKCKESSVTLVRGPESWDDILIYGRIKGTCQ...CSNTIVE**F**YF**KC**GS**H**LADENEFSAP**L**YQIR UPI00016: ...........................................................................................................................................................................................................TLQE**F**YM**KC**AS**H**PTSDNDHSVA**L**DLIMT

**6. The initial homology models**

For each of the templates listed above, models were built. Either a single model if the alignment was certain, or a number of alternative models if the alignment was ambiguous.

**6.1. Homology model 1/2, based on template T001-_**

This model is a **monomer**, and based on the following profile-profile alignment:

(To improve readability, only up to ten lines of the complete target multiple sequence alignment (MSA) shown above are also displayed here (upside down). The template MSA has been shrunk and focused on structurally aligned PDB entries. The actual target-template alignment can be found in the middle at the line named 'Match'.)

D3K2X0 : B0X8E8 : UPI00005: Q5J4W3 : D3JW62 : UPI00020: D3JW61 : UPI00020: UPI0001D: O60260 : SecStr : **Target** : **Match : Template**: SecStr : UPI0000E: B9VH11 : Q1WDP3 : F1MA69 : D3JVU6 : UPI00020: Q5VVX4 : Q5VVX3 : Q561U2 : D3JW61 : Q5J4W3 : UPI00017: UPI00005: Q4S2K5 : Q9JK66-6: C3XVY8 : B3RY20 : E0VIU9 : Q8K5C2 : D6WJ25 :

MIVFVRFNSSHGFPVEVDSDTSIFQLKEVVAKRQGVPADQLRVIFAGKELRNDWTVQNCDLDQQSIVHIVQRPWRKGQEMNATGGDDPRNAAGGCEREPQSLTRVDLSSSVLPGDSVGLAVILHTDSRKDSPPAGSPAGRSIYNSFYVYCKGPCQRVQPGKLRVQCSTCRQATLTLTQ............................E**F**FF**KC**GA**H**PTSDKETSVA**L**HLIAT ..IYVKSNTGSTLSVDLEPHMDIKDVKEIVAPQLGLAPDELKIIFAGKELSDTITISECDLGQQSIIH....PTIPSPSKNFQSNGKRRLNSIISEESPEEPYPAGSSK....................................FFVYC...CEKVCTGKLRVRCGICRSGAFTVHRDPESWDDVLKRKRITGHCENYE......E**F**YF**KC**AE**H**..........**L**NLIK ...............................................................................................................................RSPETP........LHPSFYVYCKSHCRSVQPGKLRVCCQTCKDNAFIVKEDPVCWDDVILSNRISGSCFVPGCQGQKAE**F**FF**KC**SS**H**ASSVNDQFTV**L**PLVK MIVFVRYNLGPEVVVELQEEATVAELKEVVGQQQGVQPDLLRVLFAGRELKSTSTLQGCDLPEQSTVH...................PEHLSQGEEENHDSLTRLDLSASRLPTTSSTLGVI.......................FFVYCK..CKLVQPGKLRVRCRSCRQATLTLSRGPSCWDDVLLQSRVHGVCHSDGCHGTEAE**F**YM**KC**AS**H**PTSDNDHSVA**L**DLIMT MIVFVRFNSSYGFPVEVDSDTSIFQLKEVVAKRQGVPADQLRVIFAGKELQNHLTVQNCDLEQQSIVHIVQRPQRKSHETNASGGDKPQSTPEGSIWEPRSLTRVDLSSHILPADSVGLAVILDTDSKSDSEAARGPAAKPTYHSFFVYCKGPCHKVQPGKLRVQCGTCRQATLTLAQ............................E**F**FF**KC**GA**H**PTSDKDTSVA**L**NLIT ..VFVRFNSSHGFPVEIDSDTSIFQLKEAVAKRQGVPADQLRVIFAGKELRNDLTVQSCDLPQQSIVHVIQTP.....................................................................YNSFYVFCKSFCQAVKPGKLRVHCRTCKQGTLTLSRGPSCWEDVLIPNRITGVCQSRNCNGEVAE**F**YF**KC**GA**H**PTSDSETSVA**L**NLITT MKVFVRFNSNHGFPVEVDSDTSIFQLKEVVARRQGVPADQLCVIFAGKELRNDWTVQSCDLDQQSIVHIVLRPRRKG..........PRPAWGRSDRELESLTRVDLSSSVLPADSVGLAVILQDGEESGASSARRPAGRPTYNSFYVYCKGPCQGVQPGKLRVRCSTCQQATLTLAQGPSCWEDVLIPNRMSGECQSPNCPGTRAE**F**FF**KC**GA**H**PTSDKETSVA**L**NLITT ..MFVRFNSSHGFPVEVEADTNIFQLKEVVAKRQGVPADQLHVIFAGKELRNDLTLQNCDLDQQSIVHVVQRAQRGDQKEAMSGQNDPGHSRGVIGREPESLTRVDLSSSILPAYSVGLAVILENEDKDDSPPAG...GTPTYNSFYVFCKGGCQGVQPGKLRVRCSTCKQATLILDQGPSCWDDVLISNKISGVCHFPDCNGTGAE**F**YF**KC**GA**H**PTSANETSVA**L**NLITT ..MFVRFNSSHGFPVEVDSNTSIFQLKEVVAKRQGVPADQLHVIFAGKELRNDLTVQSCDLDQQSIVHVVLRPWREGQEREATRGDSPQKAMDGPEREPESLTRVDLSSSILPAHSVGLAVILNND.......AGRPAGRGTYNSFYVYCKGPCQRVQPGKLRVQCSTCRQATLTLAQGPSCWDDVLIPNRMTGECQSANCPGTAAE**F**FF**KC**GA**H**PTSDKETSVA**L**NLITT MIVFVRFNSSHGFPVEVDSDTSIFQLKEVVAKRQGVPADQLRVIFAGKELRNDWTVQNCDLDQQSIVHIVQRPWRKGQEMNATGGDDPRNAAGGCEREPQSLTRVDLSSSVLPGDSVGLAVILHTDSRKDSPPAGSPAGRSIYNSFYVYCKGPCQRVQPGKLRVQCSTCRQATLTLTQGPSCWDDVLIPNRMSGECQSPHCPGTSAE**F**FF**KC**GA**H**PTSDKETSVA**L**HLIAT CEEEEECCCCCEEEEEECCCCCHHHHHHHHHHHHCCCCCCEEEEECCCCCCCCCCCCCCCCCCCCEEEEEECCCCCCCCCCCCCCCCCCCCCCCCCCCCCCCCCCCCCCCCCCCCCCCCEEEECCCCCCCCCCCCCCCCCCCCCCCCCCCCCCCCCCCCCCCCCCCCCCCCCCCCCCCCCCCCCCCCCCCCCCCCCCCCCCCCCCCHHHHHCCCCCCCCCHHHHHHHCCCCCCCC MIVFVRFNSSHGFPVEVDSDTSIFQLKEVVAKRQGVPADQLRVIFAGKELRNDWTVQNCDLDQQSIVHIVQRPWRKGQEMNATGGDDPRNAAGGCEREPQSLTRVDLSSSVLPGDSVGLAVILHTDSRKDSPPAGSPAGRSIYNSFYVYCKGPCQRVQPGKLRVQCSTCRQATLTLTQGPSCWDDVLIPNRMSGECQSPHCPGTSAE**F**FF**KC**GA**H**PTSDKETSVA**L**HLIAT

SIYNSFYVYCKGPCQRVQPGKLRVQCSTCRQATLTLTQGPSCWDDVLIPNRMSGECQSPHCPGTSAEFFFKCGAHPTSDKETSVALHLIATNSRN ............................................................................................................................................SIYNSFYVYCKGPCQRVQPGKLRVQCSTCRQATLTLTQGPSCWDDVLIPNRMSGECQSPHCPGTSAEFFFKCGAHPTSDKETSVALHLIAT

............................................................................................................................................CCCCCEEEEECCCCEEEEEEEEEEEECCCCCCEEEECCCCCCHHHHHHCCCCEEEECTTTTCCCEEEEEEEECCCCCTTTTCCCCTTTTECTTTT ............................................................................................................................................SIYNSFYVYCKGPCQRVQPGKLRVQCSTCGQATLTLTQGPSCWDDVLIPNRMSGECQSPHCPGTSAEFFFKCGAHPTSDKETSVALHLIAT .............................................................................................................................................IYNSFYVYCKGPCQRVQPGKLRVQCSTCRQATLTLTQGPSCWDDVLIPNRMSGECQSPHCPGTTAEFFFKCGAHPTSDKETSVALHLIAT ................................................................................................................................................SFYVYCKGPCQRVQPGKLRVRCSTCQQATLTLTQGPSCWDDVLIPNRMSGECQSPNCPGTTAEFFFKCGAHPTSDKETSVALNLITT ..............................................................................................................................................YHSFFVYCKGPCHKVQPGKLRVQCGTCRQATLTLAQGPSCWDDVLIPNRMSGECQSPDCPGTRAEFFFKCGAHPTSDKDTSVALNLIT ..............................................................................................................................................YHSFFVYCKGPCHKVQPGKLRVQCGTCRQATLTL..GPSCWDDVLIPNRMSGECQSPDCPGTRAEFFFKCGAHPTSDKDTSVALNLIT ............................................................................................................................................STYNSFYVFCKSFCQAVKPGKLRVHCRTCKQGTLTLSRGPSCWEDVLIPNRITGVCQSRNCNGEVAEFYFKCGAHPTSDSETSVALNLITT ............................................................................................................................................SIYNSFYVYCKGPCQRVQPGKLRVQCSTCRQATLTLTQ............................EFFFKCGAHPTSDKETSVALHLIAT ............................................................................................................................................................................................................VQEFFFKCGAHPTSDKETSVALHLIAT ..............................................................................................................................................HSSFYVFCKTVCKAIQPGKLRVRCKDCKQGTLTLSRGPSCWDDVLLPNRIHGVCQSQGCNGRLAEFYLKCASHPTCDNDTSVALDLIMP ..............................................................................................................................................YNSFYVYCKGPCQGVQPGKLRVRCSTCQQATLTLAQGPSCWEDVLIPNRMSGECQSPNCPGTRAEFFFKCGAHPTSDKETSVALNLITT ...............................................................................................................................................STFFVYCK..CKLVQPGKLRVRCRSCRQATLTLSRGPSCWDDVLLQSRVHGVCHSDGCHGTEAEFYMKCASHPTSDNDHSVALDLIMT ................................................................................................................................................TFFVYCK..CKSIQPGKLRVRCRSCRQTTLTLSRGPSCWDDVLLRNRLHGVCHSDGCHGTEAEFYMKCARHPTSDSDHSVALDLIMT ............................................................................................................................................SLHPSFYVYCKSHCRSVQPGKLRVCCQTCKDNAFIVKEDPVCWDDVILSNRISGSCFVPGCQGQKAEFFFKCSSHASSVNDQFTVLPLVK ..........................................................................................................................................................QGVQPERLRVLFAGRELKSTSTLQGPSCWDDVLLRNRLHGVCHSDGCHGTEAEFYMKCARHPTSDSDHSVALDLIMT ...............................................................................................................................................................................................MSGECQSPDCPGTRAEFFFKCGAHPTSDKDTSVALNLIT .................................................................................................................................................FFVYCKQQCKDVQPGKLRVSCATCKQGTLTL..............................EFYFKCAAHPTQEEDTAAALYMIKS ............................................................................................................................................NISSRFYVYCK..CGTVTPGKLRARCSKCKESSVTLVRGPESWDDILIYGRIKGTCQ...CSNTIVEFYFKCGSHLADENEFSAPLYQIR .................................................................................................................................................FFIYCANPCKKINTGKLRVCCSECKHGAFTVDTDPQSWADVLDKNKITGVCNNVGCEGLYAKFYFKCASHP.....TAVPLNLIKR ........................................................................................................................................................................................................................................... .................................................................................................................................................FFVYC...CKALKNGKLRVRCHFCKSGAFTVHSDPQNWNDVLQSKQITGLC........FAEFYFKCSEH..........LYLIR

**NOTE:** To save space, only 20 of 45 template profile alignments are shown above. The complete set of alignments can be found in the file humanParkin-Aug2013-TRC_t001_profile.ali.

In the alignment above, 306 of 465 target residues (65.8%) are aligned to template residues. Among these aligned residues, the sequence identity is 100.0% and the sequence similarity is 100.0% ('similar' means that the BLOSUM62 score is > 0). The following 3 loops had to be modeled:

After the side-chains had been built, optimized and fine-tuned, all newly modeled parts were subjected to a combined steepest descent and simulated annealing minimization (i.e. the backbone atoms of aligned residues were kept fixed to avoid potential damage).

The resulting half-refined model has been saved as humanParkin-Aug2013-TRC_t001-__refined050.yob and obtained the following quality Z-scores:

Loop

N-terminal anchor

Loop sequence

C-terminal anchor

1

None

DSRKDSPPAGSPAGR

SIYNS

2

HEGEC

SAVFEASGTTTQAYR

VDERA

3

KVTCE

GGNGL

GCGFA

Check type

Quality Z-score

Comment

Dihedrals

1.330

Optimal

Packing 1D

-1.080

Satisfactory

Packing 3D

-1.494

Satisfactory

Overall

-0.923

Good

E I C

.

Then a full unrestrained simulated annealing minimization was run for the entire model. The result has been saved as humanParkin-Aug2013-TRC_t001-__refined100.yob, the corresponding Z-scores are listed below:

Unfortunately the overall quality Z-score decreased to -0.971 during the minimization, making it likely that the model moved into the wrong direction, away from the real structure. It was therefore discarded, and the half-refined model has been accepted as the final one for this template and alignment instead.

**NOTE:** 125 N-terminal residues have been skipped entirely and are not part of the model, as requested by the 'TermExtension' and 'LoopLenMax' parameters. Since YASARA does not yet perform ab initio structure prediction, it would not make sense to append very long tails. If a 'tail' happens to contain an entire domain, it can usually be found in another model and joined manually, provided that the 'Templates' parameter has been set large enough to include also the other template. Nevertheless 15 terminal residues were modeled, but excluded from the Z-scores in both tables above, to minimize noise from terminal regions.

The final model for this template and alignment has been saved as humanParkin-Aug2013-TRC_t001-_.yob, and is shown below, together with a plot of its overall quality Z-score, shown per residue:

**6.2. Homology model 2/2, based on template T002-_**

This model is a **monomer**, and based on the following profile-profile alignment: (To improve readability, only up to ten lines of the complete target multiple sequence alignment (MSA) shown above are also displayed here (upside down). The template MSA has been shrunk and focused

on structurally aligned PDB entries. The actual target-template alignment can be found in the middle at the line named 'Match'.)

D3K2X0 : MIVFVRFNSSHGFPVEVDSDTSIFQLKEVVAKRQGVPADQLRVIFAGKELRNDWTVQNCDLDQQSIVHIVQRPWRKGQEMNATGGDDPRNAAGGCEREPQSLTRVDLSSSVLPGDSVGLAVILHTDSRKDSPPAGSPAGRSIYNSFYVYCKGPCQRVQPGKLRVQCSTCRQATLTLTQ............................E**F**FF**KC**GA**H**PTSDKETSVA**L**HLIAT B0X8E8 : ..IYVKSNTGSTLSVDLEPHMDIKDVKEIVAPQLGLAPDELKIIFAGKELSDTITISECDLGQQSIIH....PTIPSPSKNFQSNGKRRLNSIISEESPEEPYPAGSSK....................................FFVYC...CEKVCTGKLRVRCGICRSGAFTVHRDPESWDDVLKRKRITGHCENYE......E**F**YF**KC**AE**H**..........**L**NLIK UPI00005: ...............................................................................................................................RSPETP........LHPSFYVYCKSHCRSVQPGKLRVCCQTCKDNAFIVKEDPVCWDDVILSNRISGSCFVPGCQGQKAE**F**FF**KC**SS**H**ASSVNDQFTV**L**PLVK Q5J4W3 : MIVFVRYNLGPEVVVELQEEATVAELKEVVGQQQGVQPDLLRVLFAGRELKSTSTLQGCDLPEQSTVH...................PEHLSQGEEENHDSLTRLDLSASRLPTTSSTLGVI.......................FFVYCK..CKLVQPGKLRVRCRSCRQATLTLSRGPSCWDDVLLQSRVHGVCHSDGCHGTEAE**F**YM**KC**AS**H**PTSDNDHSVA**L**DLIMT D3JW62 : MIVFVRFNSSYGFPVEVDSDTSIFQLKEVVAKRQGVPADQLRVIFAGKELQNHLTVQNCDLEQQSIVHIVQRPQRKSHETNASGGDKPQSTPEGSIWEPRSLTRVDLSSHILPADSVGLAVILDTDSKSDSEAARGPAAKPTYHSFFVYCKGPCHKVQPGKLRVQCGTCRQATLTLAQ............................E**F**FF**KC**GA**H**PTSDKDTSVA**L**NLIT UPI00020: ..VFVRFNSSHGFPVEIDSDTSIFQLKEAVAKRQGVPADQLRVIFAGKELRNDLTVQSCDLPQQSIVHVIQTP.....................................................................YNSFYVFCKSFCQAVKPGKLRVHCRTCKQGTLTLSRGPSCWEDVLIPNRITGVCQSRNCNGEVAE**F**YF**KC**GA**H**PTSDSETSVA**L**NLITT D3JW61 : MKVFVRFNSNHGFPVEVDSDTSIFQLKEVVARRQGVPADQLCVIFAGKELRNDWTVQSCDLDQQSIVHIVLRPRRKG..........PRPAWGRSDRELESLTRVDLSSSVLPADSVGLAVILQDGEESGASSARRPAGRPTYNSFYVYCKGPCQGVQPGKLRVRCSTCQQATLTLAQGPSCWEDVLIPNRMSGECQSPNCPGTRAE**F**FF**KC**GA**H**PTSDKETSVA**L**NLITT UPI00020: ..MFVRFNSSHGFPVEVEADTNIFQLKEVVAKRQGVPADQLHVIFAGKELRNDLTLQNCDLDQQSIVHVVQRAQRGDQKEAMSGQNDPGHSRGVIGREPESLTRVDLSSSILPAYSVGLAVILENEDKDDSPPAG...GTPTYNSFYVFCKGGCQGVQPGKLRVRCSTCKQATLILDQGPSCWDDVLISNKISGVCHFPDCNGTGAE**F**YF**KC**GA**H**PTSANETSVA**L**NLITT UPI0001D: ..MFVRFNSSHGFPVEVDSNTSIFQLKEVVAKRQGVPADQLHVIFAGKELRNDLTVQSCDLDQQSIVHVVLRPWREGQEREATRGDSPQKAMDGPEREPESLTRVDLSSSILPAHSVGLAVILNND.......AGRPAGRGTYNSFYVYCKGPCQRVQPGKLRVQCSTCRQATLTLAQGPSCWDDVLIPNRMTGECQSANCPGTAAE**F**FF**KC**GA**H**PTSDKETSVA**L**NLITT O60260 : MIVFVRFNSSHGFPVEVDSDTSIFQLKEVVAKRQGVPADQLRVIFAGKELRNDWTVQNCDLDQQSIVHIVQRPWRKGQEMNATGGDDPRNAAGGCEREPQSLTRVDLSSSVLPGDSVGLAVILHTDSRKDSPPAGSPAGRSIYNSFYVYCKGPCQRVQPGKLRVQCSTCRQATLTLTQGPSCWDDVLIPNRMSGECQSPHCPGTSAE**F**FF**KC**GA**H**PTSDKETSVA**L**HLIAT

Check type

Quality Z-score

Comment

Dihedrals

1.629

Optimal

Packing 1D

-1.020

Satisfactory

Packing 3D

-1.740

Satisfactory

Overall

-0.971

Good

: CEEEEECCCCCEEEEEECCCCCHHHHHHHHHHHHCCCCCCEEEEECCCCCCCCCCCCCCCCCCCCEEEEEECCCCCCCCCCCCCCCCCCCCCCCCCCCCCCCCCCCCCCCCCCCCCCCCEEEECCCCCCCCCCCCCCCCCCCCCCCCCCCCCCCCCCCCCCCCCCCCCCCCCCCCCCCCCCCCCCCCCCCCCCCCCCCCCCCCCCCHHHHHCCCCCCCCCHHHHHHHCCCCCCCC

**Target Match Template**: MIVFVRFNSSHGFPVEVDSDTSIFQLKEVVAKRQGVPADQLRVIFAGKELRNDWTVQNC**D**LDQQSIVHIVQRPWRKGQEMNATGGDDPRNAAGGCEREPQSLTRVDLSSSVLPGDSVGLAVILHTDSRKDSPPAGSPAGRS.............................................................................................. SecStr : CCEEEEEECCCCCCEEETTTTHHHHHHHHHHHHHHCTTTEEEEEETTEECTTTTGGGGGCCTTTTCTTTTCCCCCCCHHHHHHHHCHHHHHHHHHHHHHHHHHHHHHCCCCTTTTCCCEEEEECCCCCCCCCCCCCCCCCC.............................................................................................. D3JZW5 : MIVFVRFNSSHGFPVEVDSDTSIFQLKEVVAKRQGVPADQLRVIFAGKELRNDWTVQNC**D**LDQQSIVHIVQRPWRKGQEMNATGGDDPRNAAGGCEREPQSLTRVDLSSSVLPGDSVGLAVILHTDSRKDSPPAGSP.................................................................................................. B9VH11 : MIVFVRFNSSHGFPVEVDSDTSIFQLKEVVAKRQGVPTDQLRVIFAGKELRNDWTVQNC**D**LDQQSIVHIVQRPRRKGQEMNATGGDNARNTAGGCEREPQSLTRVDLSSSVLPGDSVGLAVILHTDSRNDSPPAGSP.................................................................................................. UPI00017: ..VFVRFNSSHGFPVEVDSNTSIFQLKEAVAKRQGVPADQLRVIFAGKDLRNDLTVQSC**D**LDQQSIVHVVLRPQRKDQETNTPGGDKPQSA.GGSEREPESLTRVDLSSSILPTHSVGLAVILNSDCKNDVPPPGRPAGRS.............................................................................................. D3JZW3 : MIVFVRFNSSYGFPVEVDSDTSIFQLKEVVAKRQGVPADQLRVIFAGKELQNHLTVQNC**D**LEQQSIVHIVQRPQRKSHETNASGGDKPQSTPEGSIWEPRSLTRVDLSSHILPADSVGLAVILDTDSKSDSEAARGPA................................................................................................. Q1WDP3 : MIVFVRFNSSHGFPVEVDSDTSIFQLKEVVAKRQGVPADQLRVIFAGKELRNDLTVQRC**D**LDQQSIVHVVLRPQRNGQERGVAAGHRP........REPASLTRVDLSGSVLPGDAVGLAVILQDDSADGAAPAGRPADR............................................................................................... B8YP90 : MIVFVRFNSSYGFPVEVDSDTSILQLKEVVAKRQGVPADQLRVIFAGKELPNHLTVQNC**D**LEQQSIVHIVQRPRRRSHETNASGGDEPQSTSEGSIWESRSLTRVDLSSHTLPVDSVGLAVILDTDSKRDSEAARGPA................................................................................................. Q9WVS6 : MIVFVRFNSSYGFPVEVDSDTSILQLKEVVAKRQGVPADQLRVIFAGKELPNHLTVQNC**D**LEQQSIVHIVQRPRRRSHETNASGGDEPQSTSEGSIWESRSLTRVDLSSHTLPVDSVGLAVILDTDSKRDSEAARGP.................................................................................................. UPI0000E: MKVFVRFNSNHGFPVEVDSDTSIFQLKEVVARRQGVPADQLCVIFAGKELRNDWTVQSC**D**LDQQSIVHIVLRPRRKGPE........PRPAWGRSDREPESLTRVDLSSSMLPADSVGLAVILQDGEESGASSARRPAGR............................................................................................... UPI0001D: MKVFVRFNSNHGFPVEVDSDTSIFQLKEVVARRQGVPADQLCVIFAGKELRNDWTVQSC**D**LDQQSIVHIVLRPRRKGP......GHSPRPAWGRSDREPESLTRVDLSSSMLPADSVGLAVILQDGEESGASSARRPAGR............................................................................................... D3JW61 : MKVFVRFNSNHGFPVEVDSDTSIFQLKEVVARRQGVPADQLCVIFAGKELRNDWTVQSC**D**LDQQSIVHIVLRPRRKG..........PRPAWGRSDRELESLTRVDLSSSVLPADSVGLAVILQDGEESGASSARRPAGR............................................................................................... D2Y181 : MKVFVRFNSNHGFPVEVDSDTSIFQLKEVVARRQGVPADQLCVIFAGKELRNDWTVQSC**D**LDQQSIVHIVLRPRRKGPE.......SPRPAWGRSDRELESLTRVDLSSSVLPADSVGLAVILQDGEESGASSARRPAGR............................................................................................... UPI00015: MQVFVRFNSCHGFPVEVDSDTSIFELKELVAKQQGVPADQLRVIFAGKELRNDLTLQNC**D**LEQQSIVHVVRRIKDGGQEVGEATPAKLATTVGSLCREPKSLTRVDLSNTLLPGTAVGLAVILDDTSENDNAPSGKASGRS.............................................................................................. F1MA69 : .LVFVRFNSSYGFPVEVDSDTSIFQLKEVVAKRQGVPADQLRVIFAGKELQNHLT..NC**D**LEQQSIVHIVQRPQRKSHETNASGGDKPQSTPEGSIWEPRSLTRVDLSSHILPADSVGLAVILDTDSKSDSEAARGPEAK............................................................................................... UPI00019: ..VFVRFNSSHGFPVEVGSDSSILQLKEAVAQRQGVPADQLRVIFAGRELSNDLTLQNC**D**LAQQSIVHIVESPQKNSQDKEKTEYSCVGGVPKALKREPESLTRIDLSTSILPSVSAGLAVI....PGKNMPFADSPASRA.............................................................................................. F1NWU0 : ..VFVRFNSSHGFPVELGLDASILQLKEAVAQRQGVPADQLRVIFAGRELSNDLTLQNC**D**LVQQSIVHIVQ.........D.............LERVPESLTRIDLSSSILPSLSAGLAVILDTKEPNISPPSEKSAG................................................................................................ UPI00020: ..VFVRFNSSHGFPVEIDSDTSIFQLKEAVAKRQGVPADQLRVIFAGKELRNDLTVQSC**D**LPQQSIVHVIQTPQKK............................................................................................................................................................... D3K2X1 : MIVFVRFNSSHGFPVEVDSDTSIFQLKEVVAKRQGVPADQLRVIFAGKELRNDWTVQQV**D**QSTTAFMCIAKAPVKECSREN.......................................................................................................................................................... C3XVY8 : MQVFVRFNSHHSFPVDVDSSWSVLQLKEVLAARQQVPPAEIRIIFAGRELRDSFIIGEC**D**LASHSIVHVV..........................QEPQSLTRVDL................................................................................................................................ UPI00016: ..VFVRYNLGPEVVVELQEEATVAELKEVVGQQQGVQPDLLRVLFAGRELKSTSTLQGC**D**LPEQSTVH...................PEHLSQGEEENHDSLTRLDLSASRLPTTSSTLGVIL................................................................................................................ UPI00016: ..VFVRYNLGPEVVVELQEEATVAELKEVVGQQQGVQPDLLRVLFAGRELKSTSTLQGC**D**LPEQSTVH...................PEHLSQGEEENHDSLTRLDLSASRLPTTSSTLGVIL.....NDSEGVGATAG................................................................................................

**NOTE:** To save space, only 20 of 36 template profile alignments are shown above. The complete set of alignments can be found in the file humanParkin-Aug2013-TRC_t002_profile.ali.

In the alignment above, 141 of 465 target residues (30.3%) are aligned to template residues. Among these aligned residues, the sequence identity is 100.0% and the sequence similarity is 100.0% ('similar' means that the BLOSUM62 score is > 0). The following 1 loop had to be modeled:

SecStr

: MIVFVRFNSSHGFPVEVDSDTSIFQLKEVVAKRQGVPADQLRVIFAGKELRNDWTVQNCDLDQQSIVHIVQRPWRKGQEMNATGGDDPRNAAGGCEREPQSLTRVDLSSSVLPGDSVGLAVILHTDSRKDSPPAGSPAGRSIYNSFYVYCKGPCQRVQPGKLRVQCSTCRQATLTLTQGPSCWDDVLIPNRMSGECQSPHCPGTSAE**F**FF**KC**GA**H**PTSDKETSVA**L**HLIAT **:** MIVFVRFNSSHGFPVEVDSDTSIFQLKEVVAKRQGVPADQLRVIFAGKELRNDWTVQNCDLDQQSIVHIVQRPWRKGQEMNATGGDDPRNAAGGCEREPQSLTRVDLSSSVLPGDSVGLAVILHTDSRKDSPPAGSPAGRS

Loop

N-terminal anchor

Loop sequence

C-terminal anchor

1

PAGRS

IYNSFYVYCKGPCQR

None

E

. . . . . . . . . . . . . . . . . . . . . .

After the side-chains had been built, optimized and fine-tuned, all newly modeled parts were subjected to a combined steepest descent and simulated annealing minimization (i.e. the backbone atoms of aligned residues were kept fixed to avoid potential damage).

The resulting half-refined model has been saved as humanParkin-Aug2013-TRC_t002-__refined050.yob and obtained the following quality Z-scores:

Check type Dihedrals Packing 1D Packing 3D Overall

Quality Z-score 0.589 -1.411 -1.402

-1.117

Comment Optimal Satisfactory Satisfactory Satisfactory

Then a full unrestrained simulated annealing minimization was run for the entire model. The result has been saved as humanParkin-Aug2013-TRC_t002-__refined100.yob, the corresponding Z-scores are listed below:

Quality Z-score 1.030 -1.122 -1.392

-0.936 Since the overall quality Z-score improved to -0.936 during the minimization, this fully refined model has been accepted as the final one for this template and alignment.

**NOTE:** 309 C-terminal residues have been skipped entirely and are not part of the model, as requested by the 'TermExtension' and 'LoopLenMax' parameters. Since YASARA does not yet perform ab initio structure prediction, it would not make sense to append very long tails. If a 'tail' happens to contain an entire domain, it can usually be found in another model and joined manually, provided that the 'Templates' parameter has been set large enough to include also the other template. Nevertheless 15 terminal residues were modeled, but excluded from the Z-scores in both tables above, to minimize noise from terminal regions.

The final model for this template and alignment has been saved as humanParkin-Aug2013-TRC_t002-_.yob, and is shown below, together with a plot of its overall quality Z-score, shown per residue:

**7. The model ranking**

The following table lists the 2 models sorted by their overall quality Z-scores. The models have been superposed and saved together as humanParkin-Aug2013-TRC.sce. Rank Z-score Structure State Model ID Filename Original number Residues Comment 1 -0.923 monomeric T001-_ humanParkin-Aug2013-TRC_t001-_.yob 1 141-465 Good

2 -0.936 monomeric T002-_ humanParkin-Aug2013-TRC_t002-_.yob 2 1-141 Good

**8. The hybrid model**

Finally, YASARA tried to combine the best parts of the 2 models to obtain a hybrid model, hoping to increase the accuracy beyond each of the contributors. The following fragments were copied from other models (**note** that the first transfer is simply the initial model considered most suitable for hybridization, and that the scores in the right column are not comparable to the Z-scores listed further above, since they now penalize missing and very exposed residues):

Transfer First residue Last residue Length From model Score 1 126 465 340 T001-_ **-3.444** 2 1 143 143 T002-_ **-1.710** accepted

The resulting hybrid model obtained the following quality Z-scores (this time the score includes floppy terminal tails):

Check type Dihedrals Packing 1D Packing 3D Overall

Comment Optimal Satisfactory Satisfactory Good

Comment Optimal Satisfactory Satisfactory

Overall The following figure shows the initial model in blue, and all hybridized parts in a different color.

Check type Dihedrals Packing 1D Packing 3D

Quality Z-score 1.374 -1.127 -1.706

-1.034

Satisfactory

This hybrid model with Z-score -1.034 was saved as the final one, humanParkin-Aug2013-TRC.yob
